# Bacterial expression of a designed single‐chain IL‐10 prevents severe lung inflammation

**DOI:** 10.15252/msb.202211037

**Published:** 2023-01-04

**Authors:** Ariadna Montero‐Blay, Javier Delgado Blanco, Irene Rodriguez‐Arce, Claire Lastrucci, Carlos Piñero‐Lambea, Maria Lluch‐Senar, Luis Serrano

**Affiliations:** ^1^ Centre for Genomic Regulation (CRG) The Barcelona Institute of Science and Technology Barcelona Spain; ^2^ Universitat Pompeu Fabra (UPF) Barcelona Spain; ^3^ ICREA Barcelona Spain

**Keywords:** infection, interleukin, live biotherapeutics, mycoplasma, protein engineering, Biotechnology & Synthetic Biology, Immunology, Microbiology, Virology & Host Pathogen Interaction

## Abstract

Interleukin‐10 (IL‐10) is an anti‐inflammatory cytokine that is active as a swapped domain dimer and is used in bacterial therapy of gut inflammation. IL‐10 can be used as treatment of a wide range of pulmonary diseases. Here we have developed a non‐pathogenic chassis (CV8) of the human lung bacterium *Mycoplasma pneumoniae* (*MPN*) to treat lung diseases. We find that IL‐10 expression by *MPN* has a limited impact on the lung inflammatory response in mice. To solve these issues, we rationally designed a single‐chain IL‐10 (SC‐IL10) with or without surface mutations, using our protein design software (ModelX and FoldX). As compared to the IL‐10 WT, the designed SC‐IL10 molecules increase the effective expression in *MPN* four‐fold, and the activity in mouse and human cell lines between 10 and 60 times, depending on the cell line. The SC‐IL10 molecules expressed in the mouse lung by CV8 *in vivo* have a powerful anti‐inflammatory effect on *Pseudomonas aeruginosa* lung infection. This rational design strategy could be used to other molecules with immunomodulatory properties used in bacterial therapy.

## Graphical abstract

Computer‐designed single‐chain IL‐10 molecules expressed in the mouse lung by an engineered attenuated *Mycoplasma pneumoniae in vivo* have a powerful anti‐inflammatory effect on *Pseudomonas aeruginosa* lung infection.

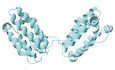

## Introduction

The IL‐10 cytokine has potent anti‐inflammatory properties and plays a central role in limiting the host immune response, thereby preventing damage to the host and maintaining normal tissue homeostasis. IL‐10 has potential applications as an immunotherapeutic agent in tumours, as it can boost CD8^+^ tissue T‐cell infiltration and increase IFN‐γ production, thereby stimulating the T‐cell memory response (Fillatreau & O'Garra, 2014). To date, however, clinical trials using IL‐10 have failed, in part due to the short half‐life of IL‐10 in the bloodstream, as well as its proinflammatory effects when applied systemically at high concentrations (van Deventer *et al*, 1997; Saxena *et al*, 2015). To improve its pharmacodynamics, IL‐10 has been fused to an IgG‐Fc region (Terai *et al*, 2009) or to polyethylene glycol (Duncan *et al*, 2019), and to dampen its pro‐inflammatory effects, novel systemically engineered variants have been generated that modulate its binding to the low‐affinity receptor 2 (R2; Gorby *et al*, 2020) or that do not activate the proinflammatory cascade (Saxton *et al*, 2021).

The use of bacteria as delivery vectors or live biotherapeutic product (LBP) has the advantage of being able to generate the therapeutic agent locally, which lowers the required administered dose, thereby reducing adverse effects and production costs. Recently, IL‐10 expressed by *Lactococcus lactis* has been shown to be effective in preventing and treating colitis in a preclinical mice model (Steidler *et al*, 2000; Cardoso *et al*, 2018) as well as in phase I clinical trials of Crohn's disease (Braat *et al*, 2006). In the lung, IL‐10 can also be used to prevent tissue damage in transplanted organs (Cypel *et al*, 2009) or as treatment of a wide range of pulmonary diseases, such as fibrosis (Shamskhou *et al*, 2019), asthma or acute respiratory distress syndrome (ARDS; Ouyang & O'Garra, 2019; Wang *et al*, 2019). Ideally, a bacterium used for therapy should be administered to the organ where it is naturally present, to ensure the survival and adaptation to the host niche. We have previously demonstrated the potential application of *Mycoplasma pneumoniae* (*MPN*) as an engineered vector for the treatment of *Staphylococcus aureus* biofilm *in vivo* (Garrido *et al*, 2021). Although *MPN* is a mild pathogen that causes pneumonia in humans, it has some advantages as a LBP, mainly related to the absence of a cell wall, which allows the secretion of molecules directly into the medium. Further, *MPN* also has intrinsic properties that limits horizontal gene transfer, such as its use of a UGA triplet as a tryptophan codon rather than a STOP codon (Inamine *et al*, 1990; Wodke *et al*, 2013). Further, some strains (i.e., *MPN* 129) have a weaker recombination capacity (Sluijter *et al*, 2010). Finally, it divides every 8–12 h, making it more easily controlled.

However, this bacterium has limited protein synthesis capacities as compared to *Escherichia coli* or *Bacillus subtilis*; for instance, it has 100 to 200 ribosomes per cell (Kühner *et al*, 2009; Maier *et al*, 2011) vs. 1 × 10^5^ ribosomes per cell in *E. coli* in the exponential phase (Scott *et al*, 2010). Thus, it is critical to optimise the effective concentration and activity of the expressed IL‐10 molecule. To address this, we first took into account the molecular characteristics of IL‐10 that distinguish it from other interleukins (ILs): it comprises a homodimeric swapped domain (Bennett *et al*, 1994) in which each monomer is bridged by Cys12‐Cys108 and Cys62‐Cys114 disulphide bonds (PDB residue numbering), but with no intermonomeric disulphide bonds. An engineered monomeric version created by enlarging a loop by inserting six amino acids (aa; e.g., GGGSGG) is significantly less active (Josephson *et al*, 2000), underscoring the importance of the dimeric folding of IL‐10 for its activity. However, this requirement for a dimeric state reduces the effectiveness of IL‐10 expressed and secreted by a bacterial chassis in a human organ, as dimerisation is hindered in large spaces, leaving the proteins more prone to dissociation at low concentrations and/or low pH (Syto *et al*, 1998), which could lead to degradation, aggregation and extensive multimerisation (Westerhof *et al*, 2012). This is especially important in the lung, which has a limited microbiome capacity as compared to the intestine (Huffnagle *et al*, 2017), preventing large amounts of bacteria from being administered.

Here, we (i) designed mutations in IL‐10 that increase its affinity for the R1 and R2 receptors (Gorby *et al*, 2020) and (ii) engineered a single‐chain (SC) variant composed of two IL‐10 monomers, using two protein design softwares: FoldX (Schymkowitz *et al*, 2005; Delgado *et al*, 2019) and ModelX (Blanco *et al*, 2018; Delgado Blanco *et al*, 2019; Cianferoni *et al*, 2020). *MPN* was able to express active IL‐10 and the new IL‐10 variants that exhibited an increased affinity as compared to the wild‐type (WT) IL‐10 (IL‐10 ORF) *in vitro* and had a powerful antiinflammatory effect in an acute lung infection model induced by *Pseudomonas aeruginosa*. This strategy could reduce production costs and be used in other therapeutic bacteria, as a more active product secreted by the bacterial chassis leads to a lower required bacterial dose and hence a reduction in any potential pathogenicity.

## Results

### *M. pneumoniae* secretes functional IL‐10 dimers with disulphide bridges

**Figure 1:**
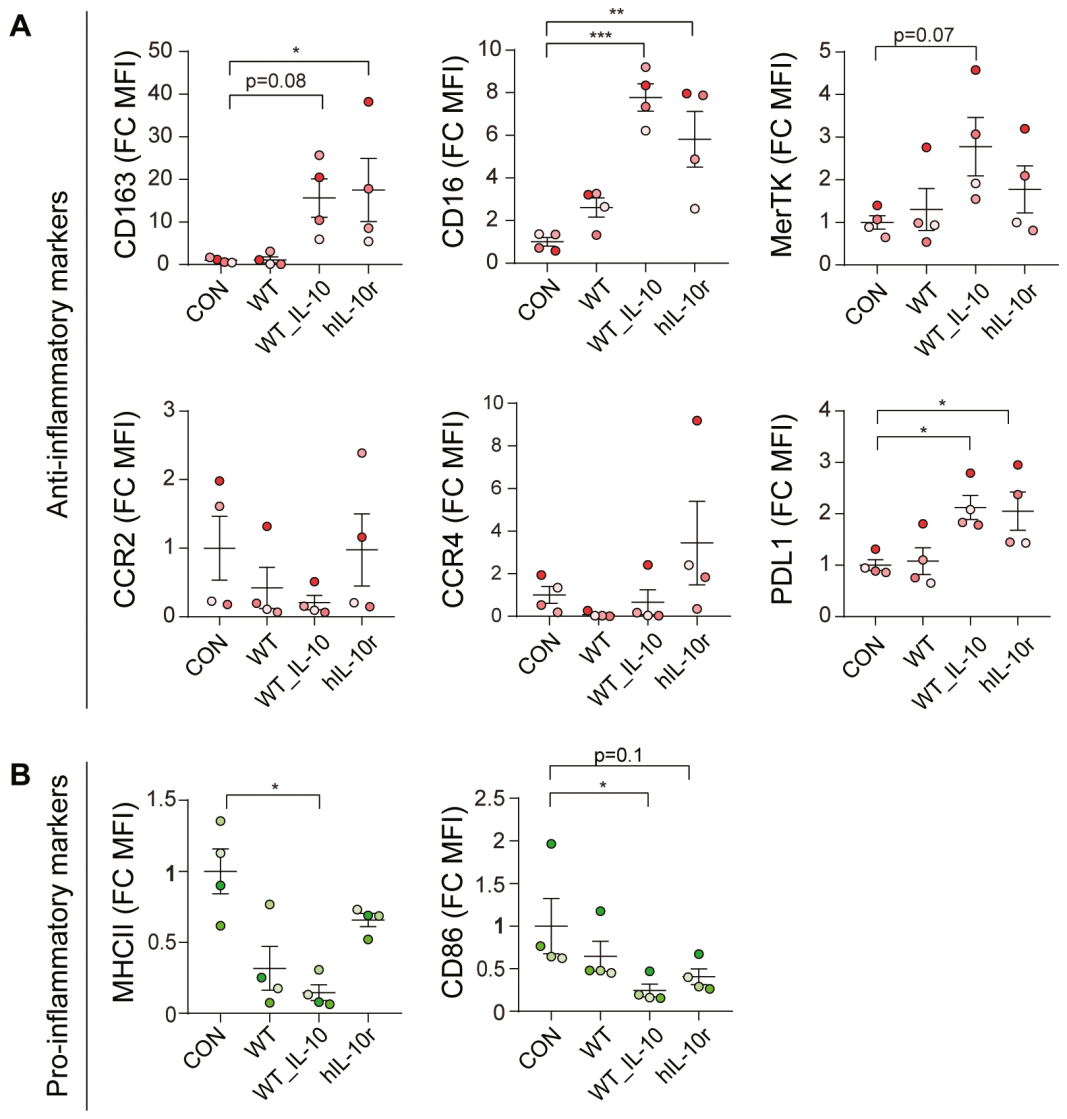
Effects of human IL‐10 recombinant protein (hIL‐10r) or IL‐10 secreted by *M. pneumoniae* on macrophage primary cells on anti‐inflammatory (A) or pro‐inflammatory biomarkers (B) A, BData were normalised using the control group and are represented as fold‐change (FC) of the mean fluorescence intensity (MFI). Macrophage cells were obtained from human donors (*n* = 4 biological replicas) and incubated with medium (CON) or recombinant human IL‐10 (hIL‐10r), *MPN* wild‐type (WT) or *MPN* expressing IL‐10 ORF (WT_IL10). Data are represented as mean ± standard error of the mean (SEM). Statistical analysis was performed using one‐way ANOVA + *post hoc* Tukey's multiple comparison test (*P < 0.05; **P < 0.001, ***P < 0.0001). Source data are available online for this figure.

**Figure EV1:**
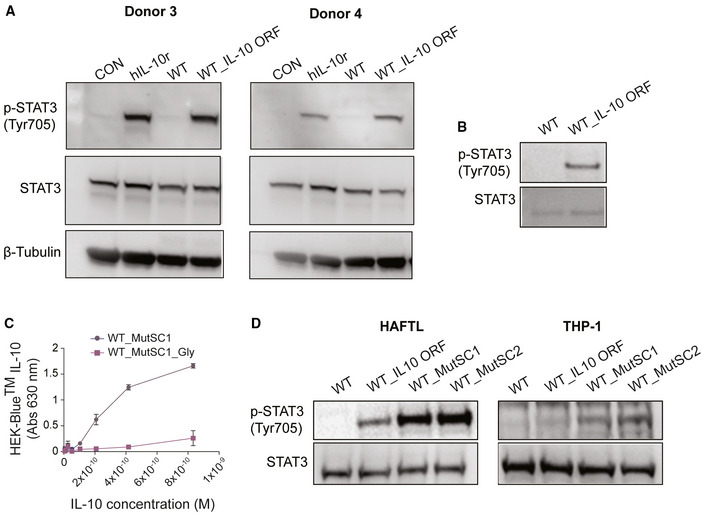
Analysis of SC mutants designed in this work A, BDetection of phosphorylated Tyr 705 of STAT3 (p‐STAT3) and unphosphorylated STAT3 by Western blot (see Materials and Methods). (A) Data from macrophages isolated from two independent donors, showing unstimulated cells (control, CON) or cells incubated for 24 h with human IL‐10 recombinant protein (hIL‐10r), supernatant of *MPN* WT (WT) or of *MPN* expressing IL‐10 (WT_IL‐10 ORF). (B) Data from HAFTL murine B‐cell line after exposure for 20 min to the supernatant of *MPN* WT (WT) or *MPN* expressing IL‐10 (WT_IL‐10 ORF).CHEK‐Blue™ reporter cell activation dose–response analysis by MutSC1 (linker NGGLD) and MutSC1_Gly (linker GGGGG) supernatants. The *x*‐axis shows the range of IL‐10 concentration analysed (Molar, M), and the *y*‐axis represents the mean ± SD of the absorbance at 630 nm. Data were generated in three independent assays with two technical replicas (*n* > 6).DWestern blot of p‐STAT3 activation after 20 min of induction with a fixed IL‐10 concentration (20 ng/ml) of supernatants from *MPN* WT (WT) or *MPN* expressing IL‐10 WT (WT_IL10 ORF), MutSC1 (WT_MutSC1), MutSC2 (WT_MutSC2) in two different cell lines: THP‐1 (human monocyte) and HAFTL (murine pre‐B cell line).

We first tested whether *MPN* can actively express a complex molecule, such as the human IL‐10‐swapped dimer with two disulphide bridges. For this purpose, we cloned the human IL‐10 fused to the *mpn142* secretion signal previously described for *MPN* (Garrido *et al*, 2021). We showed by ELISA that the *MPN* WT strain secreted IL‐10 in the supernatant (Table EV1). We confirmed by mass spectroscopy (MS) that both disulphide bridges were generated in the expressed IL‐10 (Dataset EV1), indicating that the dimer was correctly folded. We next tested the functionality of the produced IL‐10 (IL‐10 ORF) for its ability to modulate the primary anti‐inflammatory response activation program of human macrophages (see Materials and Methods). For this, *MPN* expressing IL‐10 was grown for 2 days, and the supernatant was added to a cell culture containing approximately one million circulating macrophages (see Materials and Methods). As a positive control, the human IL‐10 recombinant protein (hIL‐10r) was added. Commercial hIL‐10r treatment of macrophages enhanced the expression of anti‐inflammatory markers (Fig 1A) and decreased the levels of pro‐inflammatory ones (see Fig 1B) as compared to non‐treated cells. The results using IL‐10 ORF were comparable to those using hIL‐10r. We also confirmed that human primary blood CD14^+^ macrophages become activated upon hIL‐10r or IL‐10 ORF stimulation by phosphorylation of Tyr705 of STAT3 (p‐STAT3; Yu *et al*, 2009; Fig EV1A). Additionally, p‐STAT3 was activated in HAFTL murine B‐cell line (Fig EV1B), indicating that both the human and murine cell lines were activated by the human IL‐10.

In summary, these results demonstrated that *MPN* expresses functional human IL‐10 that is capable of activating phosphorylation of STAT3 in human primary blood CD14^+^ macrophages and in a murine cell line.

### Engineering IL‐10 for increased receptor affinity

**Figure 2:**
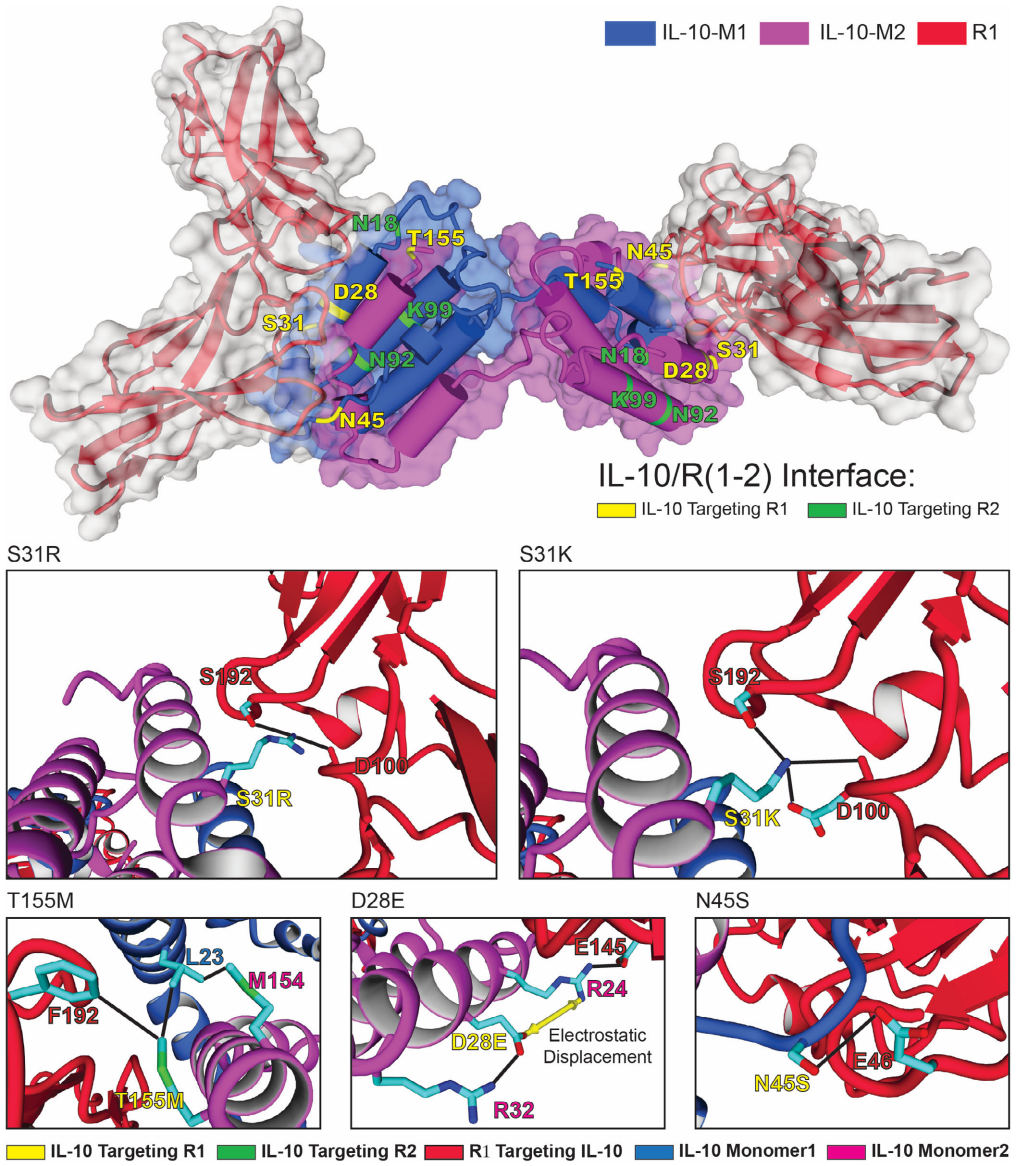
Schematics of all mutations generated in this work to increase IL‐10/R1/R2 receptor affinity Details for each of the positions mutated to improve interactions with R1 are shown in the individual panels. Note that when aspartic acid‐28 is mutated to glutamate in IL‐10, an electrostatic displacement occurs over the arginine‐24, allowing it to form an H‐bond with glutamate‐145 from the receptor 1 (yellow double arrow in the subfigure indicates D28E).

**Figure EV2:**
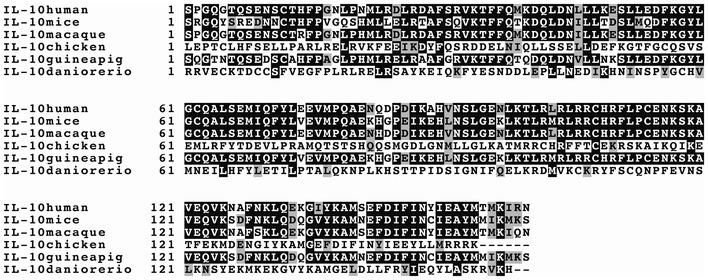
Multiple sequence alignment of IL‐10 from different vertebrate species performed with the ClustalX algorithm The first residue corresponds to the first residue of IL‐10 in the crystal structure 1y6k.

To overcome the limitations of a low protein production capacity in *MPN*, and to decrease the number of bacterial cells administered to a patient, one strategy is to engineer mutations in IL‐10 with increased binding affinity to the high‐ and low‐affinity receptors R1 and R2, respectively. To generate IL‐10 versions with an increased affinity towards R1, we used the hIL‐10 molecules crystallised in complex with R1 with the best crystal resolution (PDB 1y6k; 2.5 Å). We then performed an *in silico* mutagenesis scanning of the complex interface residues using the *PositionScan* FoldX command, which individually mutates all R1‐contacting IL‐10 aa to the other 19 natural aa (Schymkowitz *et al*, 2005). Based on this analysis and on sequence conservation across different species (Fig EV2), we identified a set of non‐conserved residues that, when mutated with FoldX, improved binding to R1 without compromising protein stability (Dataset EV2). Using the selected mutations, we modelled two multiple mutants, termed Mut1 and Mut2, that contain D28E, S31K/R, N45S and T155M (note that they differ at position 31, with the WT Ser31 mutated to Lys in Mut1 and Arg in Mut2; Fig 2). We verified that the differences in stability and binding energies were not the result of a particular conformation of the crystal structure that we used by computing the *in silico* variation of free energies of the multiple mutations of IL‐10 in Mut1 and Mut2 using other experimental structures (IL‐10 apo form: PDB 2ilk, 1.6 Å; IL‐10 holo form with R1: PDB 1j7v, 2.9 Å; IL‐10 holo form with R1&R2: PDB 6x93, 3.5 Å). We looked for changes in energy that affected IL‐10 stability, interactions with R1 and R2 (when present in the PDB structure) and stability of IL‐10 receptor complex (Dataset EV2).

**Figure EV3:**
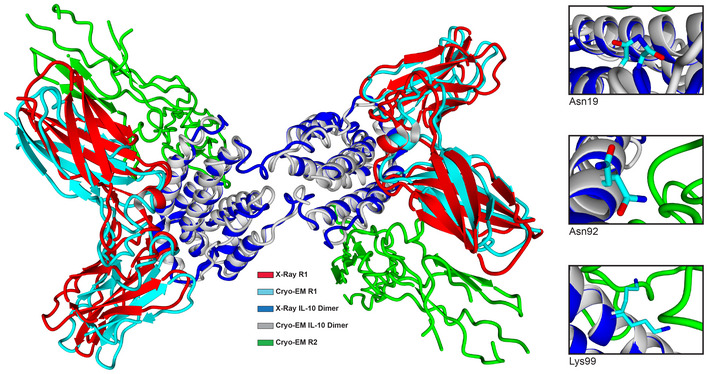
Superimposition of the crystal structures of IL‐10 with R1 (1yl6k) and of IL‐10 with both R1 and R2 (6x93) Unstructured regions adopted a different conformation when interacting with R2. Gorby (9)‐mutated positions (N19, N92, K99) are zoomed‐in on the right side in the crystallographic superimposition with cryo‐EM (9), showing an appreciable backbone shifting upon binding to R2 in some of the positions.

At the start of this work, no structure was available for IL‐10 bound to R2. Therefore, we included mutations in Mut2 that had been previously reported to potentially enhance the affinity of the interactions between IL‐10 and R2 (e.g., N18I, N92I and K99N; Gorby *et al*, 2020), resulting in Mut3. When modelling these three mutations in X‐ray IL‐10 structures in a complex with the R1 but without the R2 receptor (1y6k, 2ilk), we found that they decreased the stability of IL‐10 (Dataset EV2). This apparent decrease in stability when using the 1y6k and 2ilk structures is due to a conformational change in the regions where the mutations are introduced upon binding to the R2 receptor (see the X‐ray; 1y6k, 2ilk) superimposed on a cryo‐EM structure (6x93; 3.5 Å) of the complex between IL‐10 and R1/R2 (Saxton *et al*, 2021; Fig EV3). In fact, two of these previously described mutations (N18I and N92I) modelled on the 6x93 improved stability and binding to R2 (Dataset EV2).

### Measurement of EC‐50 and relative affinity ratios for the designed multiple mutants

To assess the relative affinity of the different designed IL‐10 variants, we used a reporter cell line (HEK‐Blue™) that contains a secreted embryonic alkaline phosphatase (SEAP) reporter. Briefly, when IL‐10 is bound to R1 and R2, the SEAP reporter triggers a JAK1/STAT3‐mediated response resulting in SEAP expression, which can be measured by absorbance at 630 nm (see Materials and Methods). The intensity of the signal before saturation is proportional to the activation of STAT3, and a kinetic model (Heck, 1971) can be fitted to the data to obtain EC‐50 values (see Materials and Methods), which could be used to determine relative activity differences between WT and mutants (Gorby *et al*, 2020). Mutants Mut1 and Mut2 (designed for improved binding to R1) had similar EC‐50 values (1.45 e^−10^ M ± 6.59 e^−11^ and 6.45 e^−11^ ± 1.73 e^−11^ M, respectively), but were better than IL‐10 ORF (1.98 e^−10^ ± 9.2 e^−11^ M; 1.4‐fold and 3.0‐fold enhancement in relative affinity, respectively). Mut3, based on Mut2, incorporates new mutations previously described to improve R2 binding (namely, N18I, N92I and K99N; Gorby *et al*, 2020). Inclusion of these three mutants resulted in an improved EC‐50 value (2.61 e^−11^ ± 1.49 e^−11^ M), with 8‐fold enhancement in relative affinity as compared to the IL10 ORF (Fig 4A). This increased affinity is to be expected, as it contains mutations that enhance both R1 and R2 binding, while Mut2 only has mutations to enhance R1 binding. We showed that the EC‐50 of the monomeric IL‐10 (MutM) is on average lower (although not significant statistically; *P* > 0.05) than that of the IL‐10 ORF (2.56 e^−10^ ± 8.77 e^−11^), as previously described (Josephson *et al*, 2000; Fig 4A and Dataset EV3). The expression level in *MPN* of the best mutant (Mut3) was comparable to that of the IL‐10 ORF (Fig 4B). Thus, we observed that mutations incorporated in Mut3 enhanced the relative activity by about 8‐fold as compared to that of the IL‐10 ORF.

### Single‐chain IL‐10 rational design

**Figure 3:**
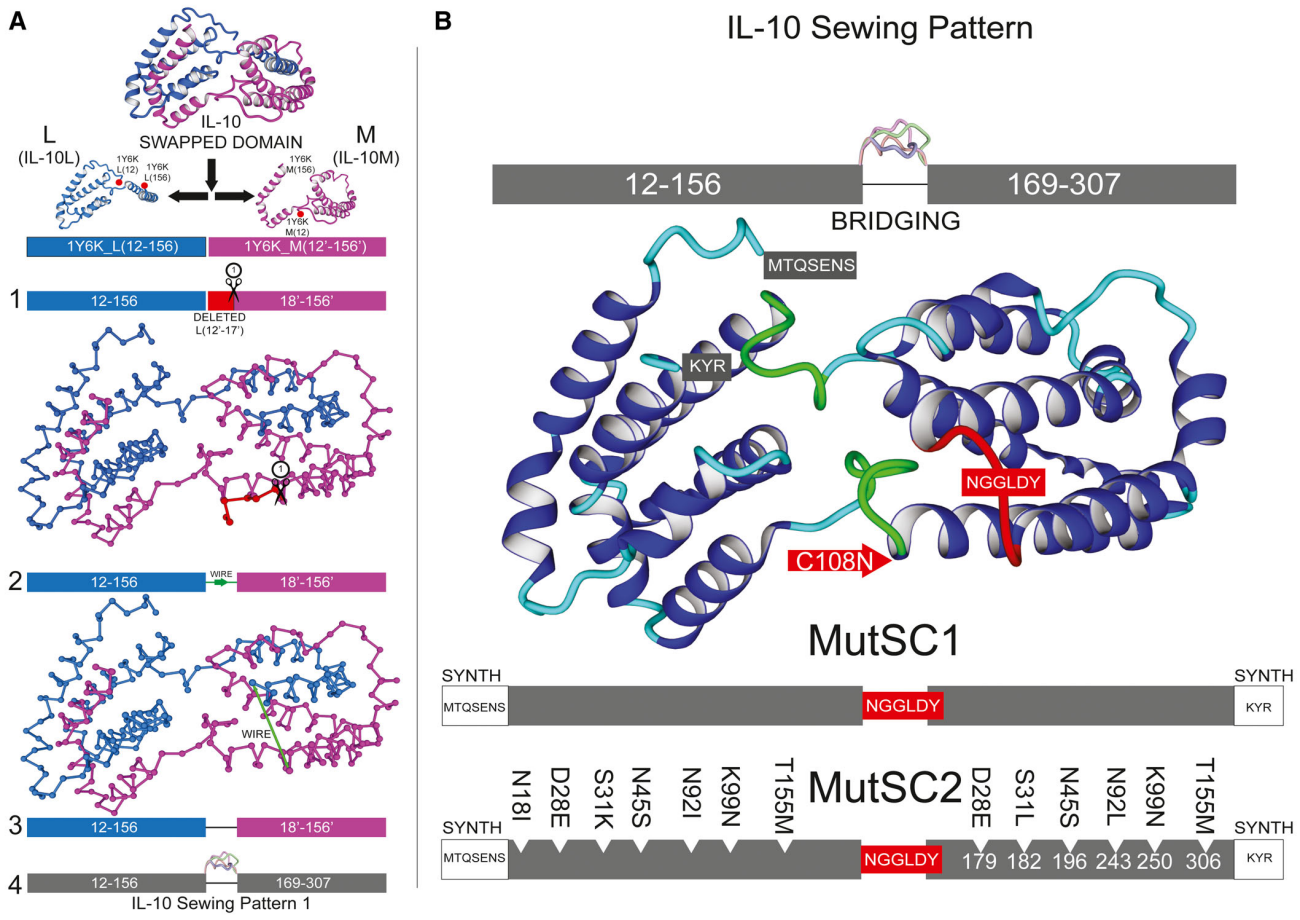
Schematic depiction of the design of single‐chain (SC) IL‐10s IL‐10L and IL‐10M monomers are respectively shown in blue and magenta. To avoid confusion, IL‐10M residue numbers are denoted by adding the prime (′) character.
Steps to generate two different sewing patterns: (1) deletion of fringe residues; (2) rewiring schema; (3) structural rearrangement after rewiring the corresponding regions; (4) final numbering in the SC, including numerical gaps long enough to host peptide bridges with different lengths (up to 20) during linker search or *bridging*. Monomer 2 (IL‐10M, in magenta) residue numbers are marked by '.MutSC1 and MutSC2 built from the IL‐10 sewing pattern 1. Linker sequences are shown as red labels; post‐rewiring mutations are included for MutSC2 using monomeric IL‐10 numbering.

Active IL‐10 is a swapped dimer, with no intramonomeric disulphide bonds, composed of IL‐10L and IL‐10M monomers (Fig 3A). However, reaching an equilibrium between the unfolded monomer and the folded dimer decreases effective concentration and could result in potential degradation, misfolding and/or extensive multimerisation (Westerhof *et al*, 2012). To solve this problem, we decided to create a SC protein by linking the N‐terminal region of monomer M with the C‐terminal region of the monomer L in the swapped dimer (MutSC1; Fig 3A). To design the linker, we used the ModelX software (Bridging command) developed by our group to screen for linkers *in silico* between Asn18' from monomer M and Lys157 from monomer L as anchoring points (see Materials and Methods). Bridging connected the anchoring points with all geometrically compatible peptide fragments from PepXDB (see Materials and Methods). Fragments were retrieved with their corresponding side chains, allowing us to explore both conformational and sequence space at a time. We obtained 541 models that were side chain‐repaired and ranked by energy using the FoldX v5 (Delgado *et al*, 2019) RepairPDB and Stability commands, respectively (see Materials and Methods). We determined that the best linker sequence was N157‐FGGLD‐Y18', whereby Asn18' was replaced by Tyr and Lys157 by Asn, and the FGGLD sequence was inserted. By introducing this linker, we deleted the N‐terminal residues of M in the crystal structure (residues 12′ to 17′; CTHFPG). Afterwards, we mutated the Phe after N157 to Asn to improve binding to R1 (this results in NGGLD as the inserted sequence; Fig 3B); this improved the FoldX predicted overall stability of the SC molecule by creating an intramolecular HBond with G160 that rigidified the inserted loop as well as its binding to R1 by stabilising the bound state. Afterwards, we renumbered the sequence with the inserted connecting loop (see Materials and Methods). Note that we deleted the N‐terminal sequence of one of the monomers containing Cys12', which makes a disulphide bridge with Cys108' in the original IL‐10 ORF sequence (new residue 259); therefore, we mutated Cys108' to Asn, to prevent spurious disulphide bridges. The original Cys12 and Cys108 remained in the structural equivalent of the other monomer of the swapped dimer. Thus, MutSC1 has three of the four original disulphide bridges. MutSC2 was designed by grafting the Mut3 mutations into MutSC1 (Fig 3B). We verified by MS that both MutSC1 and MutSC2 generated the two different disulphide bridges present in the original monomers of the swapped structure (Dataset EV1).

### Characterisation of the engineered IL‐10 mutants *in vitro* in human and murine cell lines

**Figure 4:**
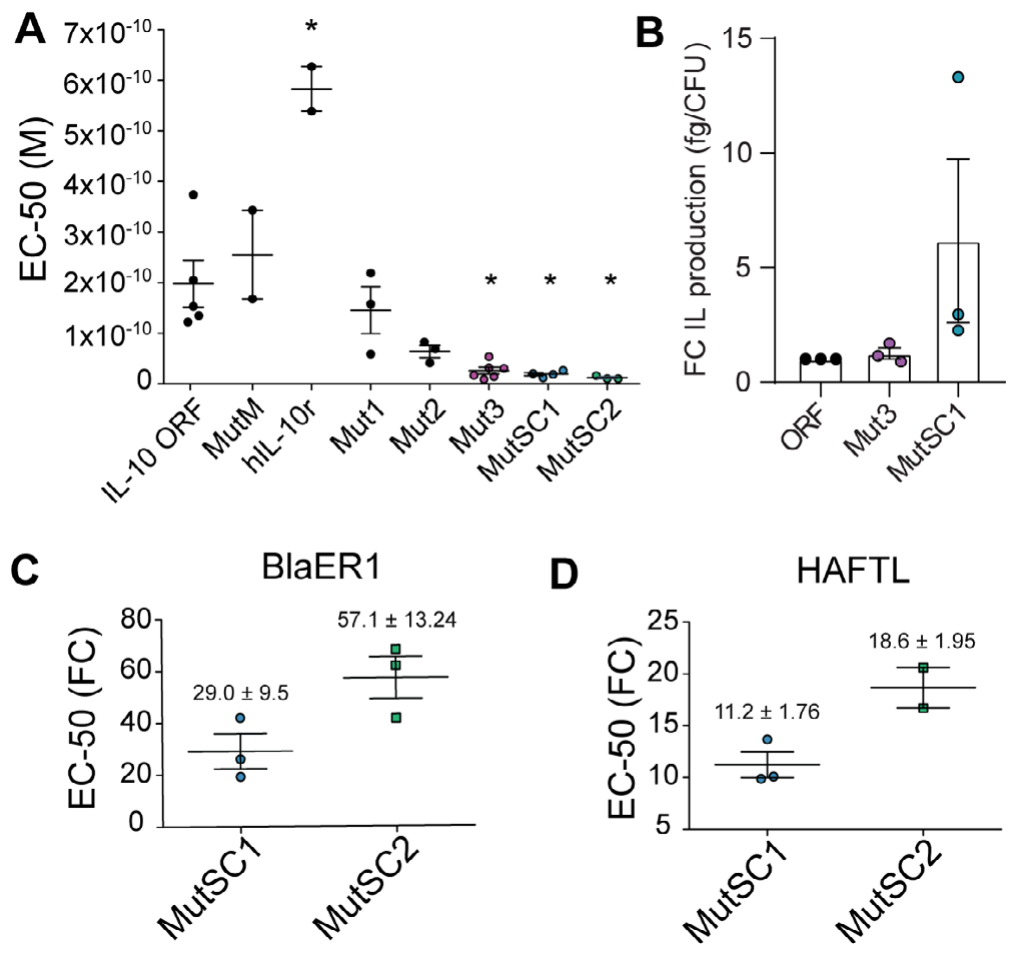
Expression levels and apparent dissociation constant of selected IL‐10 variants expressed by *M. pneumoniae* Each point in the figure is a biological replica.
AEC‐50 (molar, M) for human IL‐10 recombinant protein (hIL‐10r) and IL‐10 WT (IL‐10 ORF) and different variants (MutM, hIL‐10r, Mut1, Mut2, Mut3, MutSC1 and MutSC2) expressed by *M. pneumoniae*. Data are represented as mean ± SD. Statistical comparison was done by one‐way ANOVA + *post hoc* Bonferroni multiple comparison test, using the IL‐10 ORF condition as a reference (*P < 0.05).BFold‐change (FC) in expression levels (fg/CFU) of IL‐10 variants Mut3 and MutSC1 secreted to the medium normalised by expression level of IL‐10 ORF (ORF). Statistical comparison of mean ± SD was performed by one‐way ANOVA + Tukey's *post hoc* test (*P < 0.05).C, DAverage and SD values for the FC in the relative EC‐50 values determined by flow cytometry analysis of phosphorylated STAT3 after a 20‐min exposure of the BlaER1 (C) or HAFTL (D) cell lines to IL‐10 ORF (reference) or the mutant MutSC1 or MutSC2 (see Materials and Methods). Numbers indicate the average FC ± SD.
Source data are available online for this figure.

We next addressed whether expressing a SC‐IL10 rather than the swapped dimer increased the amount of functional protein expressed by *MPN*. We quantified the IL‐10 protein concentration in the cell culture supernatant by ELISA and determined the number of cells by counting colony‐forming units (CFU; see Materials and Methods). We found that the supernatant with MutSC1 contained up to 4‐fold more secreted IL‐10 protein than the supernatant with IL‐10 ORF or Mut3 (Fig 4B). We then assessed the capacity of each mutant to activate the IL‐10 receptors in HEK‐Blue™ cells. As compared to IL‐10 ORF (EC‐50 1.98 e^−10^ ± 9.24 e^−11^ M), the SC mutants MutSC1 (1.90 e^−11^ ± 5.12 e^−12^ M) and MutSC2 (1.20 e^−11^ ± 2.8 e^−12^ M) had better EC‐50 values than the IL‐10 ORF, with an improved relative activity of 10.4‐ and 16.5‐fold, respectively. Importantly, MutSC2 had a better EC‐50 than the best multiple mutant in the swapped dimer (Mut3). To determine the importance of the linker sequence in the activity of MutSC1 and MutSC2, we replaced the linker sequence **N**‐NGGLD‐**Y** with **N**‐GGGGG‐**Y** (MutSC1_Gly). The new control mutant MutSC1_Gly had a very significant decrease in activity, indicating that it is important for a short linker to have a specific sequence (Fig EV1C). To address whether this improvement in the relative activities of MutSC1 and MutSC2 was specific to the species cell type used in the assay, we assessed STAT3 phosphorylation (p‐STAT3) upon ligand stimulation by FACS in two different cell lines: murine HAFTL (a mouse pre‐B cell line) and human BLaER1 (a B‐cell precursor leukaemia cell line). As compared to IL‐10 ORF, both the MutSC1 and MutSC2 mutants were significantly superior in activating p‐STAT3 (enhanced relative activity: 29.0 ± 9.5‐fold and 57.1 ± 13‐fold in BLaER1; 11.2 ± 1.8‐fold and 18.6 ± 1.9‐fold in HAFTL, for MutSC1 and MutSC2, respectively; Fig 4C and D, and Dataset EV4). Altogether, the engineered mutants MutSC1 and MutSC2 resulted in enhanced relative activity in different cell types of human or mouse origin. As observed for the HEK‐Blue™ cells, MutSC2 was around 100% better than MutSC1. Similar qualitative results were found by Western blot in murine HAFTL and in the human monocyte cell line THP‐1 (Fig EV1D).

Overall, these results support the idea that the IL‐10 swapped homodimer produced by *MPN* is a mixture of unfolded monomeric and active dimeric forms. Thus, converting a swapped dimer into a SC molecule is an effective strategy for increasing the amount of not only secreted protein but also secreted active protein in bacteria. The engineered SC molecules are effectively recognised by human and murine IL‐10 receptors.

### Characterisation of the CV8 chassis strain *in vivo*

*MPN* induces an inflammatory response in the lungs of mice (Waites & Talkington, 2004; Tamiya *et al*, 2021). To solve this issue, we engineered an attenuated strain, referred to here as chassis CV8 (patent application EP 20382207.7). This chassis contains (i) deletion of the nuclease MPN133 (*mpn133*; Somarajan *et al*, 2010) and the CARDs toxin (*mpn372*; Segovia *et al*, 2015), and (ii) replacement of the glyceraldehyde‐3‐phosphate dehydrogenase GlpD (*mpn051*; Hames *et al*, 2009) with GpsA (see Materials and Methods), an enzyme with similar metabolic activity but which produces H_2_O rather than H_2_O_2_.

**Figure 5:**
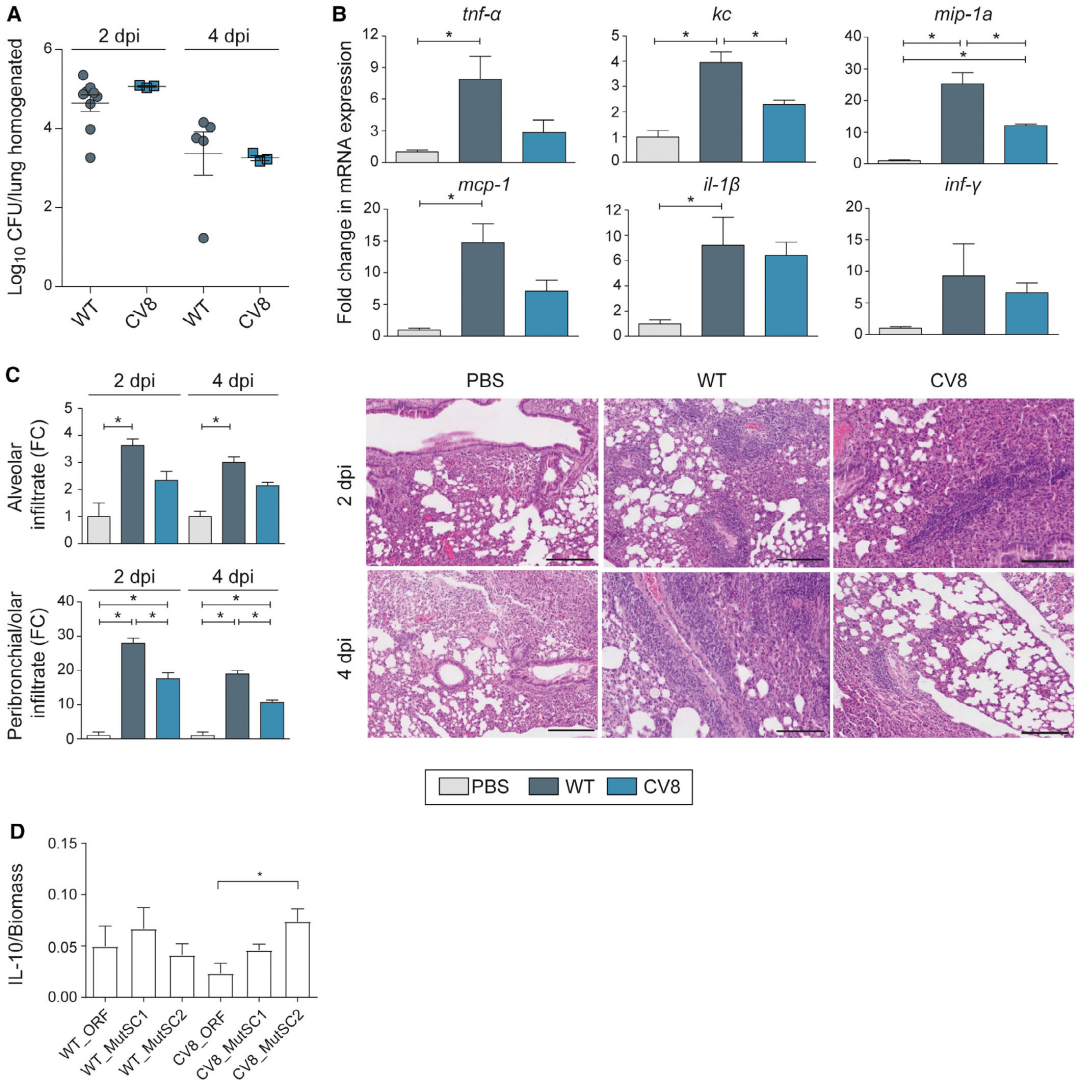
Characterisation of CV8 chassis *in vivo* C57Bl/6 were infected with 10^7^ CFU of WT or CV8 strains and sacrificed at 2 days post‐infection (dpi) or 4 dpi (*n* ≥ 3 biological replicas/group).
Average ± SD bacterial loads recovered of the WT (circle) and CV8 (square) strains in mouse lungs at 2‐ or 4‐dpi. Data are shown as log_10_ CFU/lung homogenate.Inflammatory profile of mouse lungs inoculated with WT (dark blue), CV8 (light blue) or PBS (grey). Gene expression was analysed by RT–qPCR using *gapdh* as an endogenous control (see Materials and Methods). Data are shown as average ± SD of fold‐change (FC) in mRNA expression (one‐way ANOVA + Tukey's *post hoc* test; *P < 0.05).Histological findings of mouse lung samples at the analysed time points. Left, plots representing the quantitative evaluation (score: 0–5) of alveolar infiltrate (top panel) and peribronchial/solar infiltrate (FC; bottom panel; see Materials and Methods). Parameters were normalised using the average of the PBS group (FC = sample value/average PBS; FC control group, ~1). Data are shown as average ± SD of FC (one‐way ANOVA + Tukey's *post hoc* test; *P < 0.05). Right, representative images of lungs stained with haematoxylin–eosin (the line represents 250 μm).IL‐10 secretion by the *MPN* WT or CV8 strain coding for hIL‐10 WT (ORF), MutSC1 or MutSC2. Data are expressed as the mean ± SD of IL‐10 supernatant concentration (μg/ml) by biomass (protein content in μg/ml; *n* = 3 biological replicas).
Source data are available online for this figure.

The virulence of CV8 was tested in a mice model of intratracheal lung infection (Fig 5). Similar bacterial loads were observed for the WT and CV8 strains at both time points analysed (Fig 5A), suggesting that deletion of the virulence factors did not compromise the colonisation capacity of the CV8 chassis strain. The lung inflammatory profile was analysed by RT–qPCR (Fig 5B) at 2 days post‐infection (dpi) and 4 dpi (Fig 5B). While the overall inflammatory response was not high, it was higher at 2‐ than 4 dpi, coinciding with the higher bacterial load in the lung at this time (Fig 5A). At 2 dpi, the WT strain increased the expression levels of *tnf‐a*, *kc (il‐8)*, *mip‐1a*, *mcp‐1*, and *il‐1b* (for all, *P* < 0.05; Fig 5B). For CV8, only the expression of *mip‐1a* changed significantly as compared to the PBS control (*P* < 0.05), but it was still lower than in the WT strain.

To confirm that the chassis was attenuated, we analysed the histological findings in lungs (Fig 5C). The WT strain promoted the presence of alveolar and peribronchial/peribronchial infiltrate at both 2‐ and 4‐dpi (*P* < 0.05 for both), as compared to the PBS control. In the CV8‐infected lungs, we also observed peribronchial/peribronchial infiltrate, but at a lower level than that observed in the WT strain at both time points analysed (*P* < 0.05 for both).

Altogether, these results confirmed the attenuated phenotype of the CV8 strain. We therefore generated the strains CV8_IL‐10 ORF (expressing IL10 ORF), CV8_MutSC1 (expressing MutSC1), and CV8_MutSC2 (expressing MutSC2), to test their immunomodulatory effects *in vivo*. No differences in protein capacity were observed between WT‐expressing and CV8‐expressing strains (Fig 5D).

### Engineered IL‐10 variants exhibit powerful immunomodulation effects *in vivo*

**Figure EV4:**
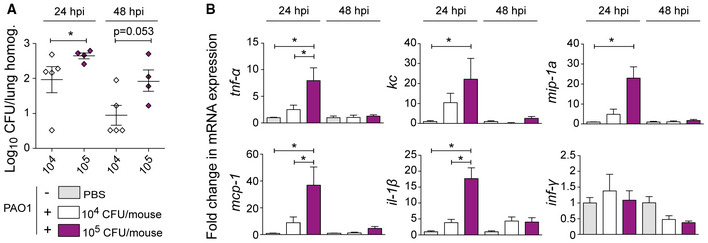
Analysis of *P. aeruginosa* PAO1 infection of mice lungs *Pseudomonas aeruginosa* PAO1 bacterial load obtained from mice infected with 10^4^ or 10^5^ CFU at 24‐ or 48 h post‐infection (hpi). Data are shown as mean ± SD of Log_10_ CFU/lung homogenate of at least 3 mice per group (*n* < 3 biological replica). Statistical analysis was performed using one‐way ANOVA + Tukey's *post hoc* test (*P < 0.05).Fold‐change in mRNA expression of different inflammatory markers in the lung of mice infected with PAO1 at 24 hpi.

We used a *Pseudomonas aeruginosa* PAO1 infectious model (Fig EV4) as an *in vivo* tool to study the down‐regulatory properties of the IL‐10 variants generated in this work.

To determine the optimal conditions, we performed a dose–response experiment in which the lung bacterial burden and gene expression of inflammatory markers were evaluated at 24 h post‐infection (hpi) and 48‐hpi (Fig EV4). The bacterial load recovered was higher at 24‐hpi than at 48‐hpi, indicative of bacterial clearance over time (Fig EV4A). In addition, the lungs infected with 1 × 10^5^ CFUs showed higher bacterial burden than those infected with 1 × 10^4^ CFUs, at both analysed time points (Fig EV4A). The inflammatory response was strongest at 24‐hpi (Fig EV4B). The lungs infected with 1 × 10^5^ CFUs showed a significant increase in the expression of *tnf‐a*, *kc*, *mip‐1a*, *mcp‐1*, and *il‐1b* as compared to the uninfected (PBS) or the 1 × 10^4^ CFU‐infected groups (*P* < 0.05 in all cases).

**Figure 6:**
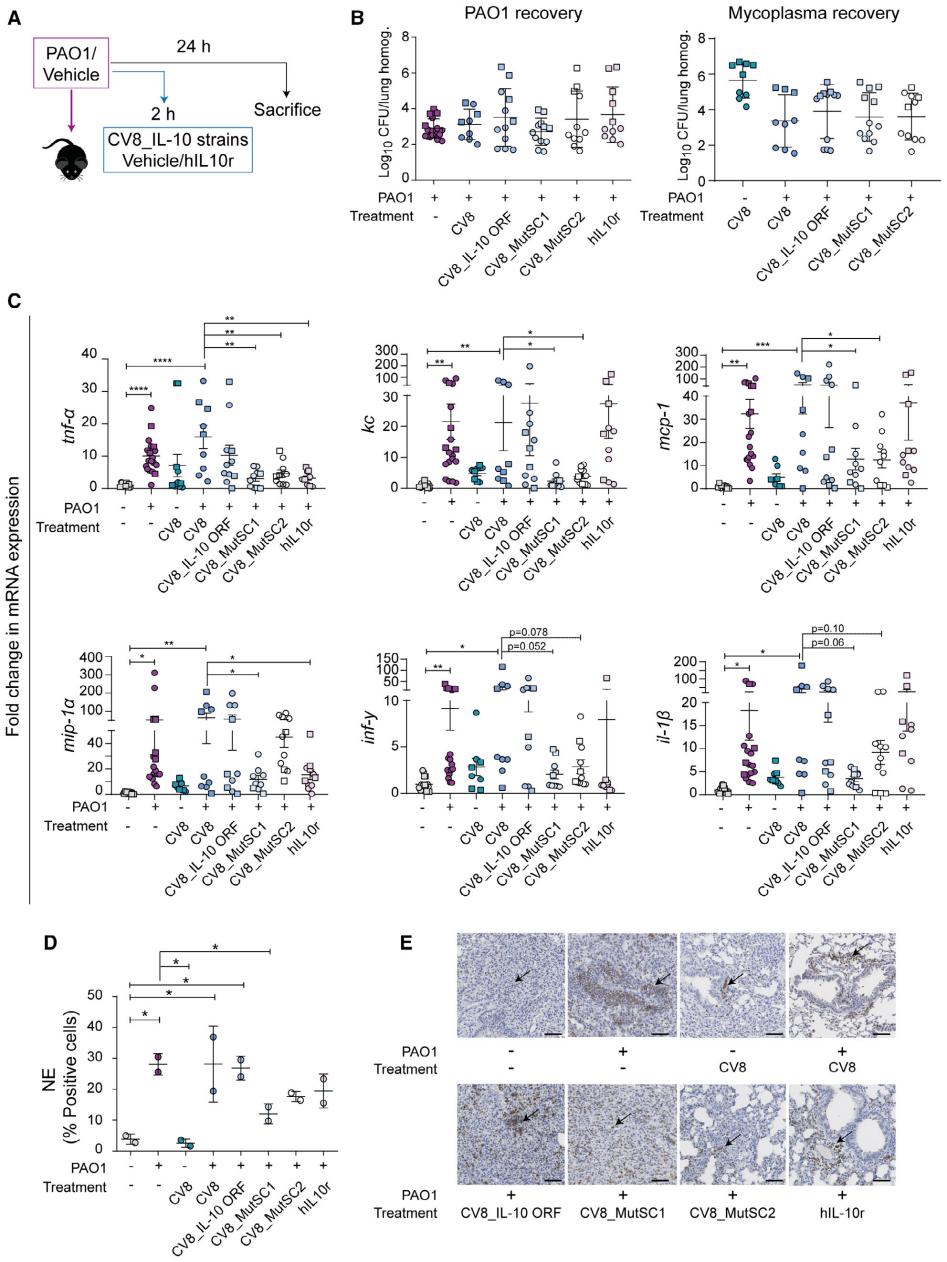
Immunomodulatory effect of IL‐10 variants *in vivo* Each point is a biological replica.
Schematic representation of the experimental design performed.CFUs of PAO1 (left) or *Mycoplasma* strains (right) recovered from lung samples. Data are shown as mean ± SD of Log_10_ CFU/lung homogenate. Two replicates were performed and individual symbols are shown (experiment 1, circle; experiment 2, square).Fold‐change (FC) of mRNA gene expression of inflammatory markers determined by qPCR. The 2^−ΔΔCt^ method was used to normalise the values, using *gapdh* as endogenous control (see Materials and Methods). Data are shown as average ± SD of FC. Statistical analyses were performed using unpaired *t*‐test (*P < 0.05; **P < 0.01; ***P < 0.001; ****P < 0.0001). Two replicates were performed and individual symbols are shown (Experiment 1, circle; Experiment 2, square).Quantitative analysis of immunochemistry (IHC) against neutrophil elastase (NE) of lung samples. Data are represented as the mean ± SD of percentage of positive cells, calculated as follows: % = 100 × (positive cells/positive cells + negative cells; one‐way ANOVA + Tukey's *post hoc* test, *P < 0.05).Representative images of IHC staining of lung samples against NE are shown. Arrows indicate NE‐positive cells. Scale bar size: 50 μm.
Source data are available online for this figure.

To test the anti‐inflammatory properties of the SCs IL‐10 mutants expressed by the CV8 chassis, we infected C57Bl/6 mice first with 1 × 10^5^ CFU of the PAO1 strain, and then, 2 h later, with 1 × 10^7^ CFU of a different *MPN* strain (CV8, CV8_IL‐10 ORF, CV8_MutSC1 or CV8_MutSC2). Control mice received PBS (as a negative control) or 2 μg of hIL‐10r (as a positive control; Fig 6A).

First, we analysed the pulmonary bacterial count of PAO1 and Mycoplasma strains (Fig 6B). No significant differences were observed at 24‐hpi between the groups analysed, ruling out that the differences in the expression of inflammatory genes were biased by the bacterial load present in the lung. We then analysed the inflammatory profile (Fig 6C). The infection with PAO1 resulted in a significant increase in the expression of all markers analysed. The expression of the inflammatory markers in mice after infection with the CV8 strain was comparable to that in the PBS‐treated control mice (Fig 6C), which was additive following the co‐administration of PAO1 + CV8 (Fig 6C). Administration of 2 μg of the hIL10r protein to mice infected with PAO1 (PAO1 + hIL‐10r) significantly reduced the expression of *tnf‐a and mip‐1a* as compared to those infected with PAO1 + CV8 (Fig 6C). In contrast to the results observed for the hIL‐10r‐treated group, the CV8 encoding IL‐10 WT (CV8_IL‐10 ORF) did not significantly modulate the expression of the analysed inflammatory markers (Fig 6C). Inoculation with CV8_MutSC1 significantly reduced the expression of *tnf‐a*, *kc*, *mcp‐1* and *mip‐1a*, with a trend for reduction observed for *inf‐γ* (*P* = 0.052) and *il‐1β* (*P* = 0.06). No differences were observed in the effects on MutSC1 and MutSC2, except for *mip‐1a*, which was better downregulated by CV8_MutSC1 (Fig 6C). The effects of MutSC1 and MutSC2 expressed by the CV8 strain were comparable or even better (except for *mip‐1a* in MutSC2) than the positive control (e.g., administration of 2 μg of hIL‐10r).

Histopathological analysis of the infected lungs showed a cellular infiltrate with predominance of neutrophils (PMN) in all groups infected with PAO1 (Fig 6D and E, and Table EV2), as previously described (Sun *et al*, 2009). We found a significant decrease in the number of PMN in the cellular infiltrate of mice treated with MutSC1, as well as a decrease after treatment with MutSC2 and hIL‐10r, as compared to the PAO1 + PBS group (Fig 6D and E, and Table EV2).

In summary, the combination of the *M. pneumoniae* CV8 chassis with the designed SC IL‐10 variants showed its immunomodulatory potential *in vivo*, improving the effect obtained by administering 2 μg of recombinant IL‐10 intratracheally.

## Discussion

The systemic administration of IL‐10 in humans for the treatment of different pathologies (including inflammatory bowel disease, allergic asthma, and solid tumours) has been tested in different clinical trials but failed in the clinical outcome of these patients due to various problems (Saraiva *et al*, 2020). Limitations of this method include low concentrations of IL‐10 in the targeted tissue and the inability to determine the precise tissue location required for IL‐10 to have the desired anti‐inflammatory effect yet avoid systemic toxicity. The use of live biotherapeutic products (LBP) that incorporate biocontainment strategies and secrete IL‐10 could be an effective strategy for increasing the local concentration of IL‐10 in the targeted tissue, providing a micro, self‐replicating minimal machine as a drug factory. In fact, gene switches to limit *M. pneumoniae* growth for biosafety applications have been developed (Broto *et al*, 2022) and could be implemented in our chassis CV8, potentially without impacting the synthesis capacity of the product. Strategies relying on the local delivery of IL‐10 *in situ* have been explored for some bacteria, such as *L. lactis* or *Bifidobacterium bifidum* in inflammatory bowel disease, with some promising preliminary results in clinical trials (Steidler *et al*, 2000; Mauras *et al*, 2018). However, the delivery of human proteins by bacteria might also be hampered by the foldability of the secreted molecule, the need to make disulphide bridges and/or the need to form a dimer (or a higher oligomer) in order to create a functional protein. Indeed, all of these issues occur in the case of IL‐10, which is a swapped homodimeric protein with two disulphide bridges.

In this work, we engineered human IL‐10 to optimise its expression and efficacy when expressed by a bacterium *in vivo*. The final objective was to decrease possible bacterial pathogenicity by reducing the required bacterial dose to be administered to a patient while retaining the anti‐inflammatory properties of IL‐10. To show the improved properties of our engineered IL‐10 molecules, we selected the lung as the target organ. This choice was made based on reports showing that exogenous delivery of IL‐10 ameliorates different lung diseases (Cypel *et al*, 2009; Shamskhou *et al*, 2019; Lindner *et al*, 2021) and because the lung is a challenging organ due to its reduced microbiome size (Huffnagle *et al*, 2017). Here, we have demonstrated that the human lung bacterium *M. pneumoniae* is able to secrete functional IL‐10 with the two disulphide bridges formed, showing an activity *in vitro* comparable to that of human IL‐10 recombinant protein (hIL‐10r).

Infection of the lung even by non‐pathogenic bacteria could trigger an inflammatory response, so the less bacteria used, the better. As *MPN* has a limited protein synthesis capacity and one way to decrease the dose applied to lungs for therapy is to increase the activity of the secreted molecules. This can be done by either increasing the affinity of IL‐10 for its receptors or improving its stability and foldability (Gorby *et al*, 2020; Minshawi *et al*, 2020). Here we engineered a multiple mutant using FoldX (Delgado *et al*, 2019) and data from the literature (Mut3) as well as SC mutants linking the N‐ and C‐termini (MutSC1) with ModelX (Delgado Blanco *et al*, 2019). While the *in vitro* EC‐50 of these mutants was a significantly better than the WT, it was even better when we combined both into a single mutant (MutSC2). Moreover, MutSC1 and MutSC2 were expressed to levels around 4‐times higher than IL‐10 ORF or Mut3. Combining the higher expression levels and the better EC‐50 shown in the HEK‐Blue™ cell line, the effect *in vitro* of the supernatant of the WT *Mycoplasma* strain expressing MutSC2 is around 60 times higher than that of the IL‐10 ORF strain. This superiority of the MutSC1 and MutSC2 IL‐10 mutants was also observed when testing STAT3 phosphorylation by Western blot or FACS in different cell lines of mouse or human origin. We did not observe significant differences in human or mouse cell lines between these two mutants in activation of STAT3 phosphorylation, indicating that our engineered IL‐10 mutants recognise both human and mouse receptors. In all cases, we found MutSC2 to have a 100% higher relative affinity than MutSC1. In agreement with this, the CV8 strain expressing the IL‐10 MutSC1 or MutSC2 *in vivo* showed a significant down‐regulatory effect on inflammation upon *P. aeruginosa* infection, as compared to CV8‐expressing IL‐10 ORF or to administration of 2 μg of hIL‐10r protein. However, we did not see a significant difference between MutSC1 and MutSC2 *in vivo*. This could be due to the small difference in the EC‐50 between the two mutants and the inherent variability *in vivo*.

In summary, we have generated the first live biotherapeutic chassis for cargo delivery of a designed IL‐10 into the lung tissue that shows a powerful anti‐inflammatory effect in an infectious context. This approach removes the previous constrictions on effective IL‐10 delivery and paves an alternative way for clinical trial success.

## Materials and Methods

### Reagents and Tools table

**Table:** 

Reagent/resource	Reference or source	Identifier or catalogue number
**Experimental models**		
HEK‐BlueTM cell line	InvivoGen	CRL‐1573
Blood samples	Banc de Sang i Teixits, Barcelona, Spain	‐
HAFTL cells (tested for Mycoplasma and authenticated)	Alessandrini *et al* (1987)	‐
BLaER1 cells(tested for Mycoplasma and authenticated)	Gaidt *et al* (2018)	‐
THP‐1 cells (tested for Mycoplasma and authenticated)	ATCC	‐
C57Bl/6 mice	Charles River Laboratories	Strain code: 027
**Bacterial strains**		
*Mycoplasma pneumoniae* M129‐B7	ATCC	29342
*E. coli* NEB® 5‐alpha High‐Efficiency strain	New England Biolabs	C2987H
*Mycoplasma pneumoniae* M129‐B7 WT expressing IL‐10 WT and variants	This work (available for non‐commercial purposes upon request to CRG TBDO office)	‐
*Mycoplasma pneumoniae* M129‐B7 CV8 expressing IL‐10 WT and variants	This work (available for non‐commercial purposes upon request to CRG TBDO office)	‐
*Pseudomonas aeruginosa* PAO1	ATCC	47085
**Recombinant DNA**		
Additional plasmids and more information	This study	Dataset EV5
**Antibodies**		
CD16	BioLegend	3G8
MerTK	BioLegend	590H1 1G1E3
CD163	BioLegend	GH1/61
B7‐H1 PD‐L1	BioLegend	MIH2
CD86	BioLegend	IT2.2
HLA‐DR	BioLegend	I.243
CD16/32 (FcBlock)	BioLegend	93
STAT‐3	Cell Signaling Technology	D1A5,9132
P‐STAT3	Cell Signaling Technology	D3A7
P‐STAT3	Abcam	ab76315
Anti‐Actin	Sigma‐Aldrich	20–33
P‐STAT‐3	Abcam	EP2147Y, Ab76315
Rabbit monoclonal anti‐F4/80 (D2S9R) XP	Cell Signaling	70076S
Rabbit polyclonal Anti‐Neutrophil Elastase	Abcam	Ab68672
Goat anti‐Rabbit IgG (H + L) Cross‐Adsorbed Secondary Antibody, Alexa Fluor 488	Thermo Fisher	Polyclonal A‐11008
Chicken anti‐Rabbit IgG (H + L) Cross‐Adsorbed Secondary Antibody, Alexa Fluor 647 (A‐21443)	Thermo Fisher	Polyclonal A21443
Mouse IgG control	Thermo Fisher	Feb‐02
**Oligonucleotides and sequence‐based reagents**		
PCR primers	This study	Table EV3
**Chemicals, enzymes and other reagents**		
PPLO broth	Difco	255420
FBS	GIBCO	10270‐106
DMEM	Lonza	BE12‐604F
Human recombinant macrophage colony‐stimulating factor (M‐CSF)	PeproTech	300‐25
RPMI	GIBCO	12633012
Cell Dissociation Buffer	Thermo Fisher	13151014
Fc‐Block TruStain FcX	BioLegend	422301
Phenol red solution	Sigma	P3532
Heat‐inactivated horse serum	Life Technologies	26050088
Ampicillin sodium salt	Sigma	#A9518
Streptomycin	GIBCO	15140122
Bacto Agar	Difco	11758223
Trypticase Soy Broth	TSB	T8907
L‐glutamine	GIBCO	25030081
BSA	Sigma Aldrich	A3059
QUANTI‐Blue Solution	InvivoGen	rep‐qbs
Paraformaldehyde	Alfa Aesar	11400580
Glycine	Thermo Fisher Scientific	15527013
10% goat serum	Abcam	ab76315
Formalin	Panreac	252931
**Plastic, columns and other materials**		
Tubes 50 ml	Leucosep®	**227290**
MACS separation columns	Miltenyi, Biotec	130‐042‐302
Glass coverslips	VWR International	C022001
96‐well plates	Nunc Microwell, Thermo Fisher Scientific	#167008
6‐well plates	Thermo Scientific	140675
Protein concentrator MWCO 3 K	Pierce	88526
Pierce™ BCA Protein Assay Kit	Thermo Fisher Scientific	23225
Endoproteinase Lys‐C	Wako	0.129–02541
Trypsin	Promega	V5113
Chymotrypsin	Roche Diagnostics	11418467001
Endo‐GluC	Sigma Aldrich	P6181
C18 column	Nikkyo Technos Co	
TSB agar plates	Fisher Scientific	BD211825
Human IL‐10 recombinant protein	PeproTech	200‐10
RNA extraction RNeasy® Mini Kit	Qiagen	74004
SuperScript II Reverse Transcriptase	Invitrogen	18064022
PCR amplification, SYBR Premix Ex Taq II	Takara	*RR420*
**Software**		
GraphPad Prism 8.0	https://www.graphpad.com	‐
FlowJo 7.6.5	https://www.flowjo.com/solutions/flowjo/downloads	‐
Xcalibur software v2.2	Thermo Fisher Scientific	‐
Proteome Discoverer software suite v2	Thermo Fisher Scientific	‐
PRM data analysis (v21.1.9.353)	*PRM data analysis*	‐
MaxQuant 1.6.10	https://www.maxquant.org/	‐
Prosit	Gessulat *et al* (2019)	‐
iBright CL1500 Imaging System	Thermo Fisher Scientific	‐
NDP.view 2 U123888‐01 software	Hamamatsu	
**Other**		
LSR‐II flow cytometer	BD Biosciences	‐
Gene Pulser Xcell pulser	BIORAD	‐
An LTQ‐Orbitrap Velos Pro mass spectrometer	Thermo Fisher Scientific	‐
HPLC EASY‐nLC 1000	Thermo Fisher Scientific	‐
Orbitrap Eclipse mass spectrometer	Thermo Fisher Scientific	‐
Tissue homogeniser Ultra‐Turrax	IKA	‐
Nanodrop One	Thermo‐Scientific	‐
AriaMx Real‐Time PCR System	Agilent Technologies	‐
Leica TP1020 Tissue Processor	Leica	‐
Leica RM2235 Microtome	Leica	‐
Leica Autostainer XL	Leica	‐
NanoZoomer‐2.0 HT C9600 digital scanner	Hamamatsu	‐

### Methods and Protocols

#### Generation of single‐chain (SC) molecules using ModelX software

SC‐IL10 variants were generated by rewiring the X‐ray IL‐10 dimeric structure (PDBs: 1y6k, 2ilk). The new connectivities were designed using the ModelX tool suite. The ModelX *Bridging* command (cross‐linking mode) was used, which connects a pair of residues selected as anchors with all geometrically compatible peptidic fragments from a custom‐made protein fragment library (PepXDB). PepXDB 120 k structures were extracted from PDB and digested into peptides of different lengths (6–20 aa) and included geometrical descriptors needed for the *Bridging* algorithm. The *Bridging* command allows the user to select different peptide lengths; the output is an ensemble of bridged models with different conformations, sequences, and lengths, whereby linkers/connections with forbidden phi and psi dihedrals in the Ramachandran plot are discarded. Once the sewing patterns are created, an extensive linker screening is performed by running them through the *Bridging* algorithm with exhaustive combinations of anchoring points in an overlapping sliding window around the flanks of the regions to be joined. Every window was queried for different peptide lengths (6–20 aa). The linkers on the bridged models contain the same side chain rotamers that they had on the PDB structure from which they were digested; thus, adaptation to the new SC‐IL10 context is done using the RepairPDB command of FoldX (Delgado *et al*, 2019). RepairPDB identifies residues that have bad torsion angles, Van der Waals' clashes or total energy, and mutates them and their neighbours to themselves, exploring different rotamer combinations to find new energy minima. The resulting models are ranked by global energy (FoldX *Stability* command).

When peptides are longer than numeric positions between the anchors, *Bridging* renumbers surplus residues with res codes that are not recognised by FoldX in further modelling steps. For this reason, SC‐IL10 design required a numerical rearrangement of the monomeric protein residues in the dimer, allowing numeric 18‐aa–long gaps between the regions to be connected. This is very important for the extreme case in which anchoring residues flank the numeric gap. To cover that situation, renumbering was done to create gaps of 18 numeric positions around the regions to be connected, which was enough to accept length fragments ranging from 6 to 20 residues without using the residue codes. The difference between 18 and 20 is that anchoring points are required for the algorithm to position the bridges, but they will be substituted with the terminal residues of the peptides found. *Bridging* also replaces all fringe residues between the anchors with the ones from the peptide. For clarity, the templates generated before using *Bridging* are termed “sewing patterns” (Fig 3). As the numeric gap is not a spatial gap, *Bridging* can also accept smaller peptide fragments, creating a discontinuous numbering.

MutSC1 was generated in several steps: residues 12′–17′ (note that monomer 2 residues and atoms are depicted by’) were deleted, as they were pointing away from the C′‐terminal and were not in contact with R1, and residues 18′–156′ were renumbered as 169–307 and were joined into one molecule, creating sewing pattern 1. As the best anchoring points, the residues 147 (Ile) and 169 (*residue 18′ in monomer 2*, *Asn*) were selected and then connected using a 17‐aa fragment from PDB 2wyu (residues 73–89). The original sequence in 2wyu of the chosen bridge segment was LDALFAGVKEA‐FGGLD‐Y. Residues LDALFAGVKEA and the last Y structurally superimposed with IL‐10. Therefore, all residues were mutated back to the original IL‐10 sequence unless the newly‐added residues improved protein stability (Asn169 to Tyr).

#### Plasmid generation

All plasmids generated in this work were assembled following the Gibson method (Gibson *et al*, 2009). When required, Integrated DNA Technologies (IDT) Corporation performed gene synthesis (gBlock double‐stranded fragments) and oligonucleotides synthesis. Gene amplification was carried out with Phusion DNA polymerase (Thermo Fisher Scientific). The promoter sequence was unified for all IL‐10 variants to P3 synthetic promoter (Yus *et al*, 2017), and to the secretion signal for *M. pneumoniae* termed s142 (patent 15/553552 USA). A detailed list of plasmids, strains and their genetic parts is available (Dataset EV5). All plasmids were verified by PCR and Sanger sequencing (GATC biotech).

#### Bacterial strains and culture conditions

The WT *M. pneumoniae* M129‐B7 strain (ATCC 29342) and all the *M. pneumoniae* strains generated in this work (CV8; see below) are described in Dataset EV5. Mycoplasma strains were grown at 37°C under 5% CO_2_ in tissue culture flasks (Corning) with Hayflick liquid medium. Hayflick was prepared by mixing 800 ml of non‐complete medium A (20 g PPLO broth [Difco], 30 g HEPES [100 mM final], 25 ml 0.5% phenol red solution [Sigma]), 200 ml heat‐inactivated horse serum (Life Technologies), 20 ml sterile‐filtered 50% glucose, and 1 ml of a 100 mg/ml stock of ampicillin (final concentration 100 μg/ml, ampicillin sodium salt [Sigma]). If growth on the plate was required, Hayflick broth was supplemented with 0.8% Bacto agar (Difco). Hayflick broth was supplemented with chloramphenicol (Cm; 20 μg/ml) for cell selection, as needed. *Pseudomonas aeruginosa* PAO1 was grown in Trypticase Soy Broth (TSB) agar plates at 37°C. The *E. coli* NEB® 5‐alpha High‐Efficiency strain (New  England Biolabs) was used for cloning and was grown at 37°C in LB broth or on LB agar plates supplemented with ampicillin (100 μg/ml).

#### Blood monocyte assays

##### Blood monocyte isolation

Blood samples from four healthy donors were provided by the Banc de Sang i Teixits (Barcelona, Spain), under agreement no. 160002 approved by the Spanish Ministry of Science and Technology. Written informed consents were obtained from the donors before sample collection. Peripheral blood mononuclear cells (PMBCs) were isolated from the buffy coat using Leucosep® tubes, according to the manufacturer's instructions. Briefly, 10 ml of blood samples was diluted with a ratio of 1:5 with phosphate‐buffered saline solution (PBS), poured into the Leucosep® tubes and centrifuged for 15 min at 800 × *g* at room temperature. PMBCs were collected using a pasteur pipette, washed twice with PBS, and finally resuspended with 3 ml PBS. Monocytes were isolated by CD14^+^ magnetic labelling and differentiated into macrophages as previously described (Troegeler *et al*, 2014). Briefly, cells were purified using CD14 microbead‐positive selection and MACS separation columns (Miltenyi Biotec), according to manufacturer's instructions. To differentiate monocyte‐derived macrophages, monocytes were adhered to glass coverslips (VWR International) in 6‐well plates (Thermo Scientific), at 1.5 × 10^6^ cells/well for 1 h at 37°C in warm RPMI 1640 medium (GIBCO). Medium was then supplemented to a final concentration of 10% foetal bovine serum (FBS, Sigma‐Aldrich) and human recombinant macrophage colony‐stimulating factor (M‐CSF, PeproTech) at 20 ng/ml. Cells were allowed to differentiate for 6–8 days.

##### IL‐10 stimulation and analysis by flow cytometry

To determine functionality of the IL‐10 produced by Mycoplasma, conditioned monocyte‐macrophages were stimulated and stained as previously described (Troegeler *et al*, 2014). The final volume for each culture was 2.5 ml/well in RPMI (supplemented with 10% FBS). The inductions were performed for 24 h with supernatants of *M. pneumoniae* expressing IL‐10 ORF (20 ng/ml), supernatants of *M. pneumoniae* WT or human IL‐10 recombinant protein (hIL‐10r; 20 ng/ml) or Hayflick (negative control). In all cases, the added volume was 20% of the final volume. Cells were then harvested using the Cell Dissociation Buffer (Life Technologies), centrifuged for 5 min at 300 × *g* at 4°C, blocked for 20 min in cold FACS buffer (PBS pH 7.2, 1% FBS) with Fc‐Block TruStain FcX solution (BioLegend) and stained for 30 min with fluorophore‐conjugated antibodies. After staining, cells were washed with a cold FACS buffer, centrifuged for 5 min at 300 × *g* at 4°C and analysed by flow cytometry using LSR‐II flow cytometer (BD Biosciences). Control cells were incubated with the corresponding isotype control antibody using a general dilution of 1:200 and processed in parallel. Data were acquired and analysed using the FlowJo 7.6.5 software. The results are expressed as an average of fold‐change (FC) of the mean fluorescence intensity (FC MFI), resulting from normalising the data with the Hayflick group (FC ~ 1).

#### Eukaryotic cell culture

HAFTL cells are a fetal liver‐derived, Ha‐ras‐oncogene transformed mouse pre‐B cell line (Alessandrini *et al*, 1987). BLaER1 are human B‐cell precursor leukaemia cell lines (Gaidt *et al*, 2018). Both cell lines were kindly provided by Professor Thomas Graf. The THP‐1 cell line is a monocyte cell line derived from peripheral blood purchased from ATCC. Cells were grown in RPMI (12633012, GIBCO), supplemented with 2 mM L‐glutamine (25030081, GIBCO), 100 U/ml penicillin + 100 ng/ml streptomycin (15140122, GIBCO), and 20% FBS (10270‐106, GIBCO).

The HEK‐Blue™ cell line carrying a SEAP reporter construct was purchased from InvivoGen (InvivoGen, San Diego, CA, USA) in July 2020. Cells were grown in DMEM (Lonza, BE12‐604F) supplemented with 10% FBS, 2 mM L‐glutamine and 100 μg/ml normocin and selection antibiotics. Cells were passed when 70% confluence was reached, following the manufacturer's recommendation.

#### Generation of the *M. pneumoniae* CV8 strain

CV8 is a derivative strain of the attenuated CV2 strain (Δ*mpn133*, Δ*mpn372*; 18) in which *mpn051* has been replaced by the *gpsA* gene from *Mycoplasma penetrans*. The enzymes encoded by these two genes are involved in the oxidation of glycerol‐3‐phosphate, whereby the GpsA enzyme generates the metabolic by‐products of NADH, and the GlpD enzyme, H_2_O_2_ (note that this H_2_O_2_ production is crucial for the cytotoxic effects of *M. pneumoniae*). The CV8 strain also differs from CV2 in that a puromycin‐resistance gene was removed from its location in the genome (*mpn560*). Mutants were generated using genome editing tools adapted to mycoplasma (Piñero‐Lambea *et al*, 2020) and long stretches of ssDNA were used, produced as previously described (Burgos *et al*, 2020). See Dataset EV5 and Table EV3 for the sequences of the primers and the plasmids used. Transposon vectors were generated by fusing the synthetic promoter P3 to all IL‐10 variants, which contained the signal peptide 142 (s142) at their N‐termini for secretion.

To generate the *M. pneumoniae* expression cells, the different plasmids were transformed into either WT or CV8 *M. pneumoniae*. Cells were grown in a 75‐cm^2^ tissue flask (Corning) containing 20 ml fresh Hayflick and incubated at 37°C under 5% CO_2_ until the late exponential phase. Cells were washed twice with a pre‐cooled electroporation buffer (272 mM sucrose, 8 mM HEPES, pH 7.4), resuspended, scraped off and passed 10‐times through a 25‐gauge (G25) syringe needle. Cell aliquots of 50 μl in 0.1‐cm cuvettes with 2 μg of the desired plasmid were kept on ice for 20 min. The electroporation settings were set in 1,250 V/25 μF/100 Ω in a BIO‐RAD Gene Pulser Xcell apparatus. After the pulse, 420 μl fresh Hayflick was added to the cells. From this culture, 80 μl were inoculated in a 25‐cm^2^ tissue flask (Corning) with 5 ml fresh Hayflick with the selection antibiotic (i.e., 20 μg/ml chloramphenicol). From the different *M. pneumoniae* expression cells, 50‐μl stock inoculum was added to a 25‐cm^2^ tissue flask (Corning) containing 5 ml fresh Hayflick, and this was grown for 48 h. Culture media were then collected and centrifuged at 6,720 *g* for 10 min, and the supernatants were stored at −80°C in 1 ml aliquots.

#### Disulphide identification by mass spectrometry

From the aliquots initially prepared and stored at −80°C, an initial inoculum of 25 μl of hIL‐10, MutSC1 or MutSC2 was inoculated in a T25‐cm^2^ flask with 5 ml Hayflick medium supplemented with Cm in duplicate. After 24 h, the medium was discarded from one of the duplicates and replaced with Version 13 of the defined medium (see patent application EP20382261). After 36 h, the supernatant of both replicates was collected and processed for proteomic analysis. No differences were observed in protein abundance for either condition. For further characterisation, samples were grown in Version 13 of the defined medium.

Supernatants were collected, concentrated with MWCO 3 K and centrifuged for 15 min at 9,660 *g* with 700 μl supernatant. Then, 200 μl were added to the same MWCO column, and the mixture was centrifuged for 60 min at 4°C at 9,660 *g*. The final concentrated volume of approximately 100 μl was split into two: one for BCA quantification and the other for MS analysis. Each of the freshly concentrated aliquots was resuspended in 100 μl of SDS 4%, 0.1 M HEPES. Supernatant lysates were quantified by BCA using the Pierce™ BCA Protein Assay Kit (Thermo Fisher Scientific, 23225) and sent to the CRG/UPF MS Facility for analysis.

For sample preparation, the cysteines were labelled by alkylating with N‐ethylmaleimide (30 nmol, 37°C, 60 min), and samples were then precipitated with six volumes of cold acetone. Pellets were dissolved in 6 M urea/200 mM ammonium bicarbonate, and samples were reduced with dithiothreitol (30 nmol, 37°C, 60 min) and alkylated in the dark with iodoacetamide (60 nmol, 25°C, 30 min). Samples were then digested using 200 mM ammonium bicarbonate. For hIL‐10, and MutSC2, the resulting protein extract was first diluted to 2 M urea and then incubated with the endoproteinase Lys‐C (endo‐LysC; 1:100 w:w, 37°C, 6 h, Wako); samples were then diluted 2‐fold (1:100 w:w) and digested with (i) trypsin (37°C, overnight; Promega); (ii) chymotrypsin (25°C, overnight; Roche Diagnostics); or (iii) GluC (25°C, overnight; Sigma Aldrich). In addition, the combinatorial digestion mix for MutSC1 was the following: (i) endo‐LysC plus trypsin: diluted to 2 M urea for endo‐LysC (1:100 w:w, 37°C, 6 h) and then diluted 2‐fold for trypsin (1:100 w:w, 37°C, overnight); (ii) endo‐LysC plus chymotrypsin: diluted to 2 M urea for endo‐LysC (1:100 w:w, 37°C, 6 h) and then diluted 2‐fold for trypsin digestion (1:100 w:w, 25°C, overnight); (iii) LysC plus GluC: diluted to 2 M urea for endo‐LysC (1:100 w:w, 37°C, 6 h), and then diluted 2‐fold for GluC (1:100 w:w, 25°C, overnight); and (iv) trypsin plus GluC: diluted to 6 M urea for trypsin digestion (1:100 w:w, 37°C, overnight) and then digested with GluC (1:100 w:w, 25°C, overnight). In all cases, the peptide samples after digestion were acidified with formic acid and desalted over a MicroSpin C18 column (The Nest Group, Inc) prior to LC–MS/MS analysis.

For chromatographic and MS analysis, the following protocol was used: samples were analysed using an LTQ‐Orbitrap Velos Pro mass spectrometer (Thermo Fisher Scientific) coupled to an EASY‐nLC 1000 (Thermo Fisher Scientific). Peptides were loaded onto the 2‐cm Nano Trap column with an inner diameter of 100 μm packed with C^18^ particles of 5‐μm particle size (Thermo Fisher Scientific) and were separated by reversed‐phase chromatography using a 25‐cm column with an inner diameter of 75 μm, packed with 1.9 μm C18 particles (Nikkyo Technos Co). Chromatographic gradients started at 93% buffer A and 7% buffer B with a flow rate of 250 nl/min for 5 min, and gradually increased to 65% buffer A and 35% buffer B at 120 min. After each analysis, the column was washed for 15 min with 10% buffer A and 90% buffer B. Buffer A, 0.1% formic acid in water; buffer B, 0.1% formic acid in acetonitrile. The mass spectrometer was operated in positive ionisation mode with nano spray voltage set at 2.1 kV and source temperature at 300°C. Ultramark 1621 was used for external calibration of the FT mass analyser prior to analyses, and an internal calibration was performed using the background polysiloxane ion signal at m/z 445.1200. Measurements were acquired in the data‐dependent acquisition (DDA) mode, and full MS scans with 1 micro scan at a resolution of 60,000 were used over a mass range of m/z 350–2,000 with detection in the Orbitrap. Auto gain control (AGC) was set to 1E6, dynamic exclusion (60 s), and a charge state filtering disqualifying singly charged peptides was activated. In each cycle of DDA analysis, and following each survey scan, the top 20 most intense ions with multiple charged ions above a threshold ion count of 5,000 were selected for fragmentation. Fragment ion spectra were produced via collision‐induced dissociation (CID) at a normalised collision energy of 35% and were acquired in the ion trap mass analyser. AGC was set to 1E4, an isolation window of 2.0 m/z, an activation time of 10 ms and a maximum injection time of 100 ms were used. All data were acquired with Xcalibur software v2.2. Digested bovine serum albumin (New England Biolabs, cat. #P8108S) was analysed between each sample to avoid sample carryover and to assure stability of the instrument; QCloud (Chiva *et al*, 2018) was used to control instrument longitudinal performance during the project.

Data were analysed using the Proteome Discoverer software suite (v2.0, Thermo Fisher Scientific) and the Mascot search engine (v2.6, Matrix Science; Perkins *et al*, 1999). Data were searched against a *M. pneumoniae* (87,071 entries) plus hIL‐10, MutSC1, MutSC2 and a list of common contaminants (Beer *et al*, 2017) and all of the corresponding decoy entries. For peptide identification, a precursor ion mass tolerance of 7 ppm was used for MS1 level, trypsin was chosen as the cleavage enzyme and up to three missed cleavages were allowed. The fragment ion mass tolerance was set to 0.5 Da for MS2 spectra. Oxidation of methionine, N‐terminal protein acetylation, carbamidomethylation of cysteines and N‐ethylmaleimide were used as variable modifications. The false discovery rate (FDR) in peptide identification was set to a maximum of 5%. Peptide quantification data were retrieved from the “Precursor ion area detector” node from Proteome Discoverer (v2.0) using 2 ppm mass tolerance for the peptide extracted ion current (XIC). The obtained values were used to calculate the protein's top three areas with the unique peptide for protein ungrouped.

For MutSC1, and given the fact that carbamidomethylated cys12 was not clearly identified in the first MS analysis, an additional step of PRM (parallel reaction monitoring)‐chromatographic and MS analysis was performed. To confirm that the first cysteine is carbamidomethylated, the sample was digested with endo‐LysC plus trypsin were analysed using an Orbitrap Eclipse (Thermo Fisher Scientific) coupled to an EASY‐nanoLC 1200 UPLC system (Thermo Fisher Scientific) with a PRM method. The peptides were loaded directly onto the analytical column and were separated by reversed‐phase chromatography using a 50‐cm column with an inner diameter of 75 μm, packed with 2 μm C^18^ particles spectrometer (Thermo Scientific, San Jose, CA, USA). Chromatographic gradients started at 95% buffer A and 5% buffer B with a flow rate of 300 nl/min for 5 min and gradually increased to 25% buffer B and 75% A over 79 min and then to 40% buffer B and 60% A over 11 min. After each analysis, the column was washed for 10 min with 10% buffer A (0.1% formic acid in water) and 90% buffer B (0.1% formic acid in 80% acetonitrile). The mass spectrometer was operated in positive ionisation mode with an EASY‐Spray nanosource at 2.4 kV and at a source temperature of 305°C. A full MS scan with 1 micro scan at resolution of 30,000 was used over a mass range of m/z 350–1,400, with detection in the Orbitrap mass analyser. A PRM method was used for data acquisition with a quadrupole isolation window set to 1.4 m/z and MSMS scans over a mass range of m/z 300–2,000, with detection in the Orbitrap at resolution of 60,000. MSMS fragmentation was performed using HCD at 30 NCE, the auto gain control (AGC) was set to 1 × 10^5^ and maximum injection time of 502 ms. Peptide masses (m/z) were defined in the mass list and are shown in Dataset EV1. For PRM data analysis, the Skyline‐Daily software (v21.1.9.353) was used to generate the libraries, which were observed as the output of the MaxQuant (1.6.10) search and predicted with Prosit (Gessulat *et al*, 2019), and to extract the fragment areas of each peptide. Data are included in Dataset EV1.

#### Characterisation of IL‐10‐expressing CV8 and WT *M. pneumoniae* cells

For the IL‐10‐expressing WT and CV8 cells, 50 μl of stock inoculum was added to a 25‐cm^2^ tissue flask (Corning) containing 5 ml fresh Hayflick and grown for 48 h. Culture media were then collected and centrifuged at 6,720 *g*, 10 min, and the supernatant was stored at −80°C for further quantification. Cells were collected by scraping in 1 ml PBS and centrifuged for 5 min at 6,720 *g* at 4°C; pellets were resuspended in 1 ml PBS and centrifuged for 5 min at 6,720 *g* at 4°C, and supernatants were discarded. Finally, pellets were disaggregated in 200 μl of lysis buffer (4% SDS, 0.1 M HEPES). Protein lysates were quantified using the Pierce BCA Protein Assay kit (Thermo Fisher) following the manufacturer's protocol. In parallel, the levels of the IL‐10 variants in the supernatants were quantified by ELISA as previously described. The secretion capacity of IL‐10 (μg/ml) was normalised to biomass (protein content of the total culture in μg/ml) for each of the strains. Three biological replicates were used.

#### Testing the activity of IL‐10 candidates in eukaryotic cells

##### Western blotting

For Western blotting with primary macrophages, total protein lysates were extracted as previously described (Troegeler *et al*, 2014). After protein transfer, membranes were incubated overnight at 4°C with rabbit monoclonal anti‐Signal transducer and activator of transcription STAT3 and anti‐phosphoY705‐STAT3 or with a monoclonal anti‐actin. For a time‐course experiment with HAFTL cells, cells were seeded at 1 × 10^6^ cells/well in a 6‐well plate (Corning). The final volume for each culture was 2.5 ml/well in RPMI (supplemented with 10% FBS and penicillin/streptomycin). The inductions were performed for 20 min with supernatants of *M. pneumoniae* expressing IL‐10 ORF at 20 ng/ml. Cells in a non‐induced (NI) condition were incubated with supernatant of WT *M. pneumoniae*. Cell mixtures were then centrifuged at 6,720 *g* for 10 min, the supernatant was discarded, and cells were washed with 1 ml of pre‐cooled PBS and then centrifuged with the same settings. Protein lysates were then extracted as indicated above. As a blocking agent, BSA 5% (Sigma Aldrich) was used. After protein transfer, samples were incubated with primary anti‐pSTAT3 antibody (Abcam) or anti‐STAT3 antibody ( Cell Signaling). For the secondary antibody, anti‐rabbit IgG (peroxidase antibody produced in goats; A9169, Sigma‐Aldrich) was used. Proteins were detected using the SuperSignal West Femto Maximum Sensitivity Substrate kit and visualised with iBright CL1500 Imaging System.

The same protocol was used for the experiment of IL‐10 ORF, MutSC1 and MutSC2 in two cellular backgrounds: HAFTL and THP‐1, whereby the induction time was set to 20 min, and the IL‐10 concentration 20 ng/ml.

##### Colorimetric analysis in HEK‐Blue™ cells

After supernatant quantification by ELISA, 200‐μl aliquots of the different supernatants of *M. pneumoniae* cells were stored at −80°C. Using fresh Hayflick medium for diluting the samples, different 500 μl “candidate aliquots” of the following concentrations (30, 15, 7.5, 3.75, 1.88, 0.94, 0.47, 0.23, 0.094, 0.05 or 0.02 ng/ml) were prepared for each mutant candidate and as well for the recombinant protein. The volume of supernatant needed was determined by ELISA quantification, and volumes were adjusted accordingly with a fresh Hayflick medium to reach the concentration stated above in the 20 μl supplement. In parallel, a HEK‐Blue™ IL‐10 cell suspension was prepared at 280,000 cells/ml in pre‐warmed DMEM supplemented with 10% FBS, 2 mM L‐glutamine (without antibiotics). Thereafter, 180 μl of cells per well were seeded in a 96‐well plate (Nunc Microwell, Thermo Fisher Scientific, #167008). From this, 20 μl of each “candidate aliquot” or the recombinant protein at a fixed concentration prepared in Hayflick medium was added, and cells were kept 24 h at 37°C and 5% CO_2_ (induced HEK‐Blue™). After 24 h, 180 μl of QUANTI‐Blue Solution (Alkaline phosphatase detection medium, #repqbs, InvivoGen) was mixed with 20‐μl induced HEK‐Blue™ cells in a new 96‐well plate. Cells were then incubated 60 min at 37°C, and absorbance (630 nm) was measured in the spectrophotometer Tecan i‐control, 2.0.10.0.

##### Flow cytometry assays of cell lines

To determine the relative stimulation of STAT3 phosphorylation by the different SC‐IL10 mutants in BlaER1 and HAFTL cells, cells were plated in 96‐well plates and stimulated with the corresponding *M. pneumoniae* supernatants for 20 min at 37°C. For HAFTL cells, the concentration range was between 0.19 and 12 nM; for BLaER1 cells, the concentration range was between 0.03 and 12 nM. This was followed by fixation with paraformaldehyde (Alfa Aesar) for 10 min at room temperature. Cells were then permeabilised with PBS^+^ Twin (PBST 0.1%), and the reaction was arrested by incubation with 0.3 M glycine (Thermo Fisher Scientific) plus 10% goat serum (ab7481, Abcam) in PBS for 20 min. Cells were incubated with primary antibody anti‐pSTAT3 (ab76315, Abcam) at a 1/400 dilution for 20 min at room temperature and then washed with PBS. For HAFTL cells, a secondary fluorescent antibody (anti‐IgG rabbit‐Alexa Fluor 488; Thermo Fisher Scientific) was added, and samples were incubated for 20 min at room temperature and then washed twice with PBS. For BlaER1 cells, only populations without constitutively‐expressed GFP were analysed, to avoid interference of GFP with the fluorescence of the secondary antibody. In this case, the secondary antibodies used were anti‐IgG rabbit‐Alexa Fluor 488 for replicates one, two and three, and chicken anti‐rabbit IgG Alexa Fluor 647 Thermo Fisher Scientific) for replicate four. Samples were analysed in a BD LSR II Flow Cytometer. For HAFTL cells, three biological replicates with two technical replicates each were used; for BLaER1, four biological replicates with two technical replicates each were used. For the analysis, the background was subtracted from the mean fluorescence intensity (MFI). Samples were normalised to the maximal WT IL‐10 ORF value of each of the replicates (maximal IL‐10 ORF signal set to 1). The results were plotted in Prism 8 (GraphPad). To estimate the EC‐50, IL‐10 concentrations were selected with no saturation, in which only the specific binding was measured (HAFTL: 1.5 × 10^9^ M to 1.875 × 10^10^ M; BLaER1: 1.87 × 10^10^ to 2.93 × 10^12^). The curve was fitted to a Specific binding with Hill slope in Prism 8 (GraphPad), whereby Bmax (saturation signal) was set for each experiment accordingly.

#### Inferring EC‐50 data

The *Mycoplasma* cell culture supernatants were diluted in Hayflick medium to adjust the IL‐10 concentration, covering a range from 0.0243 to 60 ng/ml that encompassed the dynamic range described for HEK‐Blue™ (see Materials and Methods section). To calculate the molar concentration for each IL‐10 variant, it was assumed that all IL‐10 variants (including IL‐10 ORF) have a molecular weight of 36 KDa, with the exception of MutM, which has a molecular weight of 18 KDa. The EC‐50 was calculated in molar (M).

The changes in absorbance at 630 nm due to different IL‐10 tested concentrations were fitted to a saturation binding model using the following equation:

$$Y=B\;{\mathrm{Max}}^{*}\mathrm{Xh}/\left(\mathrm{EC}-50\;h+\mathrm{Xh}\right);$$

In this equation, EC‐50 is the apparent dissociation constant (as the number of active receptors per cell is unknown), h is the Hill slope, and B Max is the saturation signal. This equation assumes specific binding only; all non‐specific signals were subtracted.

At least three biological replicates (and up to six biological replicates), with two technical replicates for each condition, were used. In the conditions in which saturation was not reached, Bmax was fixed to estimate the EC‐50 numbers. The different experiments were analysed independently and fitted using GraphPad Prism 9 software.

#### Mice experiments

C57Bl/6 female and male mice (18–20 g), aged 6–8 weeks, were purchased from Charles River Laboratories (France), randomly divided into groups and housed under pathogen‐free conditions at the PRBB animal facility (registration number B9900073). Animal handling and procedures followed the current European (Directive 86/609/EEC) and National (Real Decreto 53/2013) legislations as well as the FELASA and ARRIVE guidelines and obtained the approval of the Animal Experimentation Ethic Committee of Barcelona Biomedical Research Park (PRBB) and the local Government authorisation.

##### *M. pneumoniae* lung infection in mice

A bacterial suspension of 100 μl (of WT or CV8) in PBS containing ~0.5 to 1 × 10^8^ CFU/ml (~0.5 to 1 × 10^7^ CFU/mouse) was used for intratracheal (IT) infection in mice previously anaesthetised with isoflurane 2% (ISOFLO, Covegan). At 2 or 4 days post‐infection (dpi), animals were sacrificed by cervical dislocation, and lungs were removed and processed. The left lung was weighed and homogenised 1:10 (wt/vol) in PBS in sterile individual bags (Stomacher80, Seward Medical) for bacterial load quantification as described previously (Grilló *et al*, 2012; detection limit = Log_10_ 0.52 to 0.9 CFU/g lung tissue). The right lung was (i) insufflated with 50 μl of 10% methanol‐stabilised formalin (Panreac) for histopathological analyses (postcaval lobe); or (ii) frozen in N_2_ and stored at −80°C until use, for RNA extraction (inferior lobe). Control animals were inoculated with 100 μl of the vehicle solution and processed in parallel. Infections were performed in groups of at least 3 mice per strain (*n* ≥ 3).

##### Acute lung infection of *P. aeruginosa* PAO1 in the C57Bl/6 mouse model

The *P. aeruginosa* PAO1 strain was grown at 37°C, 5% CO_2_, for 14–16 h in TSB agar plates at 37°C (#BD211825, Fisher Scientific). Bacteria were collected in PBS, and suspensions were normalised to optical density 600 nm (OD_600_) = 0.5. For dose–response assay, 100 μl containing ~1 × 10^5^ or 1 × 10^6^ CFU/ml (~1 × 10^4^ or ~1 × 10^5^ CFU/mouse, respectively) was used for IT infection. At 24 or 48 h post‐inoculum (hpi), lungs were processed following the method described above. Control animals were inoculated with 100 μl of the vehicle solution (PBS) and processed in parallel. Infections were performed in groups of at least 5 mice per strain (*n* ≥ 5).

##### Immunomodulatory effect of CV8 expressing hIL‐10 *in vivo*

To evaluate the immunomodulatory effect of the Mycoplasma strains encoding hIL‐10, C57Bl/6 mice were infected IT with 50 μl of *P. aeruginosa* PAO1 bacterial solution containing ~2 × 10^6^ CFU/ml (~1 × 10^5^ CFU/mouse); after 2 h, mice were infected IT with 50 μl of the *Mycoplasma* strains containing ~2 × 10^8^ CFU/ml (~1 × 10^7^ CFU/mouse). Control animals were processed in parallel and treated with (i) 2 μg of human IL‐10 recombinant protein (hIL‐10r) (PeproTech), or (ii) sterile PBS (vehicle solution). At 24 hpi with PAO1, animals were sacrificed, and lungs were aseptically removed for the experiments described above. Two biological replicates were performed.

##### RNA extraction and qPCR of inflammatory parameters

Right lungs that had been stored at −80°C were homogenised using Ultra‐Turrax (IKA), and total RNA was isolated using RNeasy® Mini Kit (Qiagen) following the manufacturer's instructions. RNA concentrations were measured spectrophotometrically using Nanodrop One (Thermo‐Scientific), and sample RNA integrity was confirmed by 1% agarose gel electrophoresis. RNA samples with ratio absorbance measurements at 260 nm/280 nm of 1.8–2.1 were used. Complementary DNA (cDNA) from whole‐lung cells was synthesised from total RNA (1 μg) using SuperScript II Reverse Transcriptase reagents (Invitrogen). PCR amplification was performed using SYBR Premix Ex Taq II (Tli RNaseH Plus; Takara), and fluorescence was analysed with AriaMx Real‐Time PCR System (Agilent Technologies). Primer pairs for *gapdH*, *tnf‐a*, *kc*, *mip‐1a*, *mcp‐1*, *inf‐y* and *il‐1b* detection are shown (Table EV3). The 2^−ΔΔCt^ method was used to determine the relative abundance of mRNA in each experimental condition, using *gapdH* as an endogenous control and normalising to the values obtained in the PBS group as follows: ΔCt = mean Ct analysed gene − mean Ct *gapdH*; ΔΔCt = ΔCt treated group/ΔCt PBS group; fold‐change in mRNA expression = 2^−ΔΔCt^.

##### Histological and immunohistochemistry (IHC) analysis of lung samples

After necropsy, the postcaval lobe was fixed in formalin (Panreac), trimmed and automatically processed in ethanol series and xylene (Leica TP1020 Tissue Processor). Thereafter, tissues were embedded in paraffin (Tec2900 Histo‐Line Workstation), sectioned to 4 μm (Leica RM2235 Microtome) and stained by haematoxylin and eosin (H&E) following standard procedures (Leica Autostainer XL). Preparations were manually mounted onto glass slides and examined by light microscopy. Tissue was double‐blinded and evaluated in the Histopathology Facility of the Institute for Research in Biomedicine‐IRB (Barcelona). Alveolar cell infiltrate and perivascular‐peribronchial/solar infiltrate were used as histopathological parameters, and a quantitative score was established (0–5: 0, none; 1, minimal; 2, mild; 3, moderate; 4, intense; 5 very intense); the final score of each sample was obtained by the sum of both parameters. For IHC, paraffin‐embedded tissue sections were stained with the rabbit monoclonal anti‐F4/80 (D2S9R) XP (#70076 S, Cell Signaling) as a marker of macrophages (Kamei *et al*, 2016), or the rabbit polyclonal Anti‐Neutrophil Elastase (#Ab68672, Abcam) as a marker of neutrophils (Hirche *et al*, 2008) using the Leica Bond RX. Specificity of staining was confirmed by staining with a rabbit IgG isotype control (#Ab27478, Abcam). Brightfield images were acquired with a NanoZoomer‐2.0 HT C9600 digital scanner (Hamamatsu) equipped with a 20× objective. All images were visualised with the NDP.view 2 U123888‐01 software (Hamamatsu, Photonics, France), using a gamma correction set at 1.8 in the image control panel. Output results obtained from image analyses were represented as % positive cells [% = 100 × (positive cells/positive cells + negative cells)].

#### Statistical analyses

In all cases, statistical significance was set to *P* < 0.05. Analyses were performed using Prism software (GraphPad Software) statistical package and are detailed in each figure legend.

## Author contributions

**Ariadna Montero‐Blay:** Conceptualization; validation; writing – original draft. **Javier Delgado Blanco:** Software; methodology. **Irene Rodriguez‐Arce:** Validation; methodology; writing – review and editing. **Claire Lastrucci:** Methodology. **Carlos Piñero‐Lambea:** Resources. **Maria Lluch‐Senar:** Resources; writing – review and editing. **Luis Serrano:** Conceptualization; supervision; writing – original draft.

## Disclosure and competing interests statement

The work described here was done before the creation of the start‐up Pulmobiotics, a company interested in the development of *M. pneumoniae* as a vector to treat human lung diseases. In any case, we would like to state that Carlos Piñero and Maria Lluch are now working in the Pulmobiotics company and that Luis Serrano and Maria Lluch are co‐founders of the company. Luis Serrano is an editorial advisory board member. This has no bearing on the editorial consideration of this article for publication.

## Supporting information



Expanded View Figures PDF

Table EV1

Table EV2

Table EV3

Dataset EV1

Dataset EV2

Dataset EV3

Dataset EV4

Dataset EV5

Source Data for Figure 1

Source Data for Figure 4

Source Data for Figure 5

Source Data for Figure 6

## Data Availability

The authors confirm that the data supporting the findings of this study are available within the article and its supplementary materials.

## References

[msb202211037-bib-0001] Alessandrini A , Pierce JH , Baltimore D , Desiderio SV (1987) Continuing rearrangement of immunoglobulin and T‐cell receptor genes in a ha‐ras‐transformed lymphoid progenitor cell line. Proc Natl Acad Sci USA 84: 1799–1803 3470759 10.1073/pnas.84.7.1799 PMC304528

[msb202211037-bib-0002] Beer LA , Liu P , Ky B , Barnhart KT , Speicher DW (2017) Efficient quantitative comparisons of plasma proteomes using label‐free analysis with MaxQuant. Methods Mol Biol 1619: 339–352 28674895 10.1007/978-1-4939-7057-5_23 PMC5575765

[msb202211037-bib-0003] Bennett MJ , Choe S , Eisenberg D (1994) Domain swapping: entangling alliances between proteins. Proc Natl Acad Sci USA 91: 3127–3131 8159715 10.1073/pnas.91.8.3127 PMC43528

[msb202211037-bib-0004] Blanco JD , Radusky L , Climente‐González H , Serrano L (2018) FoldX accurate structural protein–DNA binding prediction using PADA1 (protein assisted DNA assembly 1). Nucleic Acids Res 46: 3852–3863 29608705 10.1093/nar/gky228 PMC5934639

[msb202211037-bib-0005] Braat H , Rottiers P , Hommes DW , Huyghebaert N , Remaut E , Remon J , van Deventer SJH , Neirynck S , Peppelenbosch MP , Steidler L (2006) A phase I trial with transgenic bacteria expressing Interleukin‐10 in Crohn's disease. Clin Gastroenterol Hepatol 4: 754–759 16716759 10.1016/j.cgh.2006.03.028

[msb202211037-bib-0006] Broto A , Gaspari E , Miravet‐Verde S , Dos Santos VAPM , Isalan M (2022) A genetic toolkit and gene switches to limit mycoplasma growth for biosafety applications. Nat Commun 13: 1910 35393441 10.1038/s41467-022-29574-0 PMC8991246

[msb202211037-bib-0007] Burgos R , Weber M , Martinez S , Lluch‐Senar M , Serrano L (2020) Protein quality control and regulated proteolysis in the genome‐reduced organism *Mycoplasma pneumoniae* . Mol Syst Biol 16: e9530 33320415 10.15252/msb.20209530 PMC7737663

[msb202211037-bib-0008] Cardoso A , Gil Castro A , Martins AC , Carriche GM , Murigneux V , Castro I , Cumano A , Vieira P , Saraiva M (2018) The dynamics of Interleukin‐10‐afforded protection during dextran sulfate sodium‐induced colitis. Front Immunol 9: 400 29545807 10.3389/fimmu.2018.00400 PMC5837963

[msb202211037-bib-0009] Chiva C , Olivella R , Borràs E , Espadas G , Pastor O , Solé A , Sabidó E (2018) QCloud: a cloud‐based quality control system for mass spectrometry‐based proteomics laboratories. PLoS ONE 13: e0189209 29324744 10.1371/journal.pone.0189209 PMC5764250

[msb202211037-bib-0010] Cianferoni D , Radusky LG , Head SA , Serrano L , Delgado J (2020) ProteinFishing: a protein complex generator within the ModelX toolsuite. Bioinformatics 36: 4208–4210 32437555 10.1093/bioinformatics/btaa533 PMC7390992

[msb202211037-bib-0011] Cypel M , Liu M , Rubacha M , Yeung JC , Hirayama S , Anraku M , Sato M , Medin J , Davidson BL , de Perrot M *et al* (2009) Functional repair of human donor lungs by IL‐10 gene therapy. Sci Transl Med 1: 4ra9 10.1126/scitranslmed.3000266 20368171

[msb202211037-bib-0012] Delgado Blanco J , Radusky LG , Cianferoni D , Serrano L (2019) Protein‐assisted RNA fragment docking (RnaX) for modeling RNA‐protein interactions using ModelX. Proc Natl Acad Sci USA 116: 24568–24573 31732673 10.1073/pnas.1910999116 PMC6900601

[msb202211037-bib-0013] Delgado J , Radusky LG , Cianferoni D , Serrano L (2019) FoldX 5.0: working with RNA, small molecules and a new graphical interface. Bioinformatics 35: 4168–4169 30874800 10.1093/bioinformatics/btz184 PMC6792092

[msb202211037-bib-0014] Duncan SA , Dixit S , Sahu R , Martin D , Baganizi DR , Nyairo E , Villinger F , Singh SR , Dennis VA (2019) Prolonged release and functionality of Interleukin‐10 encapsulated within PLA‐PEG nanoparticles. Nanomaterials (Basel) 9: 1074 31357440 10.3390/nano9081074 PMC6723354

[msb202211037-bib-0015] Fillatreau S , O'Garra A (2014) Interleukin‐10 in health and disease. Berlin: Springer

[msb202211037-bib-0016] Gaidt MM , Rapino F , Graf T , Hornung V (2018) Modeling primary human monocytes with the trans‐differentiation cell line BLaER1. Methods Mol Biol 1714: 57–66 29177855 10.1007/978-1-4939-7519-8_4

[msb202211037-bib-0017] Garrido V , Piñero‐Lambea C , Rodriguez‐Arce I , Paetzold B , Ferrar T , Weber M , Garcia‐Ramallo E , Gallo C , Collantes M , Peñuelas I *et al* (2021) Engineering a genome‐reduced bacterium to eliminate *Staphylococcus aureus* biofilms *in vivo* . Mol Syst Biol 17: e10145 34612607 10.15252/msb.202010145 PMC8493563

[msb202211037-bib-0018] Gessulat S , Schmidt T , Zolg DP , Samaras P , Schnatbaum K , Zerweck J , Knaute T , Rechenberger J , Delanghe B , Huhmer A *et al* (2019) Prosit: proteome‐wide prediction of peptide tandem mass spectra by deep learning. Nat Methods 16: 509–518 31133760 10.1038/s41592-019-0426-7

[msb202211037-bib-0019] Gibson DG , Young L , Chuang R‐Y , Venter JC , Hutchison CA III , Smith HO (2009) Enzymatic assembly of DNA molecules up to several hundred kilobases. Nat Methods 6: 343–345 19363495 10.1038/nmeth.1318

[msb202211037-bib-0020] Gorby C , Sotolongo Bellón J , Wilmes S , Warda W , Pohler E , Fyfe PK , Cozzani A , Ferrand C , Walter MR , Mitra S *et al* (2020) Engineered IL‐10 variants elicit potent immunomodulatory effects at low ligand doses. Sci Signal 13: eabc0653 32934073 10.1126/scisignal.abc0653 PMC7685028

[msb202211037-bib-0021] Grilló M‐J , Blasco JM , Gorvel JP , Moriyón I , Moreno E (2012) What have we learned from brucellosis in the mouse model? Vet Res 43: 29 22500859 10.1186/1297-9716-43-29 PMC3410789

[msb202211037-bib-0022] Hames C , Halbedel S , Hoppert M , Frey J , Stülke J (2009) Glycerol metabolism is important for cytotoxicity of mycoplasma pneumoniae. J Bacteriol 191: 747–753 19028882 10.1128/JB.01103-08 PMC2632104

[msb202211037-bib-0023] Heck HD (1971) Statistical theory of cooperative binding to proteins. Hill equation and the binding potential. J Am Chem Soc 93: 23–29 5538860 10.1021/ja00730a004

[msb202211037-bib-0024] Hirche TO , Benabid R , Deslee G , Gangloff S , Achilefu S , Guenounou M , Lebargy F , Hancock RE , Belaaouaj A (2008) Neutrophil elastase mediates innate host protection against *Pseudomonas aeruginosa* . J Immunol 181: 4945–4954 18802098 10.4049/jimmunol.181.7.4945

[msb202211037-bib-0025] Huffnagle GB , Dickson RP , Lukacs NW (2017) The respiratory tract microbiome and lung inflammation: a two‐way street. Mucosal Immunol 10: 299–306 27966551 10.1038/mi.2016.108 PMC5765541

[msb202211037-bib-0026] Inamine JM , Ho KC , Loechel S , Hu PC (1990) Evidence that UGA is read as a tryptophan codon rather than as a stop codon by *Mycoplasma pneumoniae*, *Mycoplasma genitalium*, and *Mycoplasma gallisepticum* . J Bacteriol 172: 504–506 2104612 10.1128/jb.172.1.504-506.1990 PMC208464

[msb202211037-bib-0027] Josephson K , DiGiacomo R , Indelicato SR , Iyo AH , Nagabhushan TL , Parker MH , Walter MR (2000) Design and analysis of an engineered human interleukin‐10 monomer. J Biol Chem 275: 13552–13557 10788470 10.1074/jbc.275.18.13552

[msb202211037-bib-0028] Kamei A , Gao G , Neale G , Loh LN , Vogel P , Thomas PG , Tuomanen EI , Murray PJ (2016) Exogenous remodeling of lung resident macrophages protects against infectious consequences of bone marrow‐suppressive chemotherapy. Proc Natl Acad Sci USA 113: E6153–E6161 27671632 10.1073/pnas.1607787113 PMC5068317

[msb202211037-bib-0029] Kühner S , van Noort V , Betts MJ , Leo‐Macias A , Batisse C , Rode M , Yamada T , Maier T , Bader S , Beltran‐Alvarez P *et al* (2009) Proteome organization in a genome‐reduced bacterium. Science 326: 1235–1240 19965468 10.1126/science.1176343

[msb202211037-bib-0031] Lindner HA , Velásquez SY , Thiel M , Kirschning T (2021) Lung protection vs. infection resolution: interleukin 10 suspected of double‐dealing in COVID‐19. Front Immunol 12: 602130 33746948 10.3389/fimmu.2021.602130 PMC7966717

[msb202211037-bib-0032] Maier T , Schmidt A , Güell M , Kühner S , Gavin A , Aebersold R , Serrano L (2011) Quantification of mRNA and protein and integration with protein turnover in a bacterium. Mol Syst Biol 7: 511 21772259 10.1038/msb.2011.38 PMC3159969

[msb202211037-bib-0033] Mauras A , Chain F , Faucheux A , Ruffié P , Gontier S , Ryffel B , Butel M‐J , Langella P , Bermúdez‐Humarán LG , Waligora‐Dupriet A‐J (2018) A new Bifidobacteria expression SysTem (BEST) to produce and deliver Interleukin‐10 in *Bifidobacterium bifidum* . Front Microbiol 9: 3075 30622516 10.3389/fmicb.2018.03075 PMC6308194

[msb202211037-bib-0034] Minshawi F , Lanvermann S , McKenzie E , Jeffery R , Couper K , Papoutsopoulou S , Roers A , Muller W (2020) The generation of an engineered Interleukin‐10 protein with improved stability and biological function. Front Immunol 11: 1794 32849644 10.3389/fimmu.2020.01794 PMC7431522

[msb202211037-bib-0035] Ouyang W , O'Garra A (2019) IL‐10 family cytokines IL‐10 and IL‐22: from basic science to clinical translation. Immunity 50: 871–891 30995504 10.1016/j.immuni.2019.03.020

[msb202211037-bib-0036] Perkins DN , Pappin DJ , Creasy DM , Cottrell JS (1999) Probability‐based protein identification by searching sequence databases using mass spectrometry data. Electrophoresis 20: 3551–3567 10612281 10.1002/(SICI)1522-2683(19991201)20:18<3551::AID-ELPS3551>3.0.CO;2-2

[msb202211037-bib-0037] Piñero‐Lambea C , Garcia‐Ramallo E , Martinez S , Delgado J , Serrano L , Lluch‐Senar M (2020) Genome editing based on oligo recombineering and Cas9‐mediated counterselection. ACS Synth Biol 9: 1693–1704 32502342 10.1021/acssynbio.0c00022 PMC7372593

[msb202211037-bib-0038] Saraiva M , Vieira P , O'Garra A (2020) Biology and therapeutic potential of interleukin‐10. J Exp Med 217: e20190418 31611251 10.1084/jem.20190418 PMC7037253

[msb202211037-bib-0039] Saxena A , Khosraviani S , Noel S , Mohan D , Donner T , Hamad ARA (2015) Interleukin‐10 paradox: a potent immunoregulatory cytokine that has been difficult to harness for immunotherapy. Cytokine 74: 27–34 25481648 10.1016/j.cyto.2014.10.031 PMC4454631

[msb202211037-bib-0040] Saxton RA , Tsutsumi N , Su LL , Abhiraman GC , Mohan K , Henneberg LT , Aduri NG , Gati C , Garcia KC (2021) Structure‐based decoupling of the pro‐ and anti‐inflammatory functions of interleukin‐10. Science 371: eabc8433 33737461 10.1126/science.abc8433 PMC9132103

[msb202211037-bib-0041] Schymkowitz J , Borg J , Stricher F , Nys R , Rousseau F , Serrano L (2005) The FoldX web server: an online force field. Nucleic Acids Res 33: W382–W388 15980494 10.1093/nar/gki387 PMC1160148

[msb202211037-bib-0042] Scott M , Gunderson CW , Mateescu EM , Zhang Z , Hwa T (2010) Interdependence of cell growth and gene expression: origins and consequences. Science 330: 1099–1102 21097934 10.1126/science.1192588

[msb202211037-bib-0043] Segovia JA , Bose S , Somarajan SR , Chang T‐H , Kannan T , Baseman JB (2015) *Mycoplasma pneumoniae* Cards toxin regulates NLRP3 inflammasome activation. J Allergy Clin Immunol 135: AB153 10.1128/mBio.02186-14 PMC4278538 25538194

[msb202211037-bib-0044] Shamskhou EA , Kratochvil MJ , Orcholski ME , Nagy N , Kaber G , Steen E , Balaji S , Yuan K , Keswani S , Danielson B *et al* (2019) Hydrogel‐based delivery of Il‐10 improves treatment of bleomycin‐induced lung fibrosis in mice. Biomaterials 203: 52–62 30852423 10.1016/j.biomaterials.2019.02.017 PMC6430662

[msb202211037-bib-0045] Sluijter M , Kaptein E , Spuesens EBM , Hoogenboezem T , Hartwig NG , Van Rossum AMC , Vink C (2010) The *Mycoplasma genitalium* MG352‐encoded protein is a Holliday junction resolvase that has a non‐functional orthologue in *Mycoplasma pneumoniae* . Mol Microbiol 77: 1261–1277 20735784 10.1111/j.1365-2958.2010.07288.x

[msb202211037-bib-0046] Somarajan SR , Kannan TR , Baseman JB (2010) *Mycoplasma pneumoniae* Mpn133 is a cytotoxic nuclease with a glutamic acid‐, lysine‐ and serine‐rich region essential for binding and internalization but not enzymatic activity. Cell Microbiol 12: 1821–1831 20690923 10.1111/j.1462-5822.2010.01513.x PMC3013625

[msb202211037-bib-0047] Steidler L , Hans W , Schotte L , Neirynck S , Obermeier F , Falk W , Fiers W , Remaut E (2000) Treatment of murine colitis by *Lactococcus lactis* secreting interleukin‐10. Science 289: 1352–1355 10958782 10.1126/science.289.5483.1352

[msb202211037-bib-0048] Sun L , Guo R‐F , Newstead MW , Standiford TJ , Macariola DR , Shanley TP (2009) Effect of IL‐10 on neutrophil recruitment and survival after *Pseudomonas aeruginosa* challenge. Am J Respir Cell Mol Biol 41: 76–84 19097982 10.1165/rcmb.2008-0202OC PMC2701962

[msb202211037-bib-0049] Syto R , Murgolo NJ , Braswell EH , Mui P , Huang E , Windsor WT (1998) Structural and biological stability of the human interleukin 10 homodimer. Biochemistry 37: 16943–16951 9836587 10.1021/bi981555y

[msb202211037-bib-0050] Tamiya S , Yoshikawa E , Suzuki K , Yoshioka Y (2021) Susceptibility analysis in several mouse strains reveals robust T‐cell responses after *Mycoplasma pneumoniae* infection in DBA/2 mice. Front Cell Infect Microbiol 10: 602453 33520736 10.3389/fcimb.2020.602453 PMC7839406

[msb202211037-bib-0051] Terai M , Tamura Y , Alexeev V , Ohtsuka E , Berd D , Mastrangelo MJ , Sato T (2009) Human interleukin 10 receptor 1/IgG1‐fc fusion proteins: immunoadhesins for human IL‐10 with therapeutic potential. Cancer Immunol Immunother 58: 1307–1317 19142637 10.1007/s00262-008-0644-9 PMC11030067

[msb202211037-bib-0052] Troegeler A , Lastrucci C , Duval C , Tanne A , Cougoule C , Maridonneau‐Parini I , Neyrolles O , Lugo‐Villarino G (2014) An efficient siRNA‐mediated gene silencing in primary human monocytes, dendritic cells and macrophages. Immunol Cell Biol 92: 699–708 24890643 10.1038/icb.2014.39

[msb202211037-bib-0053] van Deventer SJ , Elson CO , Fedorak RN (1997) Multiple doses of intravenous interleukin 10 in steroid‐refractory Crohn's disease. Crohn's Disease Study Group. Gastroenterology 113: 383–389 9247454 10.1053/gast.1997.v113.pm9247454

[msb202211037-bib-0054] Waites KB , Talkington DF (2004) Mycoplasma pneumoniae and its role as a human pathogen. Clin Microbiol Rev 17: 697–728 15489344 10.1128/CMR.17.4.697-728.2004 PMC523564

[msb202211037-bib-0055] Wang X , Wong K , Ouyang W , Rutz S (2019) Targeting IL‐10 family cytokines for the treatment of human diseases. Cold Spring Harb Perspect Biol 11: a028548 29038121 10.1101/cshperspect.a028548 PMC6360861

[msb202211037-bib-0056] Westerhof LB , Wilbers RHP , Roosien J , van de Velde J , Goverse A , Bakker J , Schots A (2012) 3D domain swapping causes extensive multimerisation of human Interleukin‐10 when expressed In planta. PLoS ONE 7: e46460 23049703 10.1371/journal.pone.0046460 PMC3462211

[msb202211037-bib-0057] Wodke JAH , Puchałka J , Lluch‐Senar M , Marcos J , Yus E , Godinho M , Gutiérrez‐Gallego R , dos Santos VAPM , Serrano L , Klipp E *et al* (2013) Dissecting the energy metabolism in *Mycoplasma pneumoniae* through genome‐scale metabolic modeling. Mol Syst Biol 9: 653 23549481 10.1038/msb.2013.6 PMC3658275

[msb202211037-bib-0058] Yu H , Pardoll D , Jove R (2009) STATs in cancer inflammation and immunity: a leading role for STAT3. Nat Rev Cancer 9: 798–809 19851315 10.1038/nrc2734 PMC4856025

[msb202211037-bib-0059] Yus E , Yang J‐S , Sogues A , Serrano L (2017) A reporter system coupled with high‐throughput sequencing unveils key bacterial transcription and translation determinants. Nat Commun 8: 368 28848232 10.1038/s41467-017-00239-7 PMC5573727

