# Author Correction: Bacterial expression of a designed single-chain IL-10 prevents severe lung inflammation

**DOI:** 10.1038/s44320-023-00008-3

**Published:** 2024-02-13

**Authors:** Ariadna Montero-Blay, Javier Delgado Blanco, Irene Rodriguez-Arce, Claire Lastrucci, Carlos Piñero-Lambea, Maria Lluch-Senar, Luis Serrano

**Affiliations:** 1 Centre for Genomic Regulation (CRG), The Barcelona Institute of Science and Technology, Dr. Aiguader 88, Barcelona, 08003 Spain; 2 Universitat Pompeu Fabra (UPF), Barcelona, 08002 Spain; 3 ICREA, Pg. Lluís Companys 23, Barcelona, 08010 Spain

## Abstract

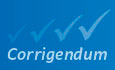

Correction to: *Molecular Systems Biology* (2023) 19:e11037. 10.15252/msb.202211037 | Published online 4 January 2023

**Figure 3B is incorrect.**

**Dataset EV5 is incorrect.**

The authors noticed a mistake in two of the mutations for MutSC2 shown in Fig. 3B. S31L and N92L are corrected to S31K and N92I respectively. The corrected and original figures are shown below.

**Figure 3B:**
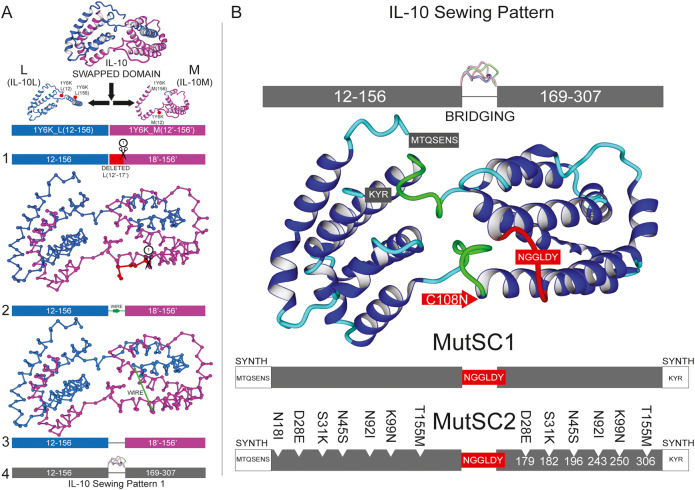
Corrected.

**Figure 3B:**
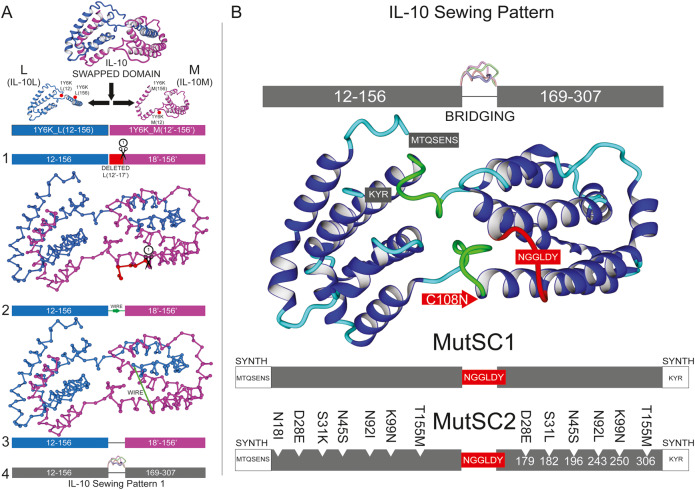
Original.

Dataset EV5 has been updated to remove the molecule name (L, for IL-10L monomer) that was erroneously inserted in the description of the mutants (i.e. DL28E has been corrected to D28E etc. in the sheet “Mutant Details”). The corrected and original Dataset EV5 are available with this correction.

These errors do not affect the conclusions of the original paper. All authors agree to this correction. The authors apologize for these errors.

### Supplementary information

Dataset ORIGINAL EV5

Dataset CORRECTED EV5

