# Supplementary material for: Bacterial expression of a designed single‐chain IL‐10 prevents severe lung inflammation
Source: Mol Syst Biol. 2023 Jan 4;19(1):e11037. doi: 10.15252/msb.202211037 (PMC9834763; doi:10.15252/msb.202211037)
Supplement: Supplementary file 1 — Expanded View Figures PDF [file MSB-19-e11037-s014.pdf]

Expanded View Figures

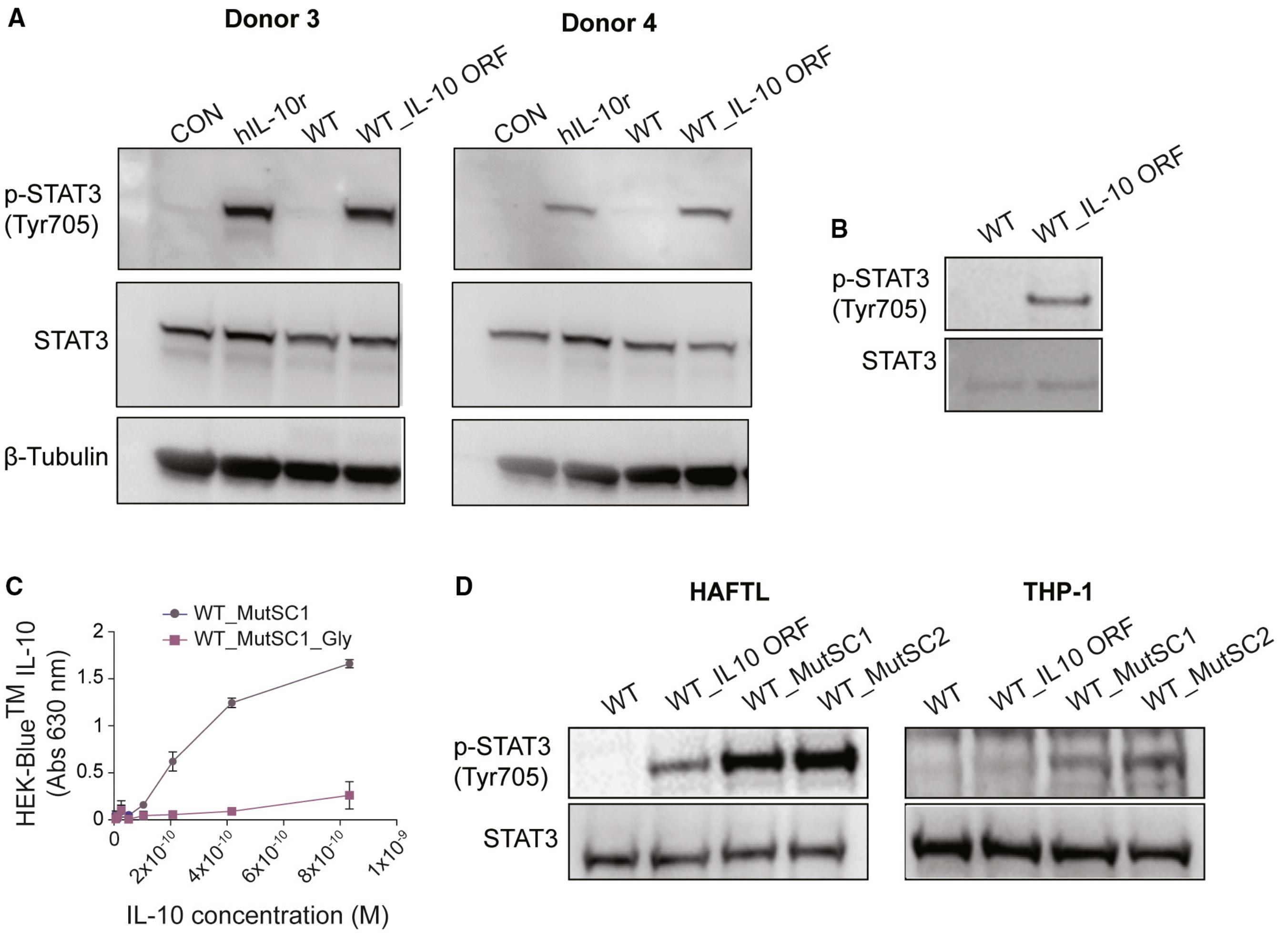

Figure EV1. Analysis of SC mutants designed in this work.

A, B Detection of phosphorylated Tyr 705 of STAT3 (p-STAT3) and unphosphorylated STAT3 by Western blot (see Materials and Methods). (A) Data from macrophages isolated from two independent donors, showing unstimulated cells (control, CON) or cells incubated for 24 h with human IL-10 recombinant protein (hIL-10r), supernatant of *MPN* WT (WT) or of *MPN* expressing IL-10 (WT_IL-10 ORF). (B) Data from HAFTL murine B-cell line after exposure for 20 min to the supernatant of *MPN* WT (WT) or *MPN* expressing IL-10 (WT_IL-10 ORF).

C HEK-Blue™ reporter cell activation dose–response analysis by MutSC1 (linker NGGLD) and MutSC1_Gly (linker GGGGG) supernatants. The *x*-axis shows the range of IL-10 concentration analysed (Molar, M), and the *y*-axis represents the mean ± SD of the absorbance at 630 nm. Data were generated in three independent assays with two technical replicas ($n > 6$).

D Western blot of p-STAT3 activation after 20 min of induction with a fixed IL-10 concentration (20 ng/ml) of supernatants from *MPN* WT (WT) or *MPN* expressing IL-10 WT (WT_IL10 ORF), MutSC1 (WT_MutSC1), MutSC2 (WT_MutSC2) in two different cell lines: THP-1 (human monocyte) and HAFTL (murine pre-B cell line).

© 2023 The Authors

```
IL-10human        1  SPGQGTQSENSCTHFPGNLPNMLRDLRDAFSRVKTFFQMKDQLDNLLLKESLLEDFKGYL
IL-10mice         1  SRGQYSREDNNCTHFPVGQSHMLLELRTAFSQVKTFFQTKDQLDNILLTDSLMQDFKGYL
IL-10macaque      1  SPGQGTQSENSCTRFPGNLPHMLRDLRDAFSRVKTFFQMKDQLDNILLKESLLEDFKGYL
IL-10chicken      1  LEPTCLHFSELLPARLRELRVKFEEIKDYFQSRDDELNIQLLSSELLDEFKGTFGCQSVS
IL-10guineapig    1  SQGTNTQSEDSCAHFPAGLPHMLRELRAAFGRVKTFFQTQDQLDNVLLNKSLLEDFKGYL
IL-10daniorerio   1  RRVECKTDCCSFVEGFPLRLRELRSAYKEIQKFYESNDDLEPLLNEDIKHNINSPYGCHV

IL-10human       61  GCQALSEMIQFYLEEVMPQAENQDPDIKAHVNSLGENLKTLRLRLRRCHRFLPCENKSKA
IL-10mice        61  GCQALSEMIQFYLVEVMPQAEKHGPEIKEHLNSLGEKLKTLRMRLRRCHRFLPCENKSKA
IL-10macaque     61  GCQALSEMIQFYLEEVMPQAENHDPDIKEHVNSLGENLKTLRLRLRRCHRFLPCENKSKA
IL-10chicken     61  EMLRFYTDEVLPRAMQTSTSHQQSMGDLGNMLLGLKATMRRCHRFFTCEKRSKAIKQIKE
IL-10guineapig   61  GCQALSEMIQFYLVEVMPQAEKHGPEIKEHLNSLGEKLKTLRMRLRRCHRFLPCENKSKA
IL-10daniorerio  61  MNEILHFYLETILPTALQKNPLKHSTTPIDSIGNIFQELKRDMVKCKRYFSCQNPFEVNS

IL-10human      121  VEQVKNAFNKLQEKGIYKAMSEFDIFINYIEAYMTMKIRN
IL-10mice       121  VEQVKSDFNKLQDQGVYKAMNEFDIFINCIEAYMMIKMKS
IL-10macaque    121  VEQVKNAFSKLQEKGVYKAMSEFDIFINYIEAYMTMKIQN
IL-10chicken    121  TFEKMDENGIYKAMGEFDIFINYIEEYLLMRRRK------
IL-10guineapig  121  VEQVKSDFNKLQDQGVYKAMNEFDIFINCIEAYMMIKMKS
IL-10daniorerio 121  LKNSYEKMKEKGVYKAMGELDLLFRYIEQYLASKRVKH--
```

Figure EV2. Multiple sequence alignment of IL-10 from different vertebrate species performed with the ClustalX algorithm.

The first residue corresponds to the first residue of IL-10 in the crystal structure 1y6k.

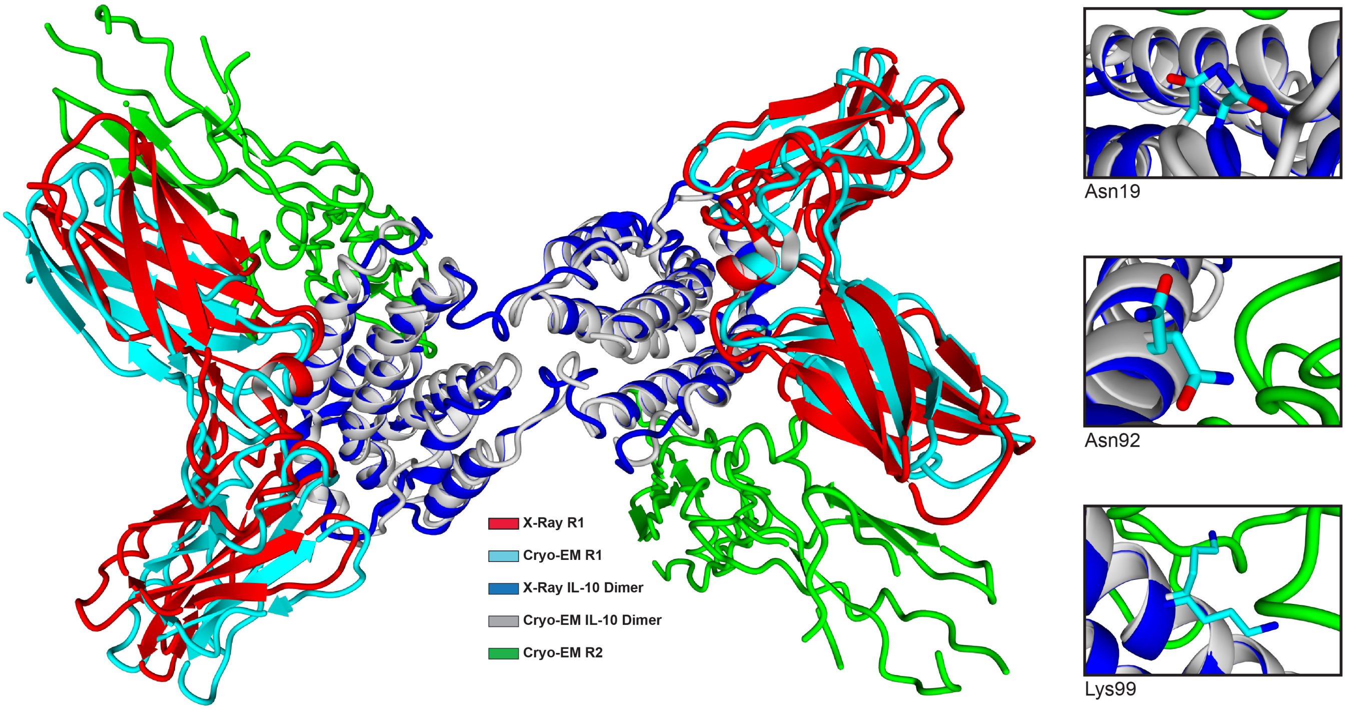

Figure EV3. Superimposition of the crystal structures of IL-10 with R1 (1yl6k) and of IL-10 with both R1 and R2 (6x93).

Unstructured regions adopted a different conformation when interacting with R2. Gorby (9)-mutated positions (N19, N92, K99) are zoomed-in on the right side in the crystallographic superimposition with cryo-EM (9), showing an appreciable backbone shifting upon binding to R2 in some of the positions.

 © 2023 The Authors

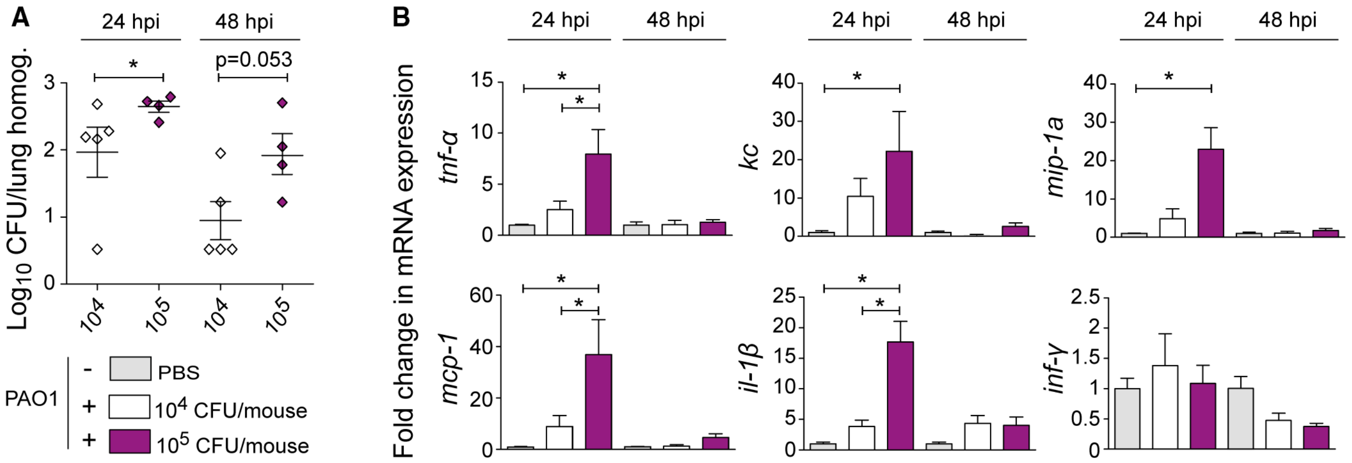

Figure EV4. Analysis of *P. aeruginosa* PAO1 infection of mice lungs.

A *Pseudomonas aeruginosa* PAO1 bacterial load obtained from mice infected with 10^4 or 10^5 CFU at 24- or 48 h post-infection (hpi). Data are shown as mean ± SD of Log_{10} CFU/lung homogenate of at least 3 mice per group ($n < 3$ biological replica). Statistical analysis was performed using one-way ANOVA + Tukey's *post hoc* test (*$P < 0.05$).

B Fold-change in mRNA expression of different inflammatory markers in the lung of mice infected with PAO1 at 24 hpi.
