# Supplementary figures and images for: Bacterial expression of a designed single‐chain IL‐10 prevents severe lung inflammation
Source: Mol Syst Biol. 2023 Jan 4;19(1):e11037. doi: 10.15252/msb.202211037 (PMC9834763; doi:10.15252/msb.202211037)

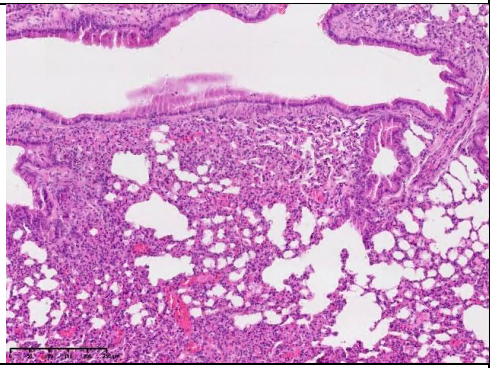

Supplement: Supplementary file 12 — Source Data for Figure 5 [file MSB-19-e11037-s006.zip › Figure 5/Figure 5C_HE stain/1. PBS_2dpi.png]

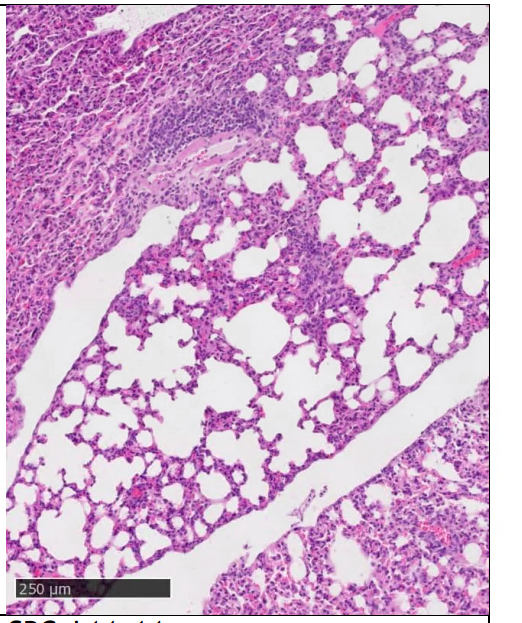

Supplement: Supplementary file 12 — Source Data for Figure 5 [file MSB-19-e11037-s006.zip › Figure 5/Figure 5C_HE stain/6. CV8_4dpi.png]

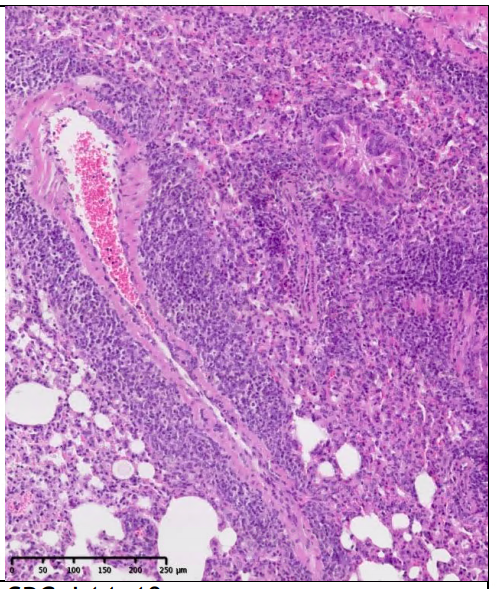

Supplement: Supplementary file 12 — Source Data for Figure 5 [file MSB-19-e11037-s006.zip › Figure 5/Figure 5C_HE stain/5. WT_4dpi.png]

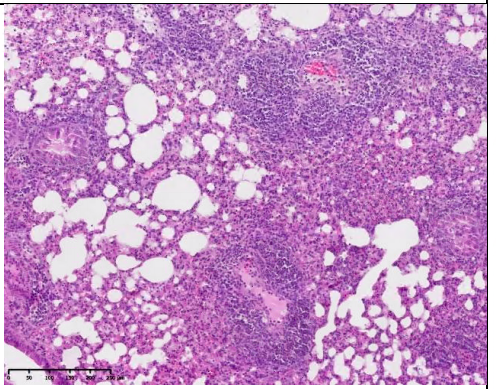

Supplement: Supplementary file 12 — Source Data for Figure 5 [file MSB-19-e11037-s006.zip › Figure 5/Figure 5C_HE stain/2. WT_2dpi.png]

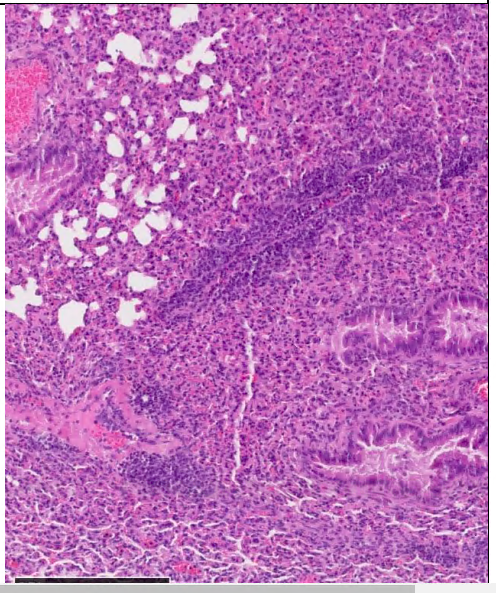

Supplement: Supplementary file 12 — Source Data for Figure 5 [file MSB-19-e11037-s006.zip › Figure 5/Figure 5C_HE stain/3. CV8_2dpi.png]

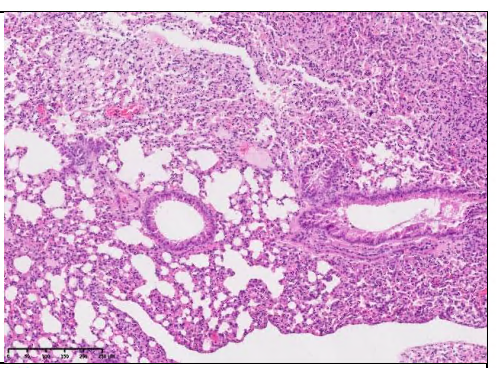

Supplement: Supplementary file 12 — Source Data for Figure 5 [file MSB-19-e11037-s006.zip › Figure 5/Figure 5C_HE stain/4. PBS_4dpi.png]

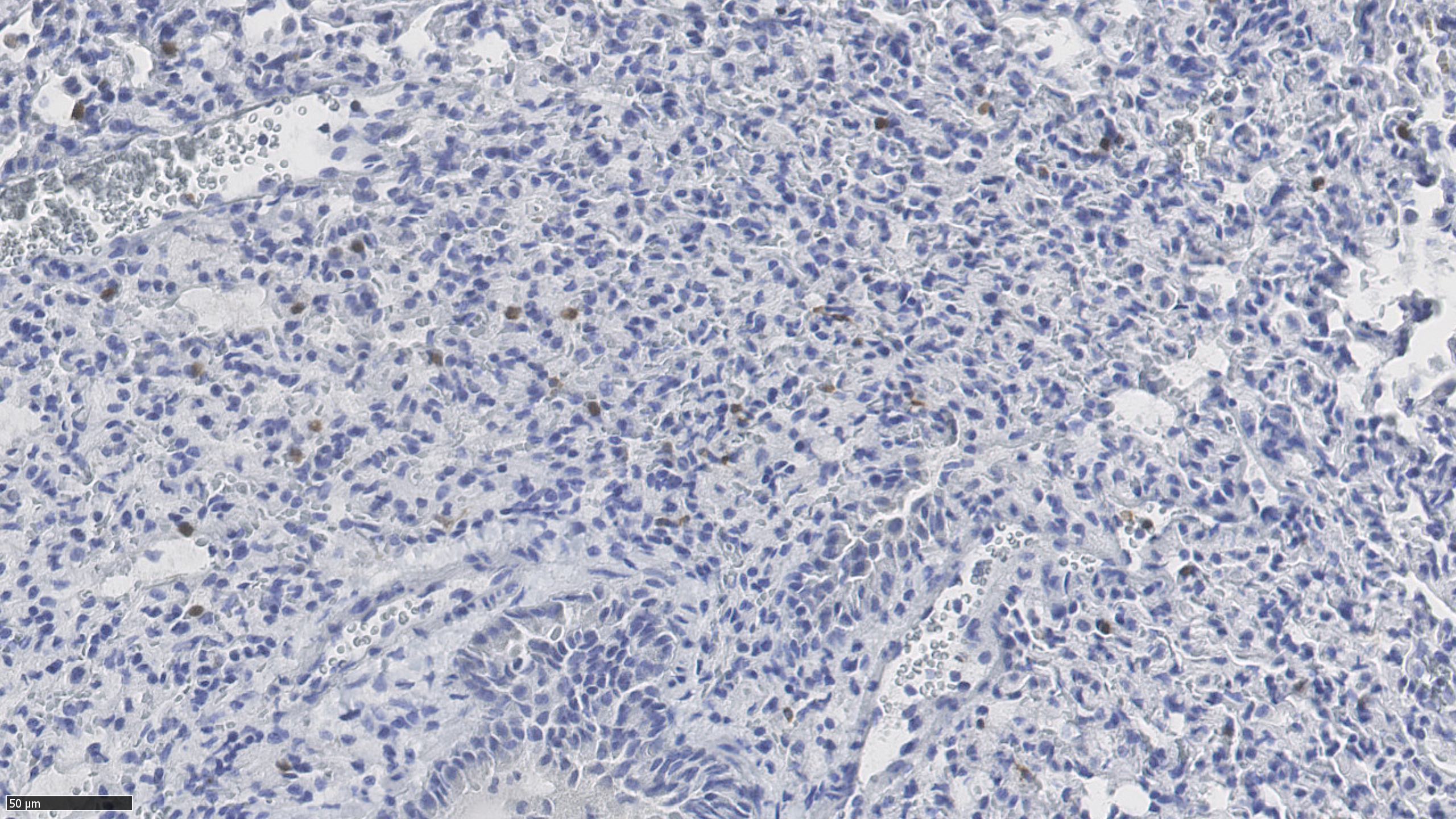

Supplement: Supplementary file 13 — Source Data for Figure 6 [file MSB-19-e11037-s011.zip › Figure 6/Figure 6E/PBS + PBS CRG_L12_55.tif]

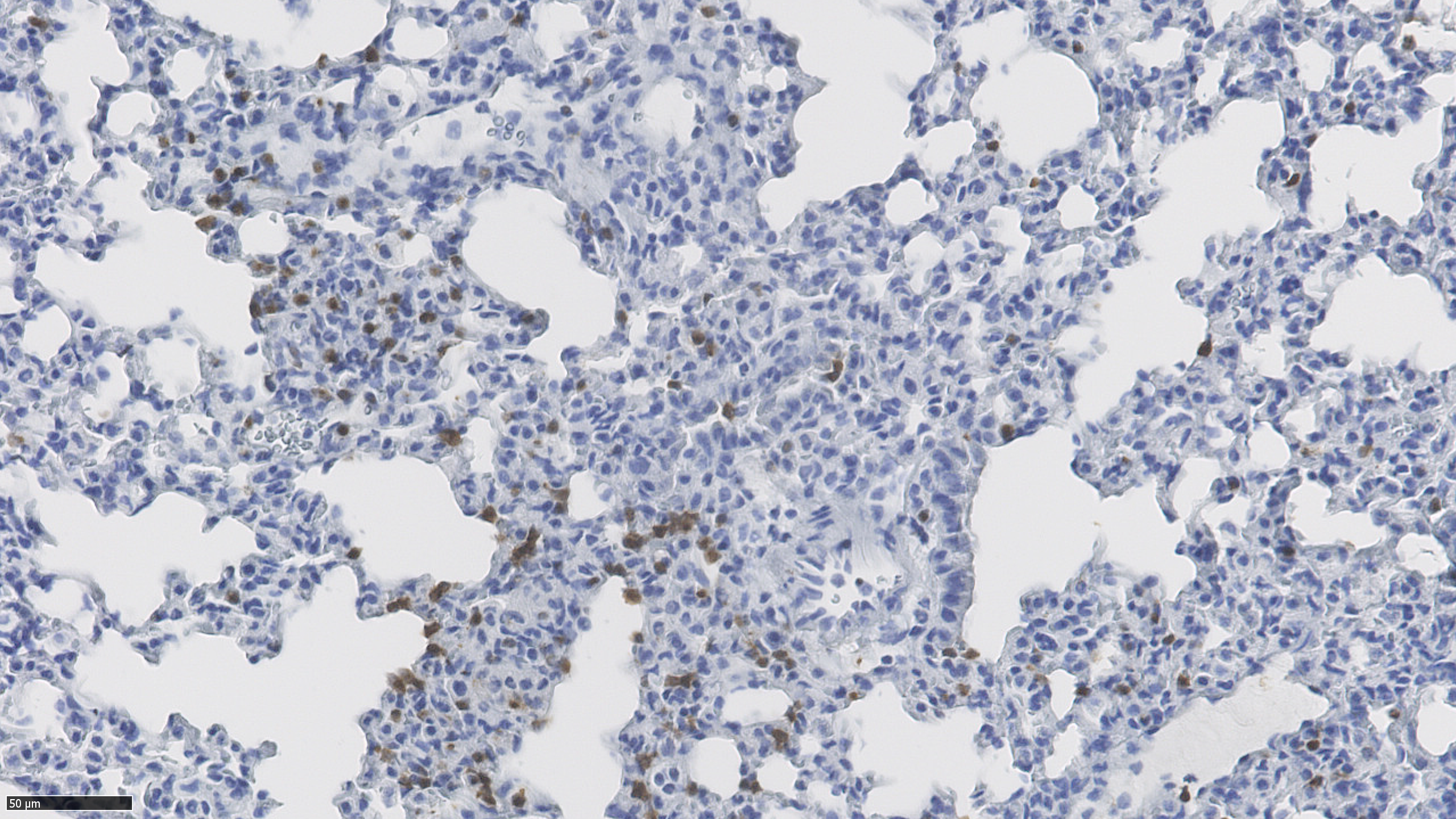

Supplement: Supplementary file 13 — Source Data for Figure 6 [file MSB-19-e11037-s011.zip › Figure 6/Figure 6E/PAO1 + CV8 F14 CRG_L12_43.tif]

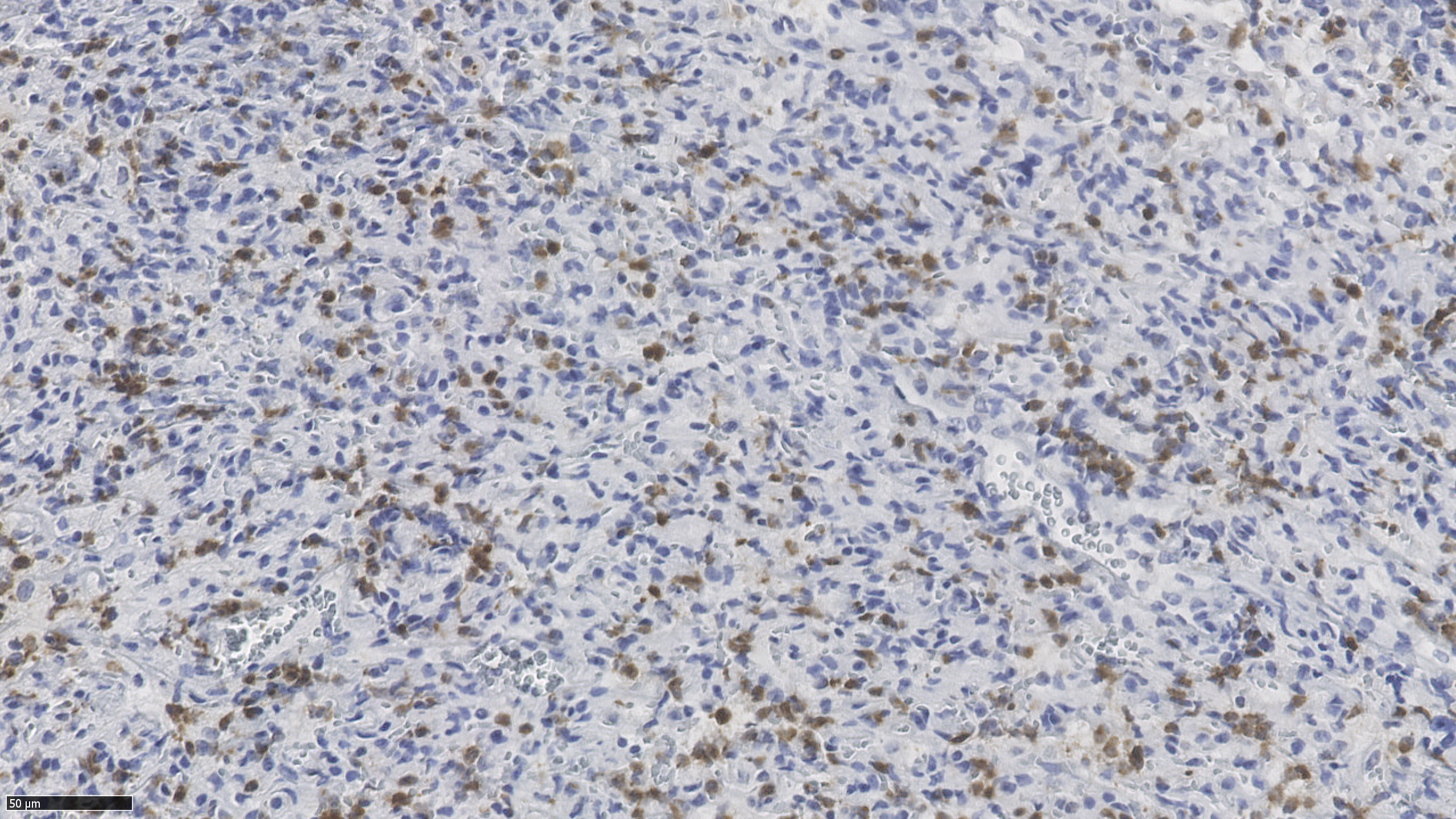

Supplement: Supplementary file 13 — Source Data for Figure 6 [file MSB-19-e11037-s011.zip › Figure 6/Figure 6E/PAO1 + hILr CRG_L12_53.tif]

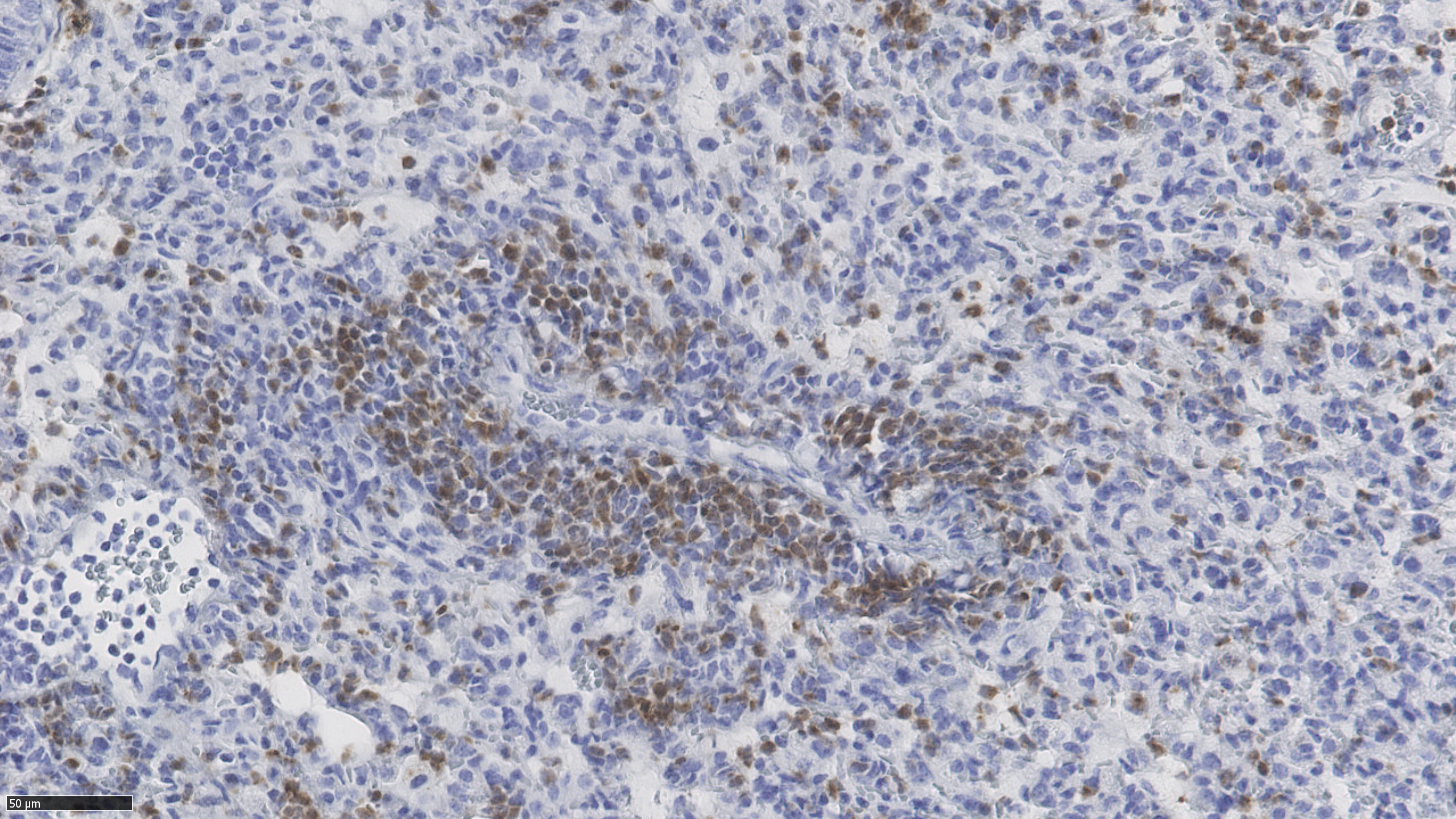

Supplement: Supplementary file 13 — Source Data for Figure 6 [file MSB-19-e11037-s011.zip › Figure 6/Figure 6E/PAO1+ PBS CRG_L12_2.tif]

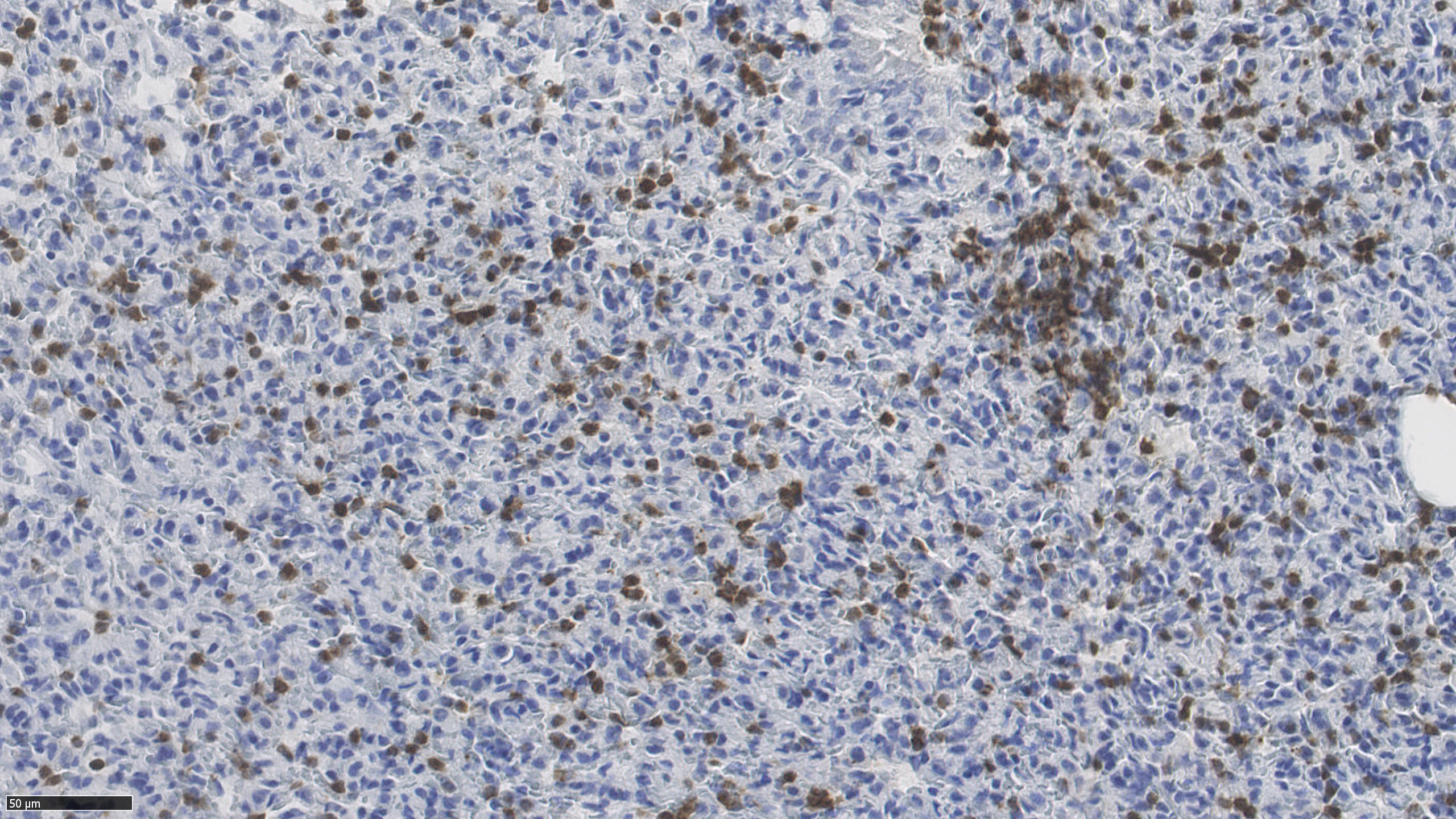

Supplement: Supplementary file 13 — Source Data for Figure 6 [file MSB-19-e11037-s011.zip › Figure 6/Figure 6E/PAO1 + ORF CRG_L12_41.tif]

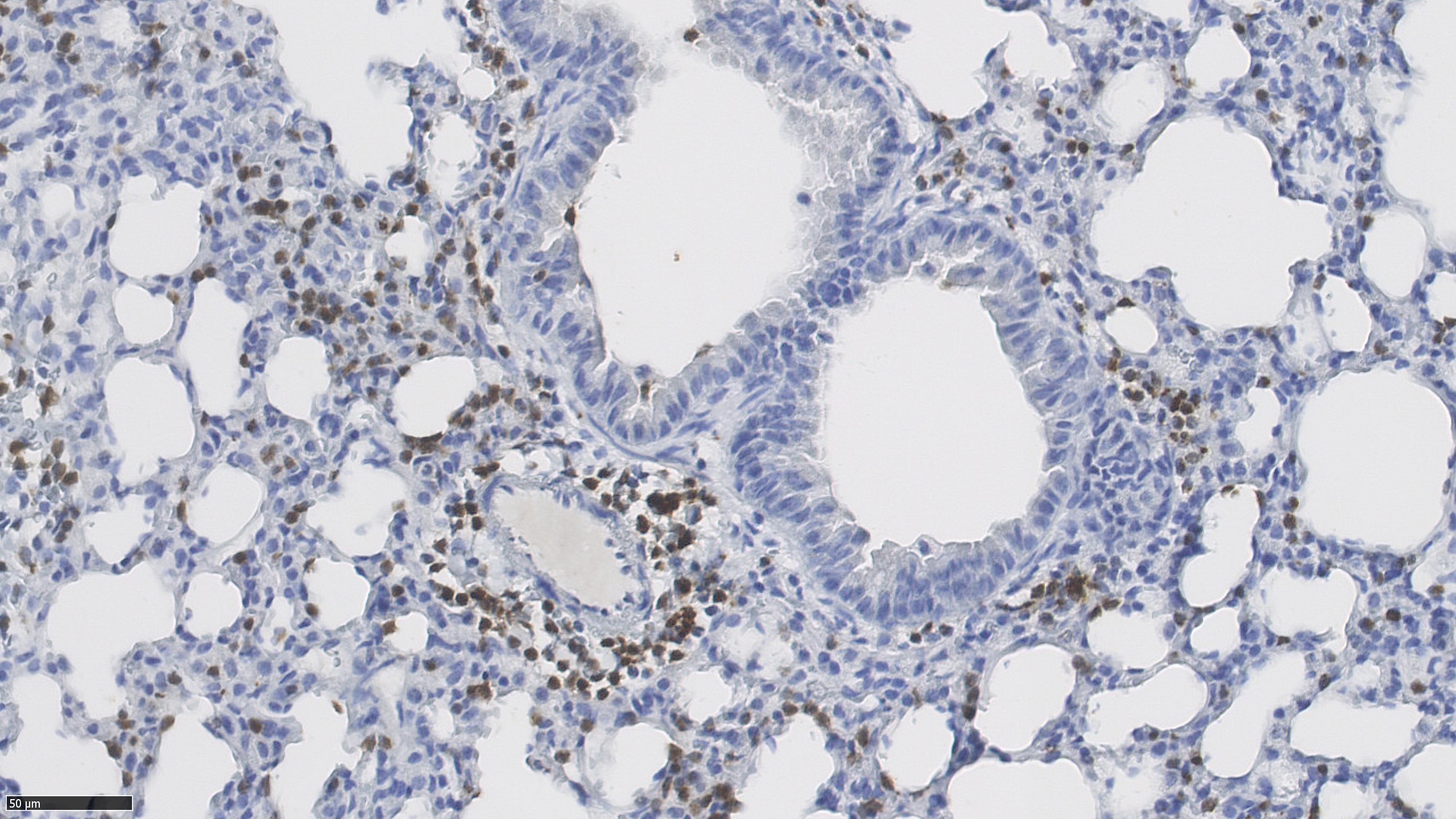

Supplement: Supplementary file 13 — Source Data for Figure 6 [file MSB-19-e11037-s011.zip › Figure 6/Figure 6E/PAO1 + CV8 F15 CRG_L12_49.tif]

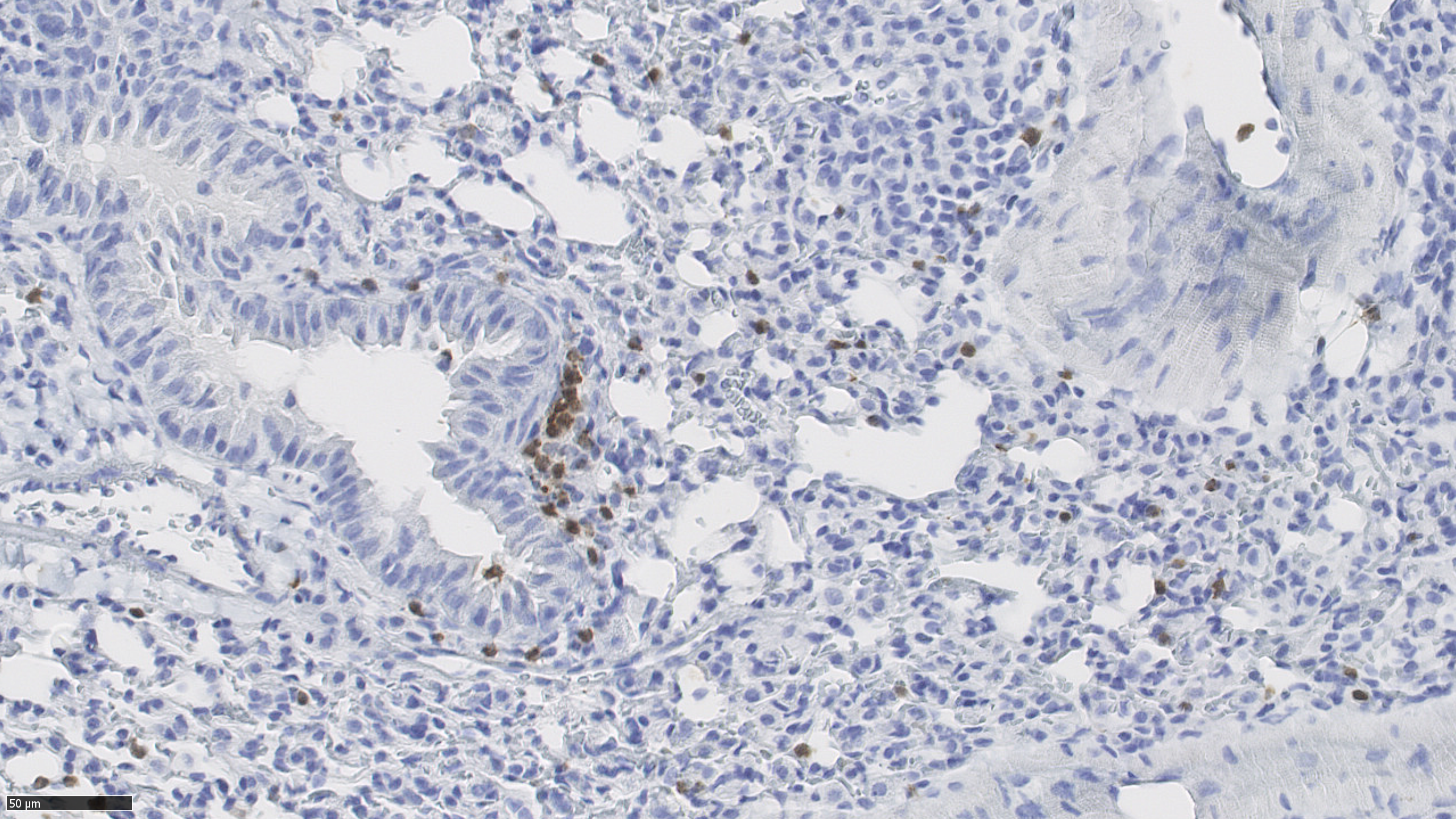

Supplement: Supplementary file 13 — Source Data for Figure 6 [file MSB-19-e11037-s011.zip › Figure 6/Figure 6E/CV8 + PBS CRG_L12_16.tif]

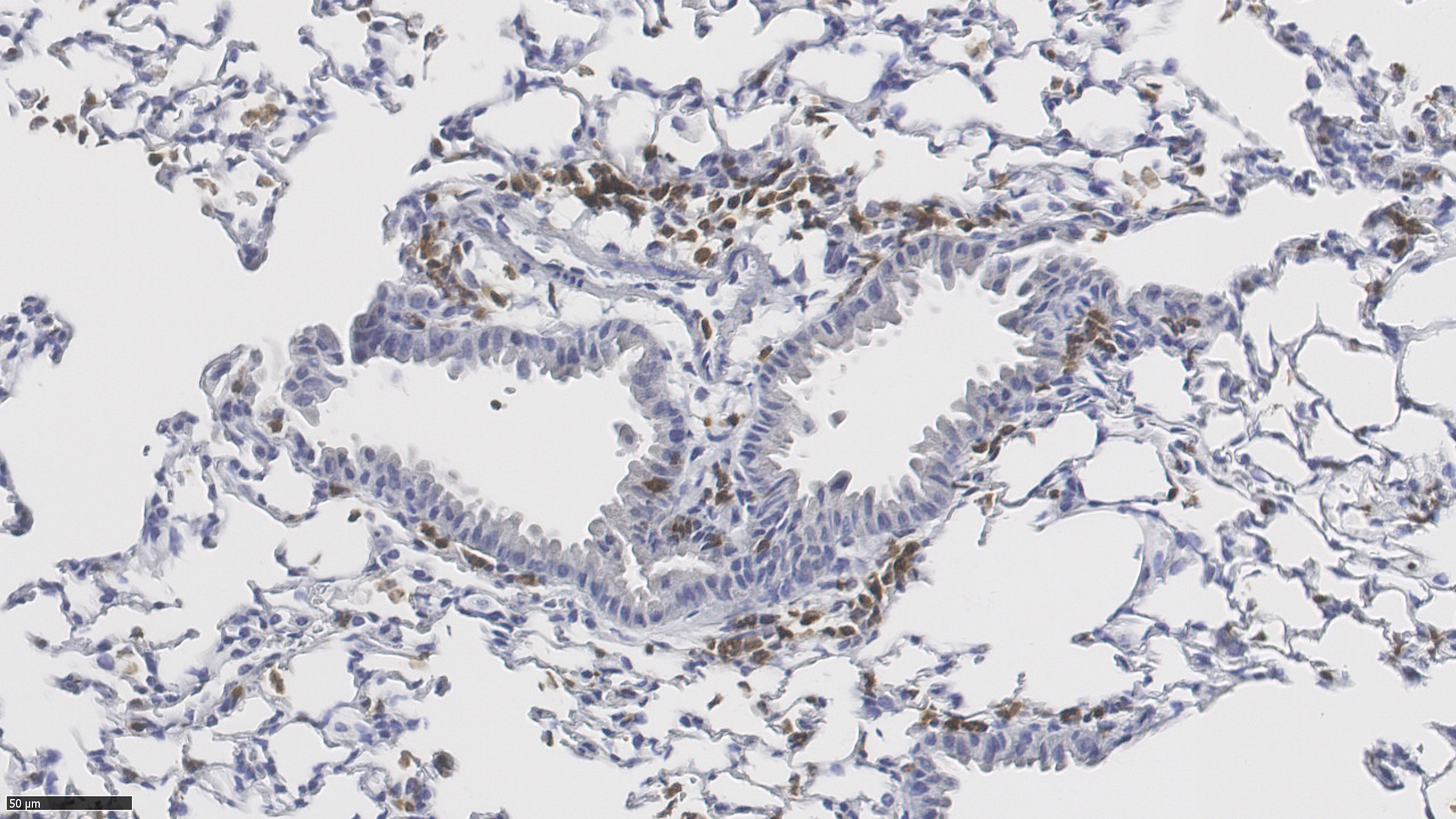

Supplement: Supplementary file 13 — Source Data for Figure 6 [file MSB-19-e11037-s011.zip › Figure 6/Figure 6E/PAO1 + CV8 CRG_L12_28.tif]
