# Supplementary material for: Bacterial expression of a designed single‐chain IL‐10 prevents severe lung inflammation
Source: Mol Syst Biol. 2023 Jan 4;19(1):e11037. doi: 10.15252/msb.202211037 (PMC9834763; doi:10.15252/msb.202211037)
Supplement: Supplementary file 14 [file MSB-19-e11037-s001.pdf]

Bacterial expression of a designed single-chain IL-10 prevents severe lung inflammation

Ariadna Montero-Blay, Javier Delgado Blanco, Irene Rodriguez-Arce, Claire Lastrucci, Carlos Piñero-Lambea, Maria Lluch-Senar, and Luis Serrano
DOI: 10.15252/msb.202211037

Corresponding author(s): Luis Serrano (luis.serrano@crg.eu) , Ariadna Montero-Blay (ariadna.montero@crg.eu)

Review Timeline:

Submission Date:	18th Mar 22
Editorial Decision:	14th Apr 22
Revision Received:	8th Aug 22
Editorial Decision:	31st Aug 22
Revision Received:	27th Sep 22
Accepted:	5th Oct 22

Editor: Jingyi Hou

Transaction Report:

(Note: With the exception of the correction of typographical or spelling errors that could be a source of ambiguity, letters and reports are not edited. Depending on transfer agreements, referee reports obtained elsewhere may or may not be included in this compilation. Referee reports are anonymous unless the Referee chooses to sign their reports.)

1st Editorial Decision 14th Apr 2022

Thank you for submitting your work to Molecular Systems Biology. We have now heard back from the three reviewers who agreed to evaluate your manuscript. As you will see from the reports below, the reviewers acknowledge the general interest and novelty of the study. They raise, however, a series of concerns, which we would ask you to address in a major revision.

I think the reviewers' recommendations are rather clear, so there is no need to repeat the points listed below. Most of the reviewers' concerns refer to the need to provide further clarifications and information (both related to the methods and experimental replicates) and to improve the presentation of the study in order to make the data and the main conclusions easily accessible to the readers.

All other issues raised by the reviewers need to be satisfactorily addressed as well. As you may already know, our editorial policy allows in principle a single round of major revision, and it is therefore essential to provide responses to the reviewers' comments that are as complete as possible.

On a more editorial level, we would ask you to address the following issues:

Reviewer #1:

Bacteria engineered to secrete anti-inflammatory cytokines have applications as next-generation therapeutics. The authors have previously engineered the pathogen M. pneumoniae as a protein secretion chassis for therapeutic applications in the lungs. Here, the authors substantially advance their previous work by i) engineering M. pneumoniae to secrete the potent homodimeric anti-inflammatory cytokine IL-10, ii) using a structure-guided approach to identify mutations that increase IL-10 activity, iii) engineering novel single-chain IL-10 mutants that are secreted by M. pneumoniae to higher levels and have improved activity, iv) developing a novel P. aeruginosa mouse model of lung inflammation in order to test the therapeutic efficacy of their strains, vi) engineering a novel M. pneumoniae strain with substantially reduced pathogenicity to use for therapeutic purposes. They provide evidence that their approach results in strong attenuation of inflammation in the lungs. These results could be advanced toward new therapeutics for inflammatory diseases of the lungs, including pneumoniae or viral (e.g. SARS-CoV2) infections. Their single-chain IL10 molecules could also have other therapeutic applications.

The manuscript is innovative and impactful. I believe that it is appropriate for publication in Molecular Systems Biology, pending a number of improvements to the presentation and several key additional experiments.

Specific comments
Please add line numbers.

The IL-10 functionality experiment in Figure 1 did not work well. Treatment of cells with positive control (recombinant human IL-10) results in a statistically significant change in only one of the seven inflammation biomarkers tested (Figure 1a). Similar results are seen in the bacterial-produced IL-10 experiments (Figure 1b). The authors do observe expected changes in the marker levels, but additional replicates are required to achieve statistically significance.

No information is given in the main text, figure legend, nor methods section about how the bacterial supernatant-human cell treatment experiment was done. A detailed methodological description of this experiment should be added.

From the main text, it sounds like only a single mutation was introduced into IL-10 Mut1 and Mut2. The descriptions of the design of these mutants should be clarified.

I do not understand the statement "We checked that the mutations were independent of the structure used by...." on page 6. The authors are describing part of their high affinity IL-10 computational design procedure, but the approach is not clear. This statement should be clarified.

How many and which mutations were introduced into IL-10 to generated Mut3 is not clear (ambiguous) based on the main text description. This information should be clarified in the main text.

The scale of the axis in Figure 3a is wrong (EC-50 values of 10^10).

The claim that the measured affinity of MutM is lower than that of IL-10 ORF is not supported by the data (Figure 3A). In particular, the two are statistically equivalent.

Why was Mut3 better than Mut2 or Mut 1? The authors do not provide any interpretation of their observations. Even if somewhat speculative, a hypothesis should be given.

The MutSC1 and MutSC2 (currently in Figures 3B-D) should not be shown before the design of MutSC1 and MutSC2 (currently Figure 4). This anachronistic presentation makes the manuscript more difficult to follow.

The description of how MutSC1 was designed is insufficient to understand both the molecule itself and the methods that were used to design it. For example, there is discussion of a linker sequence but where that sequence is introduced in the molecule is not described. As a second example, I am not familiar with "side chain repair" or why that would be utilized to design MutSC1. Figure 4 provides some of the information, but does not completely address these issues.

The authors report the efficacy of MutSC1 and MutSC2 by comparing their EC50 values to that of IL-10 ORF. However, the IL-10 ORF data is absent from the relevant main text figure (Figures 3C and D). The IL-10 ORF data should be plotted against the MutSC1 and MutSC2 data.

"One fold better" is not a common way to compare two data sets and it suggests that two things behave the same way. Perhaps the authors mean to say 100% greater?

The authors do not characterize the secretion levels of IL-10 ORF, MutSC1 or MutSC2 from CV8. This experiment must be performed in order to demonstrate that their virulence-attenuated M. pneumoniae strain does not have compromised protein production or secretion capacity. This is particularly important, because the authors observe no anti-inflammatory effect from IL-

10 using their attenuated chassis in their P. aeruginosa model (Figure 6c). One possible explanation for this is that CV8 does not secrete as much IL-10 ORF as the non-attenuated strain.

Reviewer #2:

In this work, Montero-Blay et al. propose an engineered living therapeutic aimed at addressing inflammatory conditions in the lung. For this, they create an attenuated strain of M. pneumoniae that expresses engineered versions IL-10. While single-chain IL-10 versions had been engineered previously, the authors rationally designed new versions of single-chain IL-10 with enhanced properties that might prove useful to address the challenges inherent to the lung environment and the use of MPN as a therapeutic chassis. The authors then use a mouse model of P. aeruginosa infection to evaluate the efficacy of the engineered bacteria in ameliorating inflammation in the lungs and present data that suggests their new versions of IL-10 display enhanced anti-inflammatory effect.

- I have a major concern about the data derived from the mouse studies. The authors do not report the exact number of animals used per group nor whether they performed (more than one) independent experiments to support the validity of the data. They only present a vague reference in the methods section that states: "Infections were performed in groups of at least 5 mice per strain (n {greater than or equal to} 5)". Without the sample size information, it is difficult to assess the significance of the data and it undermines the interpretation of the results. The authors should state the exact number of mice used in each group and present the data as individual data points along with the appropriate measure of dispersion. Mouse studies that support major claims of the work should be performed at least twice in independent experiments.

- Structuring and writing of the manuscript should be improved. The last paragraph of the introduction section presents results and interpretations before the data is shown. Methods and Discussion sections also report experimental results. Why is figure 4 presented in the text before addressing Fig 3C and D? Please reorganize text and figures to follow the logical flow of a scientific article.

- "Afterwards, we mutated the Phe after N157 to Asn to improve binding to R1 (this results in NGGLD as the inserted sequence; Fig. 4A); this improved the overall stability of the SC molecule as well as its binding to R1." Please state the metric used and present the data that supports the claim of improved stability.

- "Regarding the CV8 strain, we could see a minor inflammatory response when it was used alone (Fig. 6C), ...". Please describe what constitutes a "minor" inflammatory response. This sort of qualification seems subjective.

- "...as a drug factory that is easy to remove with antibiotics once its action is completed.". This statement should be revised. No data is presented supporting the efficacy of antibiotics in removing CV8 and related strains from the lungs. Antibiotic treatment should not be considered as biocontainment strategy due to the undesired effects that it might have i.e., emergence of resistant variants, dysbiosis, etc. Further discussion of an effective biocontainment strategy for this lung-targeted living therapeutic should be included.

Reviewer #3:

The manuscript submitted by Ariadna Montero-Blay and colleagues is divided into two main themes: 1) the rational engineering of IL-10 using computational protein design tools, and 2) the development of the Mycoplasma pneumoniae CV8 live biotherapeutic cell chassis and its activity in a mouse model of lung infection. Different versions of IL-10 were prepared including surface mutations and single-chain conformations (given that IL-10 forms intra-monomer disulfide bonds). The level of secretion by the CV8 chassis and the activity of some of the IL-10 variants were increased in vitro and displayed nice anti-inflammatory properties in mice infected with Pseudomonas.

The manuscript is a nice demonstration of the potential of live biotherapeutics and how rational engineering of active molecules can potentiate their effects. The work is following a recently published article in Molecular Systems biology in which a M. pneumoniae live biotherapeutic chassis was presented. The key difference is the molecule (IL-10) that was secreted and the organ that is treated (lung) while the previous work was focussing on biofilm and catheters.

Overall, I found the work to be interesting and well performed. However, some sections can be confusing and would require rewording or clarifications. I would encourage the authors to use line numbers in future manuscripts to facilitate the review process.

1) Rewording a few sentences would enhance clarity and avoid misinterpretation.

-Activity of IL-10 derivatives; a few sentences suggest that the activity of the single-chain IL-10 shows a 60-fold increase in activity (abstract line 9; page 13, line 12. This can be misleading as the result is a combination of increased secretion by Mycoplasma pneumoniae and better activation of the receptor. There is no head-to-head comparison of purified single-chain IL-10 derivative with WT IL-10 at the same concentration that would support this conclusion. These sentence should be reworded to avoid any misunderstanding.

-page 3, line 21. The sentence about the selection of a live biotherapeutic chassis with regards to the organ to treat could be reworded to improve syntax and clarity.

2) the information in certain Supplementary Tables is difficult to understand without additional details:

-page 12, second paragraph, lines 7-8: it is not obvious to me that the lung is more challenging than intestine for live biotherapeutics. The authors could clarify their thoughts. I believe they meant that fewer bacteria are present in this organ but there are other factors to consider and is not as simple as this.

-Supplementary Table 1: It is difficult to understand why a correction factor was applied and how this number was determined. This should be explained.

-Supplementary Table 2: why is the format of the data different in the "sulfide MutSC1" tab compared to "Sulfide WT IL-10" and "Sulfide MutSC2"?

Minor:

-page 11, last line: 2 ug instead of 2 ugr.

-page 15, first sentence of the last paragraph: ATCC instead of ATTC.

-page 25, line 6. Bmax should be defined at first use.

-Supplementary Table 6: the "Inflammatory makers" tab should be "Inflammatory markers"

-Supplementary Table 10: why is only the amino acid sequence provided for IL-10 derivatives? DNA sequences would ideally also be reported.

Reviewer #1:

We would like to thank you for your critical revision of our manuscript ("Bacterial expression of a designed single-chain IL-10 prevents lung severe inflammation"). We have taken your comments in consideration and have now addressed all the issues you raised. (Note that new changes in the manuscript text shown here are highlighted in blue).

Bacteria engineered to secrete anti-inflammatory cytokines have applications as next-generation therapeutics. The authors have previously engineered the pathogen M. pneumoniae as a protein secretion chassis for therapeutic applications in the lungs. Here, the authors substantially advance their previous work by i) engineering M. pneumoniae to secrete the potent homodimeric anti-inflammatory cytokine IL-10, ii) using a structure-guided approach to identify mutations that increase IL-10 activity, iii) engineering novel single-chain IL-10 mutants that are secreted by M. pneumoniae to higher levels and have improved activity, iv) developing a novel P. aeruginosa mouse model of lung inflammation in order to test the therapeutic efficacy of their strains, vi) engineering a novel M. pneumoniae strain with substantially reduced pathogenicity to use for therapeutic purposes. They provide evidence that their approach results in strong attenuation of inflammation in the lungs. These results could be advanced toward new therapeutics for inflammatory diseases of the lungs, including pneumoniae or viral (e.g. SARS-CoV2) infections. Their single-chain IL10 molecules could also have other therapeutic applications.

The manuscript is innovative and impactful. I believe that it is appropriate for publication in Molecular Systems Biology, pending a number of improvements to the presentation and several key additional experiments.
Specific comments

Please add line numbers.

Added

The IL-10 functionality experiment in Figure 1 did not work well. Treatment of cells with positive control (recombinant human IL-10) results in a statistically significant change in only one of the seven inflammation biomarkers tested (Figure 1a). Similar

results are seen in the bacterial-produced IL-10 experiments (Figure 1b). The authors do observe expected changes in the marker levels, but additional replicates are required to achieve statistical significance.

We thank the reviewer for this comment. The main point of this experiment was to demonstrate that IL-10 expressed by Mycoplasma is active and capable of modulating the response by macrophages. In the submitted data, results of four independent donors were presented as bars in a plot. These data were not normalized; rather, mean fluorescence intensity (MFI) values for the donors (n=4) are shown. As the data were not normalized, the statistical difference of controls and treated cells with commercial and Mycoplasma-expressed IL-10 was not significant for many of the biomarkers, even though all donors showed similar trends of up- and downregulation of them.

To solve this issue, and to address the reviewer's comment, we have made a new Figure 1, in which:

i) each donor is represented as an individual point; and

ii) data have been normalized using the MFI values obtained in the control group (now in the new Supplementary Table EV1).

The results now reveal a significant effect of the Mycoplasma secreted IL-10 (WT_IL10 supernatant) on the anti-inflammatory CD163, CD16, and PDL1 markers, as well as a tendency for MerTK. In addition, we also observed the statistical significance of the downregulation of the pro-inflammatory markers MHC-II and CD86.

No information is given in the main text, figure legend, nor methods section about how the bacterial supernatant-human cell treatment experiment was done. A detailed methodological description of this experiment should be added.

We have now restructured the Methods and provided further information. Specifically, the experimental details given in the 'Blood monocyte assays' section are now organized into two following subsections: "Blood monocyte Isolation" and "IL-10 stimulation and analysis by flow cytometry". This modification appears in the manuscript in lines 420-454.

From the main text, it sounds like only a single mutation was introduced into IL-10 Mut1 and Mut2. The descriptions of the design of these mutants should be clarified.

We apologize for the confusion. Mut1 and Mut2, as well as the rest of the mutants, are fully described in Table EV9. We have now added the amino acid compositions, DNA sequences, and specific details for each mutant.

The specific set of mutations introduced in IL-10 for Mut1 and Mut2 are the following:

Mut1: D28E,S31R,N45S,T155M

Mut2: D28E,S31K,N45S,T155M

The difference between these two mutants is at position 31, where Mut1 has an Arg and Mut2 a Lys. This information is included in lines 121-123 as follows:

Using the selected mutations, we modeled two multiple mutants (Mut1 and Mut2), which contain the mutations D28E,S31K/R,N45S,TL155M; note that these differ at position 31, whereby the WT Ser31 is mutated to Lys in Mut1 and Arg in Mut2 (Fig. 2).

I do not understand the statement "We checked that the mutations were independent of the structure used by...." on page 6. The authors are describing part of their high affinity IL-10 computational design procedure, but the approach is not clear. This statement should be clarified.

This is a complex issue to describe. In order to be more precise, we have added the corresponding free energies for PDB=1j7v in Table EV3 and modified the paragraph in the revised version, which we hope is clearer.

"We verified that the differences in stability and binding energies were not the result of a particular conformation of the crystal structure that we used by computing the in silico variation of free energies of the multiple mutations of IL-10 in Mut1 and Mut2 using other experimental structures (IL-10 apo form: PDB 2ilk, 1.6 Å; IL-10 holo form with R1: PDB 1j7v, 2.9 Å; IL-10 holo form with R1&R2: PDB 6x93, 3.5 Å). We looked for changes in energy that affected IL-10 stability, interactions with R1 and R2 (when present in the PDB structure), and stability of IL-10 receptor complex (Table EV3)."

(Lines 123-129)

How many and which mutations were introduced into IL-10 to generate Mut3 is not clear (ambiguous) based on the main text description. This information should be clarified in the main text.

As stated in the previous point, we apologize for the confusion.

Mut 3 is the result of introducing R2-enhancing affinity mutations on top of those introduced in Mut2. Therefore, the composition of the mutant is D28E, S31K, N45S, T155M, N18I, N92I, K99N. This information is now clarified in the text in lines 130-133 and in Table EV9.

At the start of this work, no structure was available of IL-10 bound to R2. Therefore, we included mutations in Mut2 at some positions previously reported to potentially enhance the affinity of the interactions between IL-10 and R2 (e.g., N18I, N92I, and K99N) (Gorby et al, 2020), resulting in Mut3.

The scale of the axis in Figure 3a is wrong (EC-50 values of 10^10).

Thanks for pointing this out; we have now corrected this typo in the new version (new Fig 4A).

The claim that the measured affinity of MutM is lower than that of IL-10 ORF is not supported by the data (Figure 3A). In particular, the two are statistically equivalent.

MutM was described previously (Josephson *et al.*, 2000). In that work, they analyzed the activity of MutM (stoichiometry of interaction of 1:1:1 with IL-10R1 and IL-10R2). They mentioned that this mutant was less active than the IL-10 WT (stoichiometry of interaction of 1:2:2). We find a similar tendency (although not statistically significant, p-value > 0.05). We accepted the Reviewer suggestion, and we have changed the text as follows (lines 157-159):

We showed that the EC-50 of the monomeric IL-10 (MutM) is on average lower (although not statistically significant, p-value > 0.05) than that of the IL-10 ORF (2.56 e-10 +/- 8.77 e-11), as previously described (Josephson et al, 2000) (Fig. 4A and Table EV4).

Why was Mut3 better than Mut2 or Mut 1? The authors do not provide any interpretation of their observations. Even if somewhat speculative, a hypothesis should be given.

Mut 3 has three mutations that enhance IL-10R2 interaction plus the Mut2 mutations designed to enhance IL10R1 interaction. Therefore, Mut3 has enhanced affinity towards both receptors, and therefore the affinity for IL-10 receptors was higher.

Thanks for the suggestion; we have now added this to the text, in lines 151-157.

Mut3, based on Mut2, incorporates new mutations previously described to improve R2 binding (namely N18I, N92I, and K99N; Gorby et al, 2020). Inclusion of these three mutants resulted in an improved EC-50 value (2.61 e-^{11} ± 1.49 e-11 M) with 8-fold enhancement in relative affinity as compared to the IL10 ORF (Fig. 4A). This is expected, as it contains mutations that enhance both R1 and R2 binding, while Mut2 only has mutations to enhance R1 binding.

The MutSC1 and MutSC2 (currently in Figures 3B-D) should not be shown before the design of MutSC1 and MutSC2 (currently Figure 4). This anachronistic presentation makes the manuscript more difficult to follow.

According to the reviewer's suggestion, we have switched the order of the Figures 3 and 4, and reassigned the references in the manuscript accordingly.

The description of how MutSC1 was designed is insufficient to understand both the molecule itself and the methods that were used to design it. For example, there is discussion of a linker sequence but where that sequence is introduced in the molecule is not described. As a second example, I am not familiar with "side chain repair" or why that would be utilized to design MutSC1. Figure 4 provides some of the information, but does not completely address these issues.

We have modified the single-chain IL-10 rational design section to make it more understandable and we have added the monomer descriptors in Figure 3 to accompany the changes (lines 387-395).

MutSC1 was generated in several steps: residues 12'–17' (note that monomer 2 residues and atoms are depicted by ') were deleted, as they were pointing away from the C'-terminal and were not in contact with R1; and residues 18'–156' were renumbered as 169–307 and were joined into one molecule, creating sewing pattern 1. As the best anchoring points, the residues 147 (Ile) and 169 (Residue 18′ in monomer 2, Asn) were selected and then connected using a 17-aa fragment from PDB 2wyu (residues 73–89). The original sequence in 2wyu of the chosen bridge segment was LDALFAGVKEA-FGGLD-Y. Residues LDALFAGVKEA and the last Y structurally superimposed with IL-10. Therefore, all residues were mutated back to the original IL-10 sequence unless the newly-added residues improved protein stability (Asn169 to Tyr).

We have explained what side-chain repair does in Methods (368-372):

"RepairPDB identifies residues that have bad torsion angles, VanderWaals' clashes, or total energy, and mutates them and their neighbours to themselves, exploring different rotamer combinations to find new energy minima"

Since we have grasped a peptide fragment from the ModelX database of peptide fragments to link the N- and C-termini of IL-10, we bring the original rotamers found in the structure from where the fragment was taken. These rotamers are not necessarily the same or they will require some small torsional moves in order to be accommodated in the context of the IL-10 structure. For this reason we use the RepairPDB FoldX command, to try to accomodate side chains of the fragment and of the surrounding residues of IL-10.

The authors report the efficacy of MutSC1 and MutSC2 by comparing their EC50 values to that of IL-10 ORF. However, the IL-10 ORF data is absent from the relevant main text figure (Figures 3C and D). The IL-10 ORF data should be plotted against the MutSC1 and MutSC2 data.

In Figure 3C and D, we plot the ratios in EC-50 between the designed IL-10 variants and IL-10 WT (IL-10 ORF) to indicate the fold improvement (The original data are presented in Table EV5). In order to clarify this information, we have modified the figure legend and axis and included the standard deviation in the figure to make this

clear. We think this is the best way to normalize the results. However, if the reviewer thinks that we should put the original data we are happy to change the figure.

"One fold better" is not a common way to compare two data sets and it suggests that two things behave the same way. Perhaps the authors mean to say 100% greater?

We agree with the reviewer's suggestion and have now modified the text following this suggestion (line 340-341 and line 215).

In all cases, we found that MutSC2 had a 100% higher relative affinity than MutSC1.

As seen with HEK-Blue^{TM} cells, MutSC2 was around 100% better than MutSC1

The authors do not characterize the secretion levels of IL-10 ORF, MutSC1 or MutSC2 from CV8. This experiment must be performed in order to demonstrate that their virulence-attenuated *M. pneumoniae* strain does not have compromised protein production or secretion capacity. This is particularly important, because the authors observe no anti-inflammatory effect from IL-10 using their attenuated chassis in their *P. aeruginosa* model (Figure 6c). One possible explanation for this is that CV8 does not secrete as much IL-10 ORF as the non-attenuated strain.

We have added a new set of experiments that provide evidence for the similar protein secretion capacity of the CV8 and WT *M. pneumoniae* strains (new Figure 5, panel D). We found no statistical significant differences when comparing expression of IL-10 WT and the MutSC1 and MutSC2 variants in the WT or CV8 strains.

We have added a new sentence in the manuscript in lines 249-250

No differences in protein capacity were observed between WT-expressing and CV8-expressing strains (Figure 5D).

Reviewer #2:

We would like to thank you for your critical revision of our manuscript ("Bacterial expression of a designed single-chain IL-10 prevents lung severe inflammation"). We

have taken your comments in consideration and have now addressed all the issues you raised. (Note that new changes in the manuscript text shown here are highlighted in blue).

In this work, Montero-Blay et al. propose an engineered living therapeutic aimed at addressing inflammatory conditions in the lung. For this, they create an attenuated strain of M. pneumoniae that expresses engineered versions IL-10. While single-chain IL-10 versions had been engineered previously, the authors rationally designed new versions of single-chain IL-10 with enhanced properties that might prove useful to address the challenges inherent to the lung environment and the use of MPN as a therapeutic chassis. The authors then use a mouse model of P. aeruginosa infection to evaluate the efficacy of the engineered bacteria in ameliorating inflammation in the lungs and present data that suggests their new versions of IL-10 display enhanced anti-inflammatory effect.

- I have a major concern about the data derived from the mouse studies. The authors do not report the exact number of animals used per group nor whether they performed (more than one) independent experiments to support the validity of the data. They only present a vague reference in the methods section that states: "Infections were performed in groups of at least 5 mice per strain (n {greater than or equal to} 5)". Without the sample size information, it is difficult to assess the significance of the data and it undermines the interpretation of the results. The authors should state the exact number of mice used in each group and present the data as individual data points along with the appropriate measure of dispersion. Mouse studies that support major claims of the work should be performed at least twice in independent experiments.

We agree with this comment. We understand that this suggestion concerns the mice assay performed to test the immunomodulatory effect of IL-10 single chains MutSC1 and MutSC2. To address this comment:

i) We repeated the assay with n=5 additional mice per group, and the new results confirmed our previous results. We have added this new experiment to the new Table EV7 as well as to the new Figure 6 (whereby results from experiment 1 are indicated by circles, and experiment 2, by squares).

ii) Each mouse is represented as an individual point (see new Figure 6).

iii) We have now added the number of mice used in this study in the Material and Method section, a Supplementary Table (new Table EV7) showing the mice used in the previous version (experiment 1) and the replica (experiment 2), and the criteria of exclusion. For the latter: 1) only animals with PAO1 and/or *M. pneumoniae* recovery (log >0.52) were considered; 2) animals with technical issues (*i.e.* contaminated plates) were excluded; and 3) for qPCR analysis, samples with total RNA concentration >100 ng/ul of total RNA extraction and ratio A260/A230 1.8-2.2 were considered.

iv) Considering that we are interested in the effect of the CV8 chassis coding for the designed IL-10 molecules, we showed the statistical comparison between the PAO1 + CV8 reference group and the PAO1 + CV8_IL10, PAO1 + CV8_MutSC1, PAO1 + CV8_MutSC2 and PAO1 + hIL10r groups in the new Figure 6. In addition, we included the sheet “p value” in the new Table EV7, in which we specified all the statistical values, including the comparison with the PAO1 + PBS, that present the same tendency.

We appreciate this comment because it has reinforced our previous statements and the quality of the work.

- Structuring and writing of the manuscript should be improved. The last paragraph of the introduction section presents results and interpretations before the data is shown. Methods and Discussion sections also report experimental results. Why is figure 4 presented in the text before addressing Fig 3C and D? Please reorganize text and figures to follow the logical flow of a scientific article.

We agree with the reviewer's suggestion and have switched the order of the Figure 3 and Figure 4 and reassigned the references in the manuscript accordingly. The Figure EV4 is now cited in the Results section. Also, we removed the sections that introduce results and interpretations from the introduction section.

We have now had the manuscript corrected by Dr. Veronica Raker, a professional scientific English editor (https://rocketreach.co/veronica-raker-email_51858899).

- "Afterwards, we mutated the Phe after N157 to Asn to improve binding to R1 (this results in NGGLD as the inserted sequence; Fig. 4A); this improved the overall stability of the SC molecule as well as its binding to R1." Please state the metric used and present the data that supports the claim of improved stability.

We have modified the paragraph to explain that claim (lines 180-183) as follows:

"Afterwards, we mutated the Phe after N157 to Asn to improve binding to R1 (this results in NGGLD as the inserted sequence; Fig. 3A); this improved the FoldX-predicted overall stability of the SC molecule, creating an intramolecular HBond with G160, rigidifying the inserted loop, and consequently its binding to R1 by stabilising the bound state."

- "Regarding the CV8 strain, we could see a minor inflammatory response when it was used alone (Fig. 6C), ...". Please describe what constitutes a "minor" inflammatory response. This sort of qualification seems subjective.

We agree with this comment and have replaced this line in the reviewed manuscript by (lines 271-274):

"The expression of the inflammatory markers in mice after infection with the CV8 strain was comparable that in the PBS-treated control mice (Fig. 6C)"

- "...as a drug factory that is easy to remove with antibiotics once its action is completed." This statement should be revised. No data is presented supporting the efficacy of antibiotics in removing CV8 and related strains from the lungs. Antibiotic treatment should not be considered as a biocontainment strategy due to the undesired effects that it might have i.e., emergence of resistant variants, dysbiosis, etc. Further discussion of an effective biocontainment strategy for this lung-targeted living therapeutic should be included.

We understand the reviewer's concern. Recently, gene switches to limit *M. pneumoniae* growth (biosafety) for biomedical applications have been developed (https://doi.org/10.1038/s41467-022-29574-0). The biocontainment system described in that paper could be implemented in our chassis CV8 without potentially impacting the synthesis capacity of the product. We have included this in the discussion (lines 303-306).

In fact, gene switches to limit M. pneumoniae growth for biosafety applications have been developed (Broto et al. 2022). The biocontainment system described in that paper could be implemented in our chassis CV8 without potentially impacting the synthesis capacity of the product.

Reviewer #3:

We would like to thank you for your critical revision of our manuscript ("Bacterial expression of a designed single-chain IL-10 prevents lung severe inflammation"). We have taken your comments in consideration and have now addressed all the issues you raised. (Note that new changes in the manuscript text shown here are highlighted in blue).

The manuscript submitted by Ariadna Montero-Blay and colleagues is divided into two main themes: 1) the rational engineering of IL-10 using computational protein design tools, and 2) the development of the Mycoplasma pneumoniae CV8 live biotherapeutic cell chassis and its activity in a mouse model of lung infection. Different versions of IL-10 were prepared including surface mutations and single-chain conformations (given that IL-10 forms intra-monomer disulfide bonds). The level of secretion by the CV8 chassis and the activity of some of the IL-10 variants were increased in vitro and displayed nice anti-inflammatory properties in mice infected with Pseudomonas.

The manuscript is a nice demonstration of the potential of live biotherapeutics and how rational engineering of active molecules can potentiate their effects. The work is following a recently published article in Molecular Systems biology in which a M. pneumoniae live biotherapeutic chassis was presented. The key difference is the molecule (IL-10) that was secreted and the organ that is treated (lung) while the previous work was focussing on biofilm and catheters.

Overall, I found the work to be interesting and well performed. However, some sections can be confusing and would require rewording or clarifications. I would encourage the authors to use line numbers in future manuscripts to facilitate the review process.

1) Rewording a few sentences would enhance clarity and avoid misinterpretation.

-Activity of IL-10 derivatives; a few sentences suggest that the activity of the single-chain IL-10 shows a 60-fold increase in activity (abstract line 9; page 13, line 12. This can be misleading as the result is a combination of increased secretion by *Mycoplasma pneumoniae* and better activation of the receptor. There is no head-to-head comparison of purified single-chain IL-10 derivative with WT IL-10 at the same concentration that would support this conclusion. These sentence should be reworded to avoid any misunderstanding.

Indeed, it is true that for the HEK-Blue™ cells, the "60-fold" reflects a combination of expression and change in affinity. However, when looking at the FACS analysis, we see a 29-fold improvement in EC-50 for MutSC1, and a 57-fold improvement for MutSC2 in BLAeR cells. We have slightly modified the abstract to make this clearer.

'As compared to the IL-10 WT, the designed SC-IL10 molecules increase the effective expression in *MPN* four-fold, and the activity in mouse and human cell lines between 10- to 60-times, depending on the cell line'

However, the reviewer is right that the sentence in Page 13, line12 is not correct

Combining the higher expression and the better EC-50 shown in the HEK-Blue™ cell line, the WT *Mycoplasma* strain expressing MutSC2 has around 60 times **relatively higher relative affinity** *in vitro* than the IL-10 ORF strain

Thus we have replaced it by (lines 333-336):

'Combining the higher expression and the better EC-50 shown in the HEK-Blue™ cell line, the supernatant of the WT *Mycoplasma* strain expressing MutSC2 has around 60-times higher effect *in vitro* on HEK-Blue™ cells than the supernatant of the IL-10 ORF strain'.

-page 3, line 21. The sentence about the selection of a live biotherapeutic chassis with regards to the organ to treat could be reworded to improve syntax and clarity.

To improve the clarity of this point, we have re-structured the text. Now it appears as following in lines 41-46:

'The use of bacteria as delivery vectors or live biotherapeutic (LB) has the advantage of being able to produce the therapeutic agent locally, which lowers the required administered dose, thereby reducing the adverse effects and the production costs. Recently, IL-10 expressed by Lactococcus lactis has been shown to be effective in preventing and treating colitis in a preclinical mice model (Steidler et al, 2000a; Cardoso et al, 2018) and also in phase I clinical trials of Crohn's disease (Braat et al, 2006).'

2) the information in certain Supplementary Tables is difficult to understand without additional details:

-page 12, second paragraph, lines 7-8: it is not obvious to me that the lung is more challenging than the intestine for live biotherapeutics. The authors could clarify their thoughts. I believe they meant that fewer bacteria are present in this organ but there are other factors to consider and it is not as simple as this.

We agree with this comment and have now modified the corresponding discussion section (lines 317-320):

"This choice was made based on reports showing that exogenous delivery of IL-10 ameliorates different lung diseases (Cypel et al, 2009; Shamskhou et al, 2019; Lindner et al, 2021) and because the lung is a challenging organ due to its reduced microbiome size (Huffnagle et al, 2017)"

-Supplementary Table 1: It is difficult to understand why a correction factor was applied and how this number was determined. This should be explained.

We apologize for the confusion. Protein expression can vary across biological

conditions because of differences in biomass or incubation time. Plus, the method used for biomass determination (CFU) or IL-10 quantification (ELISA) has a certain degree of variability between samples that increases when the production of IL-10 in fg per cell (CFU) is calculated. In the previous manuscript, we selected the number of molecules per cell for MutSC1 obtained in the replicate 'R2' as a correction factor to standardize the results. However, we believe that the normalization by the protein production per CFU (fg IL-10/CFU) of the reference IL-10 ORF is more appropriate. For this, we have now ,calculated the ratio ORF/ORF (=1), Mut3/ORF and MutSC1/ORF for each experiment (see new Table EV1). We observed that in all the experiments, the production of MutSC1 is 2.96x, 13.29x or, 2.25x of that produced from protein ORF, while the Mut3 production was comparable to the ORF in all the replicas analyzed. We have now modified panel B in the new Fig. 4 accordingly.

-Supplementary Table 2: why is the format of the data different in the "sulfide MutSC1" tab compared to "Sulfide WT IL-10" and "Sulfide MutSC2"?

The goal of the experiment is to identify the disulfide bridges in the WT IL-10, MutSC1 and MutSC2 proteins.

In the first experimental setting using MS, we clearly identified the cysteine12 that is carbamylated in IL-10 WT or MutSC2. However, this could not be precisely identified for MutSC1 (see Table EV2), likely due to the fact that peptides obtained after enzyme digestion were not optimal for MS detection. To improve this result, we performed an additional experiment for the MutSC1 to double-confirm the presence of carbamidomethylate in the first cysteine (Cys12) using PRM (Parallel Reaction Monitoring) chromatography followed by MS. This is the reason for the different formats of describing these (e.g., "Sulfide WT IL-10", "Sulfide MutSC2", and "Sulfide MutSC1") in the previous version. We have now reorganized the data in the new Supplementary Table EV2 in the following sheets: "Sulfide WT IL-10 MS", "Sulfide MutSC2 MS", "Sulfide MutSC1 MS" and "Sulfide MutSC1 PRM + MS".
Minor:

-page 11, last line: 2 ug instead of 2 ugr.
-page 15, first sentence of the last paragraph: ATCC instead of ATTC.

We thank the reviewer for seeing these and have now corrected them.

-page 25, line 6. Bmax should be defined at first use.

We agree and have changed it.

-Supplementary Table 6: the "Inflammatory makers" tab should be "Inflammatory markers"

We have corrected it.

-Supplementary Table 10: why is only the amino acid sequence provided for IL-10 derivatives? DNA sequences would ideally also be reported.

We agree and have now modified Supplementary Table EV9 to provide the DNA sequence plus the amino acid sequence and the specific details for each mutant.

Thank you for sending us your revised manuscript. We have now received the comments from the three referees who were asked to re-review your manuscript. As you will see below, the referees are satisfied with the modifications and think the study is now suitable for publication.

Before we can formally accept your manuscript, we would ask you to address the following issues:

1. The remaining minor comments of Referee #3.

Reviewer #1:

I thank the authors for carefully addressing each of my previous comments. I believe the manuscript is suitable for publication.

Reviewer #2:

The authors have done an excellent job in addressing the points raised in our original review. We recommend the revised paper for publication in MSB.

Reviewer #3:

This manuscript is a resubmission from Montero-Blay and colleagues in response to comments made by three reviewers a few months ago. The authors made several changes and additions that significantly improved the manuscript. Overall, I am satisfied with the updated version, but I would suggest a few minor modifications listed below to further improve the manuscript before publication.

Suggestions for minor modifications:

-I would suggest editing line 17 (abstract) to add "also": "IL-10 can ALSO be used as a treatment of a wide range of pulmonary diseases" to smoothen the transition between gut inflammation and pulmonary disease.

-I would suggest editing line 26 (abstract) to "This rational design strategy could be applied to other molecules..." instead of "This rational design strategy could be used to other molecules ».

-The use of "LB" as an abbreviation for "live biotherapeutics" is the same as LB medium used later in the text. I know the context should help make the distinction between the two. Still, I would suggest using "LBT" instead for "Live BioTherapeutics" or "LBP" for "Live Biotherapeutics Products" as already published in other articles.

-Please ensure that the EV files are labeled as such within the files themselves (e.g.: Table EV1 instead of Supplementary Table 1).

Line 302: This sentence could be reworded for increased readability. Suggestion: "The use of live biotherapeutics that incorporate biocontainment strategies and secrete IL-10 could...".

2nd Revision - Editorial Decision 5th Oct 2022

Manuscript number: MSB-2022-11037RR
Title: Bacterial expression of a designed single-chain IL-10 prevents severe lung inflammation

Thank you again for sending us your revised manuscript. We are now satisfied with the modifications made and I am pleased to inform you that your paper has been accepted for publication.

EMBO Press Author Checklist

Corresponding Author Name: Luis Serrano Pubul	
Journal Submitted to: Molecular Systems Biology (MSB)	
Manuscript Number: MSB-2022-11037R	

USEFUL LINKS FOR COMPLETING THIS FORM
The EMBO Journal - Author Guidelines
EMBO Reports - Author Guidelines
Molecular Systems Biology - Author Guidelines
EMBO Molecular Medicine - Author Guidelines

Reporting Checklist for Life Science Articles (updated January
This checklist is adapted from Materials Design Analysis Reporting (MDAR) Checklist for Authors. MDAR establishes a minimum set of requirements in transparent reporting in the life sciences (see Statement of Task: 10.31222/osf.io/9sm4x). Please follow the journal's guidelines in preparing your
Please note that a copy of this checklist will be published alongside your article.

Abridged guidelines for figures
1. Data
The data shown in figures should satisfy the following conditions:
- the data were obtained and processed according to the field's best practice and are presented to reflect the results of the experiments in an accurate and unbiased manner.
- ideally, figure panels should include only measurements that are directly comparable to each other and obtained with the same assay.
- plots include clearly labeled error bars for independent experiments and sample sizes. Unless justified, error bars should not be shown for technical
- if n<5, the individual data points from each experiment should be plotted. Any statistical test employed should be justified.
- Source Data should be included to report the data underlying figures according to the guidelines set out in the authorship guidelines on Data

2. Captions
Each figure caption should contain the following information, for each panel where they are relevant:
- a specification of the experimental system investigated (eg cell line, species name).
- the assay(s) and method(s) used to carry out the reported observations and measurements.
- an explicit mention of the biological and chemical entity(ies) that are being measured.
- an explicit mention of the biological and chemical entity(ies) that are altered/varied/perturbed in a controlled manner.
- the exact sample size (n) for each experimental group/condition, given as a number, not a range;
- a description of the sample collection allowing the reader to understand whether the samples represent technical or biological replicates (including how many animals, litters, cultures, etc.).
- a statement of how many times the experiment shown was independently replicated in the laboratory.
- definitions of statistical methods and measures:
  - common tests, such as t-test (please specify whether paired vs. unpaired), simple χ2 tests, Wilcoxon and Mann-Whitney tests, can be unambiguously identified by name only, but more complex techniques should be described in the methods section;
  - are tests one-sided or two-sided?
  - are there adjustments for multiple comparisons?
  - exact statistical test results, e.g., P values = x but not P values < x;
  - definition of 'center values' as median or average;
  - definition of error bars as s.d. or s.e.m.

Please complete ALL of the questions below.
Select "Not Applicable" only when the requested information is not relevant for your study.

Materials

Newly Created Materials	Information included in the manuscript?	In which section is the information available? (Reagents and Tools Table, Materials and Methods, Figures, Data Availability Section)
New materials and reagents need to be available; do any restrictions apply?	Yes	Reagents and Tools table

Antibodies	Information included in the manuscript?	In which section is the information available? (Reagents and Tools Table, Materials and Methods, Figures, Data Availability Section)
For **antibodies** provide the following information:<br>- Commercial antibodies: RRID (if possible) or supplier name, catalogue number and or/clone number<br>- Non-commercial: RRID or citation	Yes	Reagents and Tools table

DNA and RNA sequences	Information included in the manuscript?	In which section is the information available? (Reagents and Tools Table, Materials and Methods, Figures, Data Availability Section)
**Short novel DNA or RNA including primers, probes**: provide the sequences.	Yes	Dataset EV5 and Table EV3

Cell materials	Information included in the manuscript?	In which section is the information available? (Reagents and Tools Table, Materials and Methods, Figures, Data Availability Section)
**Cell lines:** Provide species information, strain. Provide accession number in repository **OR** supplier name, catalog number, clone number, and/**OR** RRID.	Yes	Reagents and Tools table
**Primary cultures:** Provide species, strain, sex of origin, genetic modification status.	Not Applicable	Reagents and Tools table
Report if the cell lines were recently **authenticated** (e.g., by STR profiling) and tested for mycoplasma contamination.	Yes	Reagents and Tools table

Experimental animals	Information included in the manuscript?	In which section is the information available? (Reagents and Tools Table, Materials and Methods, Figures, Data Availability Section)
**Laboratory animals or Model organisms:** Provide species, strain, sex, age, genetic modification status. Provide accession number in repository **OR** supplier name, catalog number, clone number, **OR** RRID.	Yes	Reagents and Tools table and Material and Methods section
**Animal observed in or captured from the field:** Provide species, sex, and age where possible.	Not Applicable	
Please detail **housing and husbandry conditions**.	Yes	Material and Methods

Plants and microbes	Information included in the manuscript?	In which section is the information available? (Reagents and Tools Table, Materials and Methods, Figures, Data Availability Section)
**Plants:** provide species and strain, ecotype and cultivar where relevant, unique accession number if available, and source (including location for collected wild specimens).	Not Applicable	
**Microbes:** provide species and strain, unique accession number if available, and source.	Yes	Reagents and Tools and Dataset EV5

Human research participants	Information included in the manuscript?	In which section is the information available? (Reagents and Tools Table, Materials and Methods, Figures, Data Availability Section)
If collected and within the bounds of privacy constraints report on age, sex and gender or ethnicity for all study participants.	Not Applicable	

Core facilities	Information included in the manuscript?	In which section is the information available? (Reagents and Tools Table, Materials and Methods, Figures, Data Availability Section)
If your work benefited from core facilities, was their service mentioned in the acknowledgments section?	Yes	Acknowledgments

Design

Study protocol	Information included in the manuscript?	In which section is the information available? (Reagents and Tools Table, Materials and Methods, Figures, Data Availability Section)
If study protocol has been **pre-registered, provide DOI in the manuscript**. For clinical trials, provide the trial registration number **OR** cite DOI.	Not Applicable	
Report the **clinical trial registration number** (at ClinicalTrials.gov or equivalent), where applicable.	Not Applicable	

Laboratory protocol	Information included in the manuscript?	In which section is the information available? (Reagents and Tools Table, Materials and Methods, Figures, Data Availability Section)
Provide DOI OR other citation details if **external detailed step-by-step protocols** are available.	Not Applicable	

Experimental study design and statistics	Information included in the manuscript?	In which section is the information available? (Reagents and Tools Table, Materials and Methods, Figures, Data Availability Section)
Include a statement about **sample size** estimate even if no statistical methods were used.	Not Applicable	
Were any steps taken to minimize the effects of subjective bias when allocating animals/samples to treatment (e.g. **randomization procedure**)? If yes, have they been described?	Yes	Material and Methods
Include a statement about **blinding** even if no blinding was done.	Yes	Material and Methods (Histology)
Describe **inclusion/exclusion criteria** if samples or animals were excluded from the analysis. Were the criteria pre-established?<br><br>If sample or data points were omitted from analysis, report if this was due to attrition or intentional exclusion and provide justification.	Yes	Material and Methods
For every figure, are **statistical tests** justified as appropriate? Do the data meet the assumptions of the tests (e.g., normal distribution)? Describe any methods used to assess it. Is there an estimate of variation within each group of data? Is the variance similar between the groups that are being statistically compared?	Yes	Material and Methods

Sample definition and in-laboratory replication	Information included in the manuscript?	In which section is the information available? (Reagents and Tools Table, Materials and Methods, Figures, Data Availability Section)
In the figure legends: state number of times the experiment was **replicated** in laboratory.	Yes	Figure legends
In the figure legends: define whether data describe **technical or biological replicates**.	Yes	Figure legends

Ethics

Ethics	Information included in the manuscript?	In which section is the information available? (Reagents and Tools Table, Materials and Methods, Figures, Data Availability Section)
Studies involving **human participants**: State details of **authority granting ethics approval** (IRB or equivalent committee(s), provide reference number for approval.	Yes	Material and Methods
Studies involving **human participants**: Include a statement confirming that **informed consent** was obtained from all subjects and that the experiments conformed to the principles set out in the WMA Declaration of Helsinki and the Department of Health and Human Services Belmont Report.	Not Applicable	
Studies involving **human participants:** For publication of **patient photos**, include a statement confirming that consent to publish was obtained.	Not Applicable	
Studies involving experimental **animals**: State details of **authority granting ethics approva**l (IRB or equivalent committee(s), provide reference number for approval. Include a statement of compliance with ethical regulations.	Yes	Animal Ethics Committe (Biomedical Research Parc of Barcelona - PRBB) LSP-20-0031; Local Government: 11138
Studies involving **specimen and field samples:** State if relevant **permits** obtained, provide details of authority approving study; if none were required, explain why.	Not Applicable	

Dual Use Research of Concern (DURC)	Information included in the manuscript?	In which section is the information available? (Reagents and Tools Table, Materials and Methods, Figures, Data Availability Section)
Could your study fall under dual use research restrictions? Please check biosecurity documents and list of **select agents and toxins** (CDC): https://www.selectagents.gov/sat/list.htm	Not Applicable	
If you used a select agent, is the security level of the lab appropriate and reported in the manuscript?	Not Applicable	
If a study is subject to dual use research of concern regulations, is the name of the **authority granting approval and reference number** for the regulatory approval provided in the manuscript?	Not Applicable	

Reporting

The MDAR framework recommends adoption of discipline-specific guidelines, established and endorsed through community initiatives. Journals have their own policy about requiring specific guidelines and recommendations to complement MDAR.

Adherence to community standards	Information included in the manuscript?	In which section is the information available? (Reagents and Tools Table, Materials and Methods, Figures, Data Availability Section)
State if relevant guidelines or checklists (e.g., **ICMJE, MIBBI, ARRIVE, PRISMA**) have been followed or provided.	Not Applicable	
For **tumor marker prognostic studies,** we recommend that you follow the **REMARK** reporting guidelines (see link list at top right). See author guidelines, under 'Reporting Guidelines'. Please confirm you have followed these guidelines.	Not Applicable	
For **phase II and III randomized controlled trials**, please refer to the **CONSORT** flow diagram (see link list at top right) and submit the CONSORT checklist (see link list at top right) with your submission. See author guidelines, under 'Reporting Guidelines'. Please confirm you have submitted this list.	Not Applicable	

Data Availability

Data availability	Information included in the manuscript?	In which section is the information available? (Reagents and Tools Table, Materials and Methods, Figures, Data Availability Section)
Have **primary datasets** been deposited according to the journal's guidelines (see 'Data Deposition' section) and the respective accession numbers provided in the Data Availability Section?	Yes	Materials and Methods
Were **human clinical and genomic datasets** deposited in a public access-controlled repository in accordance to ethical obligations to the patients and to the applicable consent agreement?	Not Applicable	
Are **computational models** that are central and integral to a study available without restrictions in a machine-readable form? Were the relevant accession numbers or links provided?	Not Applicable	
If publicly available data were reused, provide the respective **data citations in the reference list**.	Not Applicable	
